# The development of an evidence-based street food vending model within a socioecological framework: A guide for African countries

**DOI:** 10.1371/journal.pone.0223535

**Published:** 2019-10-22

**Authors:** Jillian Hill, Zandile Mchiza, Thandi Puoane, Nelia P. Steyn

**Affiliations:** 1 Non-Communicable Diseases Research Unit, South African Medical Research Council, Cape Town, South Africa; 2 School of Public Health, University of the Western Cape, Cape Town, South Africa; 3 Division of Human Nutrition, University of Cape Town, Cape Town, South Africa; Embrapa Agroindústria Tropical, BRAZIL

## Abstract

In the present global economic crisis and continued rapid urbanization, street food (SF) vending has grown into a practical source of income for people in the developing world. SF are not only appreciated for their unique flavours, convenience, and affordability they also contribute to the economy of the country, the perseverance of cultural and social heritage of society, as well as the potential for maintaining and improving the nutritional status of populations. This study aimed to develop a street food vending model (SFVM) that encompasses healthy and safe food options for consumers including hygiene and safety guidelines and viable business and operations for vendors. An evidence-based approach, i.e. “systematically collected proof”, was used to inform the development of this model. Phase 1 included two surveys, one of street food vendors (N = 831) and the other of consumers (N = 1047). These surveys obtained data regarding the vendors’ operations and food items they sold and the consumers’ purchases and their nutrition knowledge. In Phase 2, interviews and focus groups were conducted with government officials. Additionally, regulations and policies regarding street vending were reviewed to determine available regulations and policies for street food vending. In Phase 3, data from the two phases were integrated and participatory action methods involving street food vendors used to validate the findings and inform the development of a SFVM by engaging in focus group discussions with street food vendors (N = 28). The components of the proposed SFVM comprised four parts, namely a food and nutrition component, a hygiene component, a business component and a vending cart. These components serve as a guide and considers various elements of the socioecological framework, namely intrapersonal/individual and interpersonal factors, the physical environment/community as well as the policy environment. The development of this model can serve as an example to countries which have large street food vending components and wish to optimize their value by making them safe and healthy for consumers. Thus, allowing vendors to trade under optimal conditions giving due consideration to regulations and policy.

## Introduction

The street food (SF) vending business as an informal employment sector has grown significantly in South Africa over the past few decades [1–3]. This has been fuelled by the fact that the formal sector cannot grow fast enough to cater for all the nations’ employment requirements [3, 4]. In the Quarterly Labour Force Survey (QLFS) of Statistics South Africa [5], the unemployment rate was estimated to have risen to 27.7% in the first quarter (January to March 2017). This rate is the highest recorded since 2003 and despite the South African government’s initiatives to grow employment in the country. Furthermore, it has been shown to be the highest among young women aged 15–34 years, the black African population, and those with an education level less than Grade 12.

In response to this high unemployment rate, it is typical that most street traders are of African origin and frequently the sole breadwinners of their families [2]. In a recent survey conducted on the informal economy of the Western Cape Province, over 1800 informal businesses were interviewed. Of these, 40% were in the food and drinks trade, with most of them (46%) making less than R1000 (83USD) profit per month [1]. Evidence exists suggesting that most African street traders are “survivalist” (subsistence) traders [6]. Willemse, 2003 [7], echoes this in stating that street trading encapsulates a survival or coping strategy for the poor to escape hunger by generating a small income. In addition to the street trade being a source of income, it also contributes significantly to the diet of many people living in developing countries, including South Africa [8]. Popular SF in South Africa include cooked food items such as “vetkoek” (fat cake/doughnut) with a protein filling of chicken liver or French polony (very popular in South Africa, consists out of highly processed meat (pork, chicken, beef), also common are high-fat meals of meat with visible fat, starch, fat and vegetables, fish and chips [9]. In the rest of Africa common SF include more traditional foods such as roasted meat, fried snails and fish, fried bean cake, steamed bean cake, roasted and fried yam and plantain, cooked or roasted maize, cassava chips, sugar cane, groundnuts, boiled eggs among others [10]. Street foods are convenient, cheap, and easily accessible [8, 11]. In South Africa, black Africans are the most regular purchasers, with nearly one out of five (19%) consuming SF at least twice a week [12].

In a study undertaken in Kumba, Cameroon, mobile food vending was shown to be part of a survival strategy for the poor, who attempt to maintain and expand the subsistence business [13]. As such, it is imperative that local and regional policy-makers forge strategies directed at growing the economic well-being of poverty-stricken families and develop SF enterprises into city food establishments. Willemse [7] recommends that it will be valuable if policy makers, and researchers, address problems encountered/experienced by the poor who are trying to enter informal trading.

As important as SF vending is, this business increases the strain on local government as it is difficult to manage because of its informal nature, risk of microbial contamination and selling of unhealthy (high fat, salt and sugar, highly processed) foods [2, 14, 15]. For example, regulating what is sold on the streets becomes a challenge, as government officials are not always able to monitor the type of food sold (whether nutritious, hygienic or safe). In this regard, initiatives that foster healthy, safe products, and profit SF vendors are needed to ensure that SF is not sold at the expense of the consumer’s health. In Nairobi researchers found that vendors sell what consumers will buy, demonstrating purchasing power [16]. In SF vendor studies basic hygiene practices such as handwashing between handling food and money as well as toilet breaks, and the use of a hairnet when cooking has been observed in studies in Brazil and South Africa [17, 18]. Both studies also recognized a huge lack of infrastructure and basic services/poor environmental conditions [17, 18]. Ideally, one wishes to create a SF vending business model that would be economically sustainable, and offer healthy foods, which are microbiologically safe to clients. Such a model would provide the greatest benefit to vendors and their clients and would also reduce the monitoring burden on local government.

However, to our knowledge, no-one has developed a sustainable SF vending model (SFVM) in a metropolitan area in South Africa that encompasses good business practices with the sale of nutritious foods which are microbiologically safe to eat. Thus, the overall aim of this study was to develop a SFVM that considers nutrition, hygiene and safety, business and operational aspects of SF vending, including a vending cart. The overall goal of the Global Strategy on Diet, Physical Activity and Health, of the World Health Organization [19] ties in with this model. The strategy is, to “promote and protect health by guiding the development of an enabling environment for sustainable actions at individual, community, national and global levels that, when taken together, will lead to reduced disease and death rates related to unhealthy diet and physical inactivity” [19] [page 3].

## Methodology

### Overall aim

**Fig 1:**
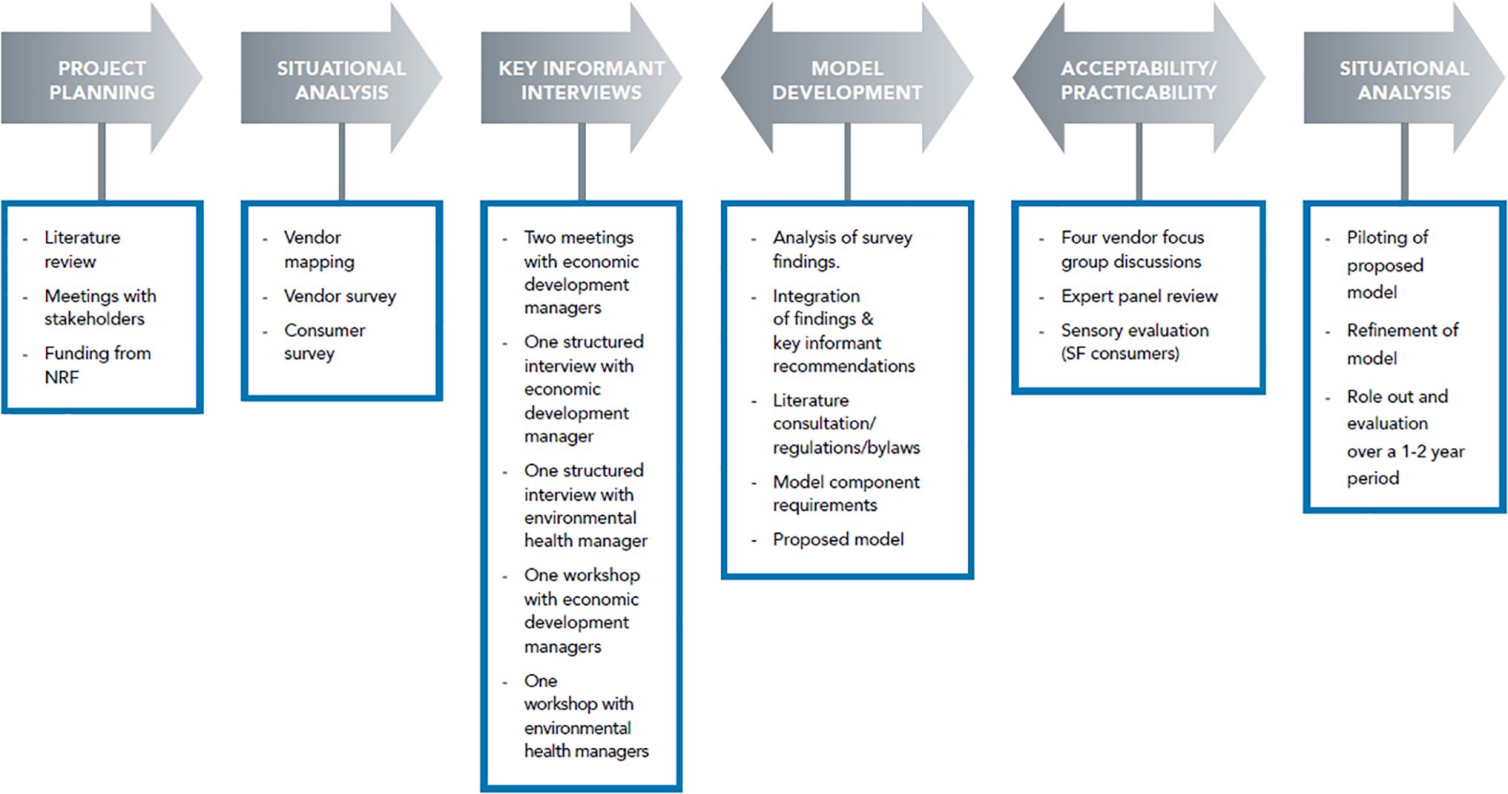
The process of the development of the street food vending model. *Previously published: Literature review; Vendor and consumer surveys [8, 9, 18, 20].

In this study, which was conducted in three phases, we made use of mixed (quantitative and qualitative) methodology. Phase 1 results have been published [9, 18, 20]. However, Phases 2 and 3 are unpublished and will form the bulk of this article. Fig 1 presents an overview of the study with all its components.

#### Phase 2a. Qualitative data collection from government officials

The main objectives of this phase were to identify the current regulations and policies governing SF vending and to gain insight into SF vending operations from a regulatory perspective. Phase 2a of this study comprised qualitative methodology utilizing in-depth interviews and focus group discussions with staff from the Western Cape Department of Environmental Health (DoEH) and Department of Economic Development (DoED). These two departments are recognized as the only two governmental/municipal departments directly involved in supporting and servicing SF vending. Individual interviews were conducted with one DoEH and one DoED manager. A semi-structured interview schedule was used as a guide to steer the interview. Questions asked, pertained specifically to regulations, bylaws and policies that relate to SF vending, certification, requirements regarding business and hygiene, and support available to vendors. Both key informants spoke freely and openly allowing for rich information. Two focus group discussions were held with the officers from the DoEH (N = 10) and DoED (N = 12).

Individual interviews and focus groups were transcribed, after which a summary of each interview and focus group was written. This process was followed for the interviews and focus group discussions with DoEH and DoED officers. The primary researcher familiarized herself with the data by reading each transcript several times and then used the Computer-Aided Qualitative Data Analysis Software (CAQDAS) package, Atlas ti 7.5.7, to manage the data. The four transcripts were loaded into Atlas ti 7.5.7 as four primary documents. Then the coding process commenced by reviewing the transcripts and allocating codes and giving them a concise label (open coding) [21]. After consultation with team members, the researcher then began reviewing all codes and merging and deleting ones where applicable, which was repeated a few times. The researcher then began grouping quotes under predetermined themes with summarizing sentences, thus, placing them into categories. Codes and their connected quotations were retrieved to explore patterns or tendencies.

#### Phase 2b. Document review

The objective of Phase 2b was to identify the existing regulations and policies on SF vending. Document review was employed as an additional method of data collection. Documents were accessed through official government websites, nationally, and the City of Cape Town was searched specifically. Documents from websites about street-vending business guidelines were also searched.

#### Phase 3

The objectives of this phase were to integrate the data obtained from the two earlier phases, assess the relevance, acceptability and feasibility of the identified themes and resulting components with SF vendors, and develop a SFVM. A socioecological framework was applied in this phase to understand, interpret, and apply results of the previous phases to inform this SFVM development.

Step 1: Integration of data: Data from the two surveys undertaken in Phase 1 and the outcomes from Phase 2 were studied, interpreted, and integrated to develop main themes and components which would contribute to the development of the SFVM within a socioecological framework (Table 1).

**Table 1:** Placing the street food model within the context of the socio-ecological model.

Component	Intrapersonal factors	Interpersonal factors	Physical Environment	Policy Environment
**Business**	Business & operational knowledge & awareness	Engaging with relevant government officials & fellow vendors		Regulation & bylaw awareness
		Attracting & maintaining clientele		Adhering to rules and regulations
**Food/Nutrition**	Food & nutrition knowledge & awareness	Attracting & maintaining clientele	Healthier foods for sale	Adhering to rules and regulations
**Hygiene**	Hygiene Knowledge & awareness	Attracting & maintaining clientele	Better hygiene practices	Adhering to rules and regulations
**Vending Cart**		Attracting & maintaining clientele	Basic facilities available	Adhering to rules and regulations

Step 2: Determining relevance and feasibility of data integration: In this step, we tested the relevance and feasibility of resulting themes and components with the use of participatory action research (PAR) using focus group discussions with SF vendors. Participatory action research recognizes the importance of involving those who are intended to be the beneficiaries of the research, with specific reference to the working class, laborers, those exploited, and the poor in a breakdown of their reality [21]. The aim of PAR is to address the practical concerns of people in a problematic situation as well as the larger goals of social science, with impetus on rigor and relevance [22]. A profound sense of ownership, which a PAR approach enables, would promote the sustainability of the proposed SFVM.

As the main focus of this part of the research study was to assess the proposed SFVM, focus groups were conducted with SF vendors to evaluate the acceptability and practicalities of the proposed model. Data collection took place from September 01 to November 17, 2015. Three focus group discussions took place in central Cape Town, and one took place in Mfuleni (a suburb in the Northern suburbs of Cape Town). Focus groups comprised five to nine participants, totalling to 28, with 20 females and eight males. Face-to-face focus groups were conducted and audio-recorded. A semi-structured focus group schedule was used as a guide to steer the discussion. Questions were specifically geared to assess various components identified in the previous step, i.e. business, food and nutrition, hygiene as well as a proposed SF vending cart.

The focus group discussions were audio-recorded, and the primary researcher listened to these a few times before coding commenced. A pre-determined coding list was used to categorize information into acceptability, practicability, perceived challenges and suggested changes to the proposed SFVM.

The primary researcher followed the same qualitative method as before by listening to each recording and then using the CAQDAS package, Atlas ti 7.5.7, to assist in managing the data. This software allows for basic ‘code retrieval’ of data as well as a more sophisticated analysis using algorithms [23]. The four recordings were loaded into Atlas ti 7.5.7 as four audio files. The researcher then commenced the coding process by listening to the audio files and allocating sound bites to pre-determined codes in consultation with supervisors and senior project consultants. The researcher then began grouping sound bites, transcribed relevant quotes under predetermined themes with summarizing sentences, thus, placing these into categories.

Step 3: Development of the street food vending model: A model can be interpreted as a descriptive strategy that has a broad conceptual framework [24]. Models are characterized using analogies to give a visual illustration, with the aim to simplify phenomenon as a tool for explanation and conceptualization [25].

Sallis *et al*., [26] indicated that ecological models of health behavior emphasize the environmental and policy context of behavior, though they do include social and psychological issues. In an ecological model, each level of influence is considered, thus, guiding the development of comprehensive interventions. The most relevant potential influences should be considered at each level. These assist researchers/intervention planners in appreciating how people interact with their various environments. In turn, this understanding can be used to develop effective multi-level methodologies to better health practices [26].

The socioecological model used in this study represents a comprehensive approach to designing, implementing, and evaluating interventions which target multiple influences on behavior [27]. The premise of an ecological model/perspective supposes that individual behavior should be considered as being affected by various levels of influence, including individual and environmental determinants [28]. The five levels of influence to health behavior, in particular, include intrapersonal factors, interpersonal processes, primary groups, institutional factors, and community factors as well as public policy [29].

### Ethics

This research received ethical approval from the University of the Western Cape’s Ethics Committee (Registration no: 14/4/17). Ethical approval for the larger study to intervene with SF vendors and consumers was previously obtained from the Human Sciences Research Council (Protocol No REC13/20/02/13). In addition, permission to intervene with SF vendors was obtained from the City of Cape Town (ID No. 10341).

All participants were informed that participation was voluntary, with the understanding that they could withdraw from the study at any time without any consequences. Before the interview/focus group, discussion participants who gave informed consent received an information sheet explaining the study in detail and what was expected from them. Anonymity was assured in that their names were not used in any report and the collected data were only accessible to the study investigators.

## Results

### Phase 2a: Key informant interviews and focus group discussions with government officials

Five broad categories were identified through the analysis process (Table 1): Regulations, business-related issues, food/nutrition/health, environmental health/hygiene, vendor cart, and vendor challenges within the SF operation.

#### Vendor operations

Participants were asked about what they see as the most important aspects of the SF vending operation. All extracted codes about the business operation of SF vendors were grouped and presented in Table 2.

**Table 2:** Integral elements of the business operations of vendors as identified by participants.

THEMATIC CODES	Environmental Health		Economic Development		Total
	Manager SSI	Officials FG	Manager SSI	Officials FG	
Business Act/license	4	5	0	5	14
Business plan/guidelines	1	3	0	2	6
Certificate of acceptability	2	4	0	4	10
Environmental health/hygiene	13	14	2	0	29
Food/nutrition/health	0	16	0	9	25
Informal trading plan	0	0	2	3	5
Legislation/regulations/bylaws	15	11	12	1	39
Permit/trading zones	5	5	0	15	25

SSI = semi-structured interviews; FG = focus groups

As can be observed in Table 2, the most attention given by participants was regarding the legislation, regulations and bylaws to which the SF vendors should adhere to, to run a legally compliant operation. Discussions on the hygiene or environmental health aspects of SF vending were next. Participants also mentioned food, nutrition and trading zones frequently, with food and nutrition-related aspects only mentioned in the focus group discussions and not in the interviews with the managers. The density of the codes presented in Table 2, shows that more robust discussions around the perceived integral elements of the business operations of street vendors took place with officials from the environmental health sector.

Regulations: The Foodstuffs, Cosmetics and Disinfectants Act 54 of 1972 (R962) [30], recently changed to R364, was the regulatory aspect to which interviewees most often referred to in the interviews and the focus group discussions. One key informant referred to this as, *“*R962 that is the main one that is the Bible.” Another key informant explained that “…the certificate of acceptability is a regulation and has been promulgated under the foodstuffs act.” Participants explained that vendors selling cooked food should be in possession of a certificate of acceptability (COA) requiring the vendor to comply with certain standards for health and safety. The DoEH issues the COA, but before being able to apply for the COA, the vendor would have to apply for a “hawker’s meal trading license” promulgated under the Business License Act of 1991 [31] at the cost of R10 (less than 1USD).

Participants also indicated that regarding the Informal Trading Bylaw of the City of Cape Town, no-one is allowed to trade informally on City property in a trading area without a valid permit. Therefore, a SF vendor potentially should be in possession of three documents to operate legally, namely, a COA, a hawker’s trade license and a permit.

Environmental health and hygiene: This aspect of SF vending was an obvious concern to officials from the DoEH. The importance of including health and hygiene aspects into a business model for vendors was discussed from various perspectives. One participant referred to the importance of temperature control as being key, and in recognizing the limitations that SF vendors experience, the DoEH deems the use of cooler boxes as cooling equipment to be acceptable, “…that is why we accept the cooler boxes. So, if your product goes out of your home in the morning at 4° Celsius, you have ice boxes and cooler bags and things like that. [Then] you can maintain it at a safe temperature unless you are going to leave that bin open or that container open, or you [are] going to open and close … it, and things like that. So that is why I say that [a] business model is very important.” [DoEH manager]

The participant also emphasized that vendors should possess the knowledge to conduct temperature control measures appropriately, “…and then, of course, keeping things hot that is meant to be hot. Don’t make the sausage 10 am this morning then it sits outside [at] room temperature, 28 degrees in the center of town and you are only warming it up again to put it on a roll or something like that.” [DoEH manager]

Participants from the DoEH indicated that SF vendors should at least receive a copy of the WHO’s, *Five Keys to Safer Foods [32]*, endorsed by the South African Department of Health, even if no training is possible. The DoEH manager noted, “The five safer foods [were] developed for Africa and for every region they sort of took certain things under consideration. So, this is really something that has been researched for South Africa. This particular one that we use.” “We use Five Keys, that is one of the things that is [needed by] everybody in the City, we adopt [the] World Organization[‘s] Five Keys that is what we preach”. [DoEH official]

Environmental health officials attested to regular inspections of premises and food sampling of SF vendors. “…because you need to guard against food poisoning, so that is one of the things that can be used as well.” [DoEH official]

Food, nutrition, and health: Food, nutrition, and health were also concerns of the key informants as can be seen from Table 2 and the following quotations. Most of the comments were participants’ concerns about the unhealthy nature of food items for sale by SF vendors.

“So they would sell anything from *koeksisters* [type of doughnut/oily cakes] to sweets to toffee apples to vetkoek [deep fried bread] …and many of those kids’ parents don’t give them food for lunch so they would get money.”

[DoED official on vendors operating around schools]

“Another sensitive area is your clinics… vendors are all around the fence, and they sell, because now the patients are waiting, they wait in long queues, so it’s chips, it’s cookies, it’s ‘Niknaks’ all of those things and that is carrying into the facility as well.”

[DoED official on vendors operating around clinics]

“A lot of the items that are sold by the SF vendors are unfortunately not very healthy items, and it is not going to change on its own.”

[DoED official]

One key informant felt that he is not sure on how prescriptive one should be regarding what vendors can and cannot sell. Because if the vendor does not make a profit, then the officials will be to blame. “I in my mind, haven’t drawn that line yet. I absolutely understand cigarettes and school children, but to me where do you start drawing the line, what about somebody selling hamburgers where you fry it in oil that is also not healthy.” [DoED manager] “So to me whatever you are selling it is there because the City have [sic] been prescriptive, then who takes the responsibility when that person’s model doesn’t work.” [DoED manager]

Although business plans are not required when applying for a business license, COA or location permit, they should be encouraged to develop such plans, and there is some support available for this, a key informant said. “We have a unit within DoED called *business support*, and we refer people to do it through that, and then throughout the municipal area, there are organizations which the City funds. We can provide that, and that helps you develop your business plan, your business case, looks at various financial options, so those models are available.” [DoED manager]

“The simplest of logs and the simplest of business plans, where do I buy my things. What do I do to keep it good? What remains of the stock that is left over tonight? Because meat and things like that does [sic] not go off, you can use it, keep it refrigerated and frozen. There’s nothing wrong with using it the next day. But don’t defrost it [and then] freeze it again. And the volumes that they need to get [a profit] and that is where people sometimes get into trouble. And the thing that you throw away is money. The moment that you throw away it is waste, it is absolute money.”

[DoEH manager]

Challenges regarding the street food vending operation: Codes regarding challenges in the vending operation are presented in Table 3. The salient issues regarding challenges in the vendor operation were recognised as a lack of consumer and vendor knowledge regarding health and hygiene. This was directed towards the vendor practices and the consumer not ‘asking’ and thus not knowing any better. “My thinking, and it is a long way to get there, is surely what we should be encouraging. That [is] if a trader wants to then sell healthy food stuff we should be encouraging them. But, we shouldn’t say you must sell healthy food but say if you train them in education these are the better alternatives, these are the better options, and we would encourage [this]. And then if people like this lady who sells fruit and vegetables that is fine, and [if] there is another trader who is selling sushi that is fine, but again what do we do when the mindset of the trader is saying, I will sell what the commuters want. Now, the commuters are not necessary talking about healthy food; they want hamburgers.” [DoED manager] “To me, the approach would be two-fold, one is to educate the trader absolutely, but one needs to also [sic] educate the community.” [DoED manager]

**Table 3:** Codes regarding the challenges affecting the street food vending operation.

CODES	Environmental Health		Economic Development		Total
	Manager SSI	Officials FG	Manager SSI	Officials FG	
Consumer education	0	13	1	0	14
Facilities built not used	0	0	3	0	3
Financial assistance	0	0	2	0	2
Healthy = expensive	0	0	7	0	7
Mobile vending/seasonal	1	3	1	0	5
Security of tender	0	0	1	0	1
Storage	1	1	1	3	6
Vendor associations	0	0	0	1	1
Vendor challenges	5	1	0	0	6
Vendor education/training	2	5	6	0	13
Waste management	0	4	0	0	4

SSI = semi-structured interviews; FG = focus groups

The issue of healthy foods being expensive came up in one manager’s interview several times, but not in any of the other discussions or interviews, while the challenge of vendors acquiring storage space came up in all the discussions. “The question of storage is [a] personal/private storage arrangements. Most traders have engaged businesses for their private storing purposes. So, they have to get out by a certain time, as obviously businesses must trade, so by 6/7 am. And then they must be in at a certain time as the business closes for the day. Even though there might still be ‘traffic’ in Wynberg [a suburb], they then lose out on that business.” [DoED official] “Irrespective of the design, the issue in my area is storage. I have a situation now where a trader stores her goods in a shop.” [DoED official]

Other challenges were also acknowledged, including crime and the lack of basic facilities such as water and energy sources at many stalls. “When people are complaining about crime and your safety issues … I can see their frustration and things. It would be difficult to operate where you do not have electricity, and so on, but then there are people who are running successful little businesses. That has actually gone in from tiny, tiny starts to bigger things. And have become good caterers, so it is a constraint, but it does not prevent people from trading.” [DoEH manager]

The issue of street vending being difficult to manage because of its mobility and seasonality came up in three of the four discussions. “*Ja* look, I think that is the reality, unfortunately. Because some of the constraints that we have that colleagues find, is the fact that you have a very seasonal thing, and you have a very occasional sense. End-of-month things, end-of-week things, lots of people actually operate on a Friday, Saturday, Sunday. And they spring up; some may operate for four, five, six weeks or two months and then all of a sudden, they are no longer there, or whatever the case may be.” [DoEH manager]

Waste management also appears to be a key issue when considering the vendors’ operations.

“It’s a big issue for us, especially what do you do with waste? So you’ve got to try and build in some… waste minimization strategy because people don’t like to take the waste home.”

[DoEH official]

Cart requirements/suggestions: When key-informants were asked to raise an opinion regarding the use of a cart/mobile unit for SF vendors, a few themes emerged which is noted in Table 4. Emphasis was placed on the fact that any cart designed for vendors’ use should comply with health and hygiene regulations as stipulated by Act R962, recently replaced by R364, the Foodstuffs, Cosmetics and Disinfectants Act, 1972 [30].

**Table 4:** Street food vending cart considerations.

CODES	Environmental Health		Economic Development		Total
	Manager SSI	Officials FG	Manager SSI	Officials FG	
Cart requirements	1	9	1	4	15
Cart challenges	0	0	1	4	5
Cart example	1	8	2	8	19

SSI = semi-structured interviews; FG = focus groups

One challenge identified about the cart was that of finances as it may be too costly for the average street vendor to purchase. Lack of storage space for such a vehicle was another challenge. The key informants could provide some examples of existing carts and initiatives for designing an “ideal cart” which researchers could explore.

“I think the main thing is that gas and stuff like a cooling facility, the most important ones are usually your gas or whatever your preparation things [are] that you use and then your storage facilities. Your cool bags or cool facility, and something to wash hands. I think that is the main aspect of the preparation thing, that cold-chain stuff, the cooling system and your cleaning system.” [DoEH official] “You see what the people usually do, they put in something like a little washing basin thing like they do in a caravan with a 25-L or 20-L plastic can, draining it down at the bottom where your waste water is going, which you can empty tonight, because that is what they usually do.”

[DoEH official]

“Maybe what I could say regarding the cart, it is a very good idea but just looking at the financing. Have you looked at [whether] the people can afford this?” [DoED official] “…the units are fantastic on one side because if you’ve got a healthy-looking unit, it attracts people to buy and be selling healthy food even better. But there are [an] enormous amount of challenges around the units, and the key of that is to forget the finance, it costs money to build a unit but the storage and the ability so when operating at BTI [Bellville transport interchange] where do you store it?”

[DoED manager]

“I think the cart of the Cape Winelands (an area using a cart) will bring with it some answers to what you are looking for. They had a safe component in it. There is, I cannot remember if it is a tap or 25-L water container–clean water thing in which one can use the sink maybe to wash maybe your vegetables or whatever meat or anything and then at the bottom, there is also an empty 25-L container to drain (the water). So it’s a typical–an ideal model that really satisfies us DoEH practitioners because it has all the components we are looking for. And I think, also has a space for them to label their stalls because our regulations R962 requires that if it is a stall that you are selling from then it must be clearly labelled with your name and everything.”

[DoED official]

Hygiene standards: About hygiene training provided to the SF vendors in Cape Town, the *Five Keys to Safer Food* developed by the WHO, 2006 [32] appears to be the main document in use by the DoEH officials. The officials from the DoEH discussed issues around the *Five Keys to Safer Food* as noted in Table 5. The five keys to safety cover the following aspects: keep everything clean; separate raw and cooked food; cook thoroughly; keep food at safe temperatures, and use safe water and raw materials. “Training in this regard, we don’t really [give training], but we try and get (reach) the hawkers (vendors) through the Five Keys to Safer Food initiative. I know in Khayelitsha (suburb) they have a nice thing going with the hawkers, they actually get them in from time to time, and train them. But it is not a pre-requisite for us.” [DoEH official]

**Table 5:** Five Keys to Safer Food codes.

CODES	Environmental Health		Economic Development		Total
	Manager SSI	Officials FG	Manager SSI	Officials FG	
Five Keys to Safer Food	4	3	0	0	7
Five Keys to Safer Food training	0	1	0	0	1

SSI = semi-structured interviews; FG = focus groups

### Phase 2b Document review

In Table 6, the 13 regulations and bylaws that directly or indirectly affect SF vendors in the Western Cape Province are shown. Most of these regulations are South African laws, while a smaller number are City of Cape Town bylaws, and as such effect only those vendors trading within Cape Town.

**Table 6:** Regulations and bylaws deemed applicable for street vending.

Regulations	Key aspect	Source
**National**		
The Foodstuffs, Cosmetics and Disinfectants Act 54 of 1972 (R962)	To control the sale and manufacturing of foodstuffs	https://www.capetown.gov.za/en/CityHealth/Documentation/Documents/Reg_Regulations_Gov_Gen_hygiene_Food_Premises.pdf
The Health Act 63 of 1977	Nutritional intervention/environmental conditions that constitute a health hazard	https://www.capetown.gov.za/en/CityHealth/Documents/Legislation/Act%20-%20National%20Health%20Act%20-%2061%20of%202003.pdf
The International Health Regulations Act 28 of 1974	To apply international health regulations	http://www.gov.za/sites/www.gov.za/files/Act%2028%20of%201974.pdf
The Liquor Products Act 60 of 1989	To provide for control over the sale and production for sale of certain alcoholic products	https://www.westerncape.gov.za/text/2003/liquor_products_act_no60_1989.pdf
The Abattoir Hygiene Act 121 of 1992	Maintenance of proper standards of hygiene in slaughtering of animals for human consumption	http://www.enviroleg.co.za/acts/Abattoir%20Hygiene/Abattoir%20Hygiene%20Act.pdf
The Agricultural Product Standards Act 119 of 1990	To provide control over the sale of agricultural products	http://www.nda.agric.za/doaDev/sideMenu/foodSafety/doc/Act%20119.pdf
The Animal Diseases Act 35 of 1984	To provide control of animal diseases and parasites	
The Business license act 71 of 1991	Laws regarding the licensing and carrying on of businesses/Hawkers license	https://www.capetown.gov.za/en/CityHealth/Documents/Legislation/Act%20-%20Businesses%20Act%20-%2071%20of%201991.pdf
**Local government**		
City of Cape Town informal trading by-law 2009	City is responsible for “trading bylaws”/”markets”/”street trading”/”beaches”	https://www.capetown.gov.za/en/ByLaws/Promulgated%20bylaws/Informal%20Trading%20By-law.pdf
	To promote social and economic development	
City of Cape Town: Integrated Waste Management Amendment By-law 2010	Waste management Littering/dumping/spillage	http://www.capetown.gov.za/en/ByLaws/Draft%20bylaws/Integrated_Waste_Management_Bylaw_13-JAN-2010_final_draft_Eng.pdf
City of Cape Town: By-law relating to community fire safety 2002	Fire hazards/fire safety equipment	https://www.capetown.gov.za/en/ByLaws/Promulgated%20bylaws/Community%20Fire%20Safety%20By-law.pdf
City of Cape Town: Traffic bylaw 2011	To control nuisances emanating from the operation of public transportation (vendors operating at transport interchanges, possible nuisance)	https://www.capetown.gov.za/en/ByLaws/Promulgated%20bylaws/Traffic%20%20By-law%202011.pdf
**Regulatory Guidelines**		
City of Cape Town: Guidelines for conducting a food vending business (hawking in meals 2000)	Stipulates requirements for running a street food vending business	http://web.capetown.gov.za/eDocuments/General_-_Operating_an_Informal_Food_Vending_Business_-_Guidelines_105200503949_245.pdf

### Phase 3

#### Step 7 Data integration

The data from the survey on vendors and consumers (Phase 1) and key informants and government officials (Phase 2) were integrated (Table 7).

**Table 7:** Key themes from surveys and key informant interviews and focus groups.

Theme	Survey data [9, 18]	Key informant data
	Business/operational issues	
Regulations, bylaws [Licensing & permits]	63% of vendors had no form of licensing.	Business license “hawker’s meal trading license promulgated under the Business License Act (1991) at a cost of R10.” Certificate of acceptability “R962, that is the main one, that is the Bible.” “…the certificate of acceptability is a regulation and has been promulgated under the food stuffs act.” Permit “40% of the municipal areas you don’t need a location permit.”
Business operations	Long hours/days (87% vendors worked six days a week). Little profit (67% made less R1 000 a week). 28% did not keep an inventory. Stock purchases: 29% of vendors bought wholesale and 21% bought straight from fruit and vegetable markets. 70% of vendors stored their stock at home. 60% of vendors store cash in their pocket. 22% of vendors called for financial assistance. 9% of vendors would appreciate business guidelines.	Business guidelines “The simplest of logs and the simplest of business plans, where do I buy my things and things like that. What do I do to keep it good? What remains of the stock that is left over tonight?” “We have a unit within economic development called business support and we refer people to do it through that and then throughout the municipal area there are organizations which the city funds we can provide that and that helps you develop your business plan, your business case, looks at various financial options so those models are available.” “Irrespective of the design, the issue in my area is storage. I have a situation now where a trader stores her goods in a shop…” Vendor training “I think I mentioned it right at the beginning your consumer education you’ve got to really look at because as the one thing is the vendor but also the vendor will turn if there is no profit.”
**Food and nutrition**		
Unhealthy food items for sale	46% sold packaged snacks (potato chips, chocolates, etc.). 21% sold sweetened beverages.	*Food*, *nutrition and health* “A lot of the items that are sold by the street food vendors are unfortunately not very healthy items and it is not going to change on its own.” “So, they would sell anything from *koeksisters* [doughnuts] to sweets to toffee apples to vetkoek [deep fried bread] to what…and for many of those kids’ parents don’t give them they don’t put in food for lunch so they would get money.” Vendor training “To me the approach would be two-fold one is to educate the trader absolutely but one needs to also educate the community…”
Cooked food	28% of vendors sold cooked food. 72% of consumers purchased cooked food.	
Poor nutrition knowledge	15% of vendors had an acceptable nutrition knowledge. 12% of consumers had an acceptable nutrition knowledge.	
Intention to purchase healthier foods	96% of consumers indicated a willingness to purchase healthier food items should these be available.	
**Hygiene and safety**		
Hygiene practices	80% of vendors handled money and food without washing their hands in-between. 87% of vendors selling cooked food had a poor cooked food handling score.	Hygiene standards [Five Keys to Safer Foods] “We use [the] Five Keys [of safety] that is one of the things, that is everybody in the city we adopt [the] world organization (WHO) Five Keys [of safety] that is what we preach.” “Actually, interesting to note; the five safer food was developed for Africa and the …-, for every region they sort of took certain things under consideration. So, this is really something that has been researched for South Africa. This particular one that we use.” Vendor training “Training in this regard we don’t really but we try and get the hawkers through the five keys to safer food initiative. I know in Khayelitsha they have a nice thing going with the hawkers, they actually get them in from time to time, and actually train them and things like that. But it is not actually a pre-requisite for us.”
**Vending cart**(to attempt bridging the gap between the lack of facilities)		
Lack of basic facilities	69% of vendors mentioned the lack of basic facilities such as water and electricity. 16% had access to electricity or gas. 11% had access to a stove. 8% had access to cold storage.	Cart requirements “Irrespective of the design, the issue in my area is storage. I have a situation now where a trader stores her goods in a shop…” “I think the main thing is that gas thing and stuff like a cooling facility, the most important ones are usually your gas or whatever your preparation things that you use and then your storage facilities. Your cool bags or cool facility, and something to wash hands. I think that is the main aspects of the preparation thing, that cold chain stuff that cooling system and your cleaning system.”

#### Step 2: Relevance, acceptability, and feasibility of the integrated themes

All SF vendor participants in the four focus group discussions agreed to the relevance, acceptability, and feasibility of including the components business and operational issues, food and nutrition, hygiene and safety, and cart with basic facilities, in a SFVM. In the previous step of data integration, this model was identified (Table 7). In the following section, we present and discuss the perceptions of vendors on the components of the four main themes, as reflected during the group discussions.

Business and operational issues: In Table 8 some of the reflections and ideas that vendors shared concerning the business-related themes and components proposed for the SFVM are presented. The inclusion of licensing and granting of a COA seems to be important to include in a business and operational component of the SFVM. The reason being that most participants were not in possession of any form of licensing and were keen to know where to go, what process is involved, and how long this would take.

**Table 8:** Vendors’ perceptions of the proposed business and operation’s component of a street food vending model.

Theme	Component	Supporting quote
Business/ operational issues	Regulations, bylaws [Licensing & permits]	“I believe the department did come and ask for this food license, but the time they give us the shop, they never told us you have to have before you sell. I don’t know how we are going to get those certificates now”. They actually came to us and asked for food license. When you give someone a kiosk and a key you supposed to give food license. But they never did that, so what are they taking us for? They think that we are stupid, we are not stupid”.
	Business plan/guidelines (marketing)	“Basically, when I started out I didn’t do thorough research of what needed to be done…I don’t really know the ins and out”. “Just do thorough research don’t just go in before you know what the challenges is and when does the business pick up. Like she said some days are better”. “Make it nice, can you do it yourself? A menu maybe, or an umbrella, everything must just look attractive”. “I give then a smile when they come to my place. I keep my place clean and become talkative so that they can come familiar”.
	Business operations (stock/inventory and storage)	“We do not have money to stock up a lot of stuff. And we do not even have space”. “Cos I can’t buy more stuff. Maybe I buy a hamper of vegetables and business is not good then the potatoes rot. And that is a loss. So, it is better to buy everyday”. “If we do have the money then we buy in bulk, because it is better for all of us. But that is only if there is money”. “You know what I like, I did not think of what you tell us, to take our money together, you know” “Log books…yes…books”. “Today business is good, tomorrow it’s bad, so how do you count R120?”
	Financial/and other support available to vendors	“Must you be registered?” “Do you have to pay back the financial support?” “So, is there some financial support? If I want to start a business and have no money?” “But I think a lot of us do need training when it comes to consisting of how to go about your business. Even your money, how to because some of us make money, tomorrow you must give it out again. You must buy the same stuff, and so it goes on and so it goes on …”

Vendors commented on the fact that inspectors come to their premises asking for a food license, but do not always explain the process of applying for a license. Similarly, in Cameroon, the lack of certification of SF vendors, suggests that amid the fast-growing SF sector, it is of great importance for the urban councils to make the SF vendors an essential part of local plans. As Acho-Chi [13] [page137] mentioned, this is “to encourage a competitive modern urban delivery system”. In such a system, licensing would be an essential component.

The group discussions showed that vendors regarded business guidelines as important and they liked the idea of doing research before starting a business. Of the vendors who participated in the discussions, 57% started their business because they were unemployed and needed a means of income. Therefore, they did not give much consideration to the facts and challenges involved in SF vending; they simply seized the opportunity. Though, in retrospect, they recognized that research beforehand might have been very helpful, particularly regarding location, safety, and foot traffic.

Another item of great interest to vendors was the financial considerations. One vendor strongly felt that one should save money before starting a business, “If I can just try to save because there is nowhere to go for financial support.” They were very keen to know whether there was any financial support available to vendors and what the process involved. Regarding buying food items wholesale, there were aspects that the proposed model did not consider. For example, vendors do not have the money to purchase large amounts of stock, and the perishable stock was a risk since the vendor could lose much through spoilage if the business were slow. Vendors were, however, very open to the idea of starting a vendor-purchasing club, i.e. buying wholesale as a group, and saving in this manner.

Regarding attracting clientele, most vendors rely on rapport with their clients. They indicated that it was important to make the stall look attractive and to be friendly to one’s clients. Also, regarding the general look and feel of the business operation, being clean, welcoming, and attractive. The participating vendors, although some kept logbooks, did not understand the necessity for keeping records. The comments and reflections in Table 7 attest to the importance of a business component in a model that wishes to facilitate SF vending operations. This component should consider guidelines regarding laws and regulation, basic market research, financial implications and available support, and other operational issues such as purchasing of stock and equipment, storage, and record-keeping.

Food and nutrition: The discussion on the nutrition theme invited some scepticism from vendors as is reflected by some quotations in Table 9 regarding selling healthy foods.

**Table 9:** Vendors’ perceptions of the proposed food and nutrition component of a street food vending model.

Theme	Component	Supporting quote
Food and nutrition	Unhealthy foods (Fat, salt)	“But then you get your customer also that like their fatty food and say give me that extra fat or whatever.” “We have all kinds of vegetables like your salad, your cucumber and your lettuce, we put all these things.” “I go through mayonnaise like it is hot chips because people love mayonnaise.” “I don’t think people will like it, I am sorry to say but we have been in this food, we know what people like already, they do not like healthy foods, do not even talk to them about healthy foods.” “At least my customers they are very health conscious so, they’ll always tell me don’t put in salt or…”
	Cooked food	“Yes, it is possible to cater for different kind of people.” “So maybe starting to use this pamphlet [booklet] you gave us today, there will be a change. With your support as well. Coming to visit us and checking, what we have promised you.” “If we can have something we could use for instance to cook, for instance to cook with less salt.”
	Poor nutrition knowledge (portion size)	“Ja we also think about healthy, but our customers they normally see the size, how big is your plate.” “If you try to put less food, you get a fight.” “You might cook healthy food, but it might be small for them.”
	Intention to purchase healthier foods	“Come to think of it a lot of people know this, you get a lot of, at least my customers they are very health conscious so, they’ll always tell me don’t put in salt or…” “Because there are some of the people there, yes, they say they don’t want fat, they don’t want this. And healthy food is good.”

Vendors believed that many SF consumers would prefer unhealthy foods. However, some vendors indicated that some of their clients do not want fatty and/or salty foods (Table 9) reflecting two types of consumers. Those who are conscious of what they consume and those who are either not conscious thereof or do not consider this important. Some vendors appeared to be open to trying healthier ways of preparing foods if they were provided with information and support in this regard.

Portion size was another obstacle that was identified, as consumers want value for their money. Vendors want to retain their clientele, so they serve large portions. Nutrition education is likely to be a bridging strategy in this regard to improve nutrition knowledge of vendors and consumers and to create an enabling environment and make healthier choices possible.

Hygiene and safety: A few comments regarding the hygiene components are reflected in Table 10.

**Table 10:** Vendors’ perceptions of the proposed hygiene and safety component of a street food vending model.

Theme	Component	Supporting quote
**Hygiene and safety**	Hygiene practices (Five keys of safety)	“I worked at Pick and Pay before I joined my mother, it is called the golden book, so that is why I know about it.” “CPUT. It was a workshop.” [was not a vendor then] “There in my work place. In the container, I am renting”
	Training	“No training, I thought today this was the training.” “They brought a drum and showed how to wash hands and clean.” “But we don’t even have anything, we don’t even have…” “No, she does not give us anything.”

Most vendors were not familiar with the *Five Keys to Safer Foods*, which were identified as being an integral part of environmental health by the DoEH officials. Only three vendors had seen these. One at her previous workplace, one had the guidelines in the stall she was renting, and the third vendor learnt about these at a workshop presented by the Cape Peninsula University of Technology. Only two vendors recalled receiving training with regards to hygiene. The reflections in Table 10 attest to the fact that there is a gap in hygiene knowledge and training, which would be key to improving hygiene practices.

Basic facilities: A well-equipped vending cart is proposed as an essential aspect of SF vending if no basic facilities are available (Table 11). Such a recommendation was also made following a study in Kumba, Cameroon, suggesting the introduction of suitable modern preservation technologies. For example, affordable refrigeration and heating units which would enable the storage and reheating of leftover foods, since food losses were found to be a common occurrence, owing to low demand and/or poor quality. Furthermore, with reliable heating and cooling facilities, SF vendors would be able to improve their income [13].

**Table 11:** Vendors’ perceptions of the basic facilities component of a street food vending model.

Theme	Component	Supporting quote
Basic facilities	Vending cart (as a bridge/gap between the lack of facilities)	“I don’t think it will work for me. I think this will work for someone that is just starting.” “It can work. It can make a difference.” “When we can see it practically. At least if we can see it we can comment (willingness to pay or it).” “I think it can help us. Sometimes we have football at the back, I can go there to make special food.” “Depends on the payment terms. It would be a good idea to buy, but we would have to know the payment terms.” A problem I see most people especially those starting a business, we always have is that of transportation, so if you have to take that now with, it is going to be a problem as well. And more especially the business, who is going to see to it, and you are going to have to pay that person.” “To keep it in storage.” “I see you have fresh water and refuse, the problem with those things where it lies now, they look at you, while you serving food you have to clean up some other stuff, then you have to throw it somewhere else. But it depends now, winter time you can survive, but in summer time you will find it is quite a lot of flies now. And the things are here and there are no drainages as well. This will keep it maybe for 7, maybe 6 hours. So, that might just be the challenges you come in and that may also effect the business on its own. Lots of flies, stuff like that and … They will ask you quite a lot of things because of the drainages and stuff like that. So it will be easier, but maybe not easier, because where are you going to dump your stuff, then this standing water that you use to clean, where at the end of the day will you dump it.”

Vendors indicated that the cart would be an asset for someone starting up a SF business, and not necessarily for someone who is already operating with a container or stall which is provided by the local authorities. However, when it came to financing the cart, they were very wary. They first wanted to see the final product and be informed about the terms of payment. Vendors also had some concerns around the vending cart regarding the storage, its transportation and the refuse container on the cart attracting flies. This proposed component would require further exploration and piloting in the field before additional recommendations can be made.

#### Step 3: Developing the street food vending model

**Fig 2:**
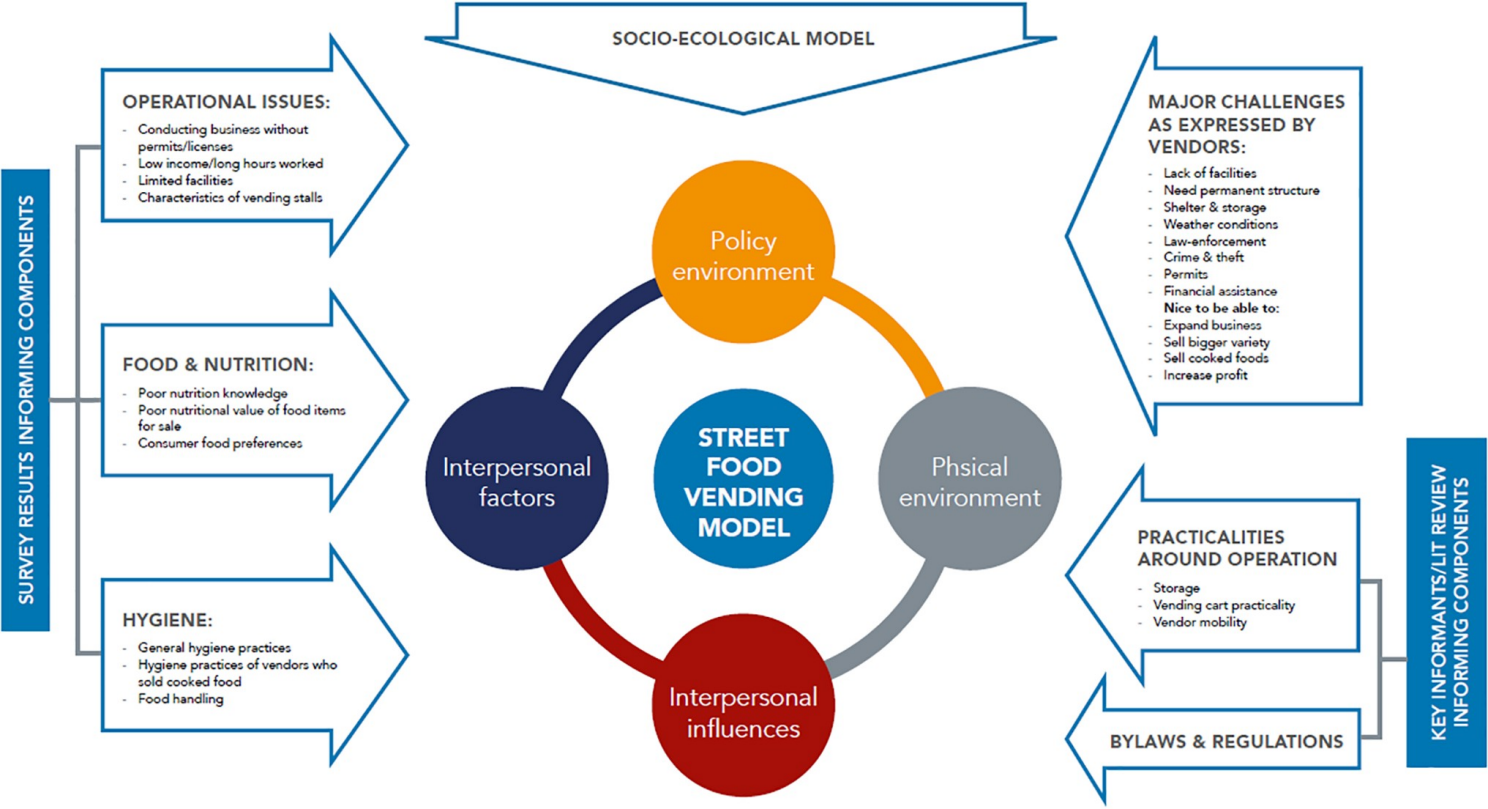
How the data sources influenced the development of the street food model.

In Fig 2, the process followed in developing the SFVM within the socioecological theoretical framework is depicted.

**Fig 3:**
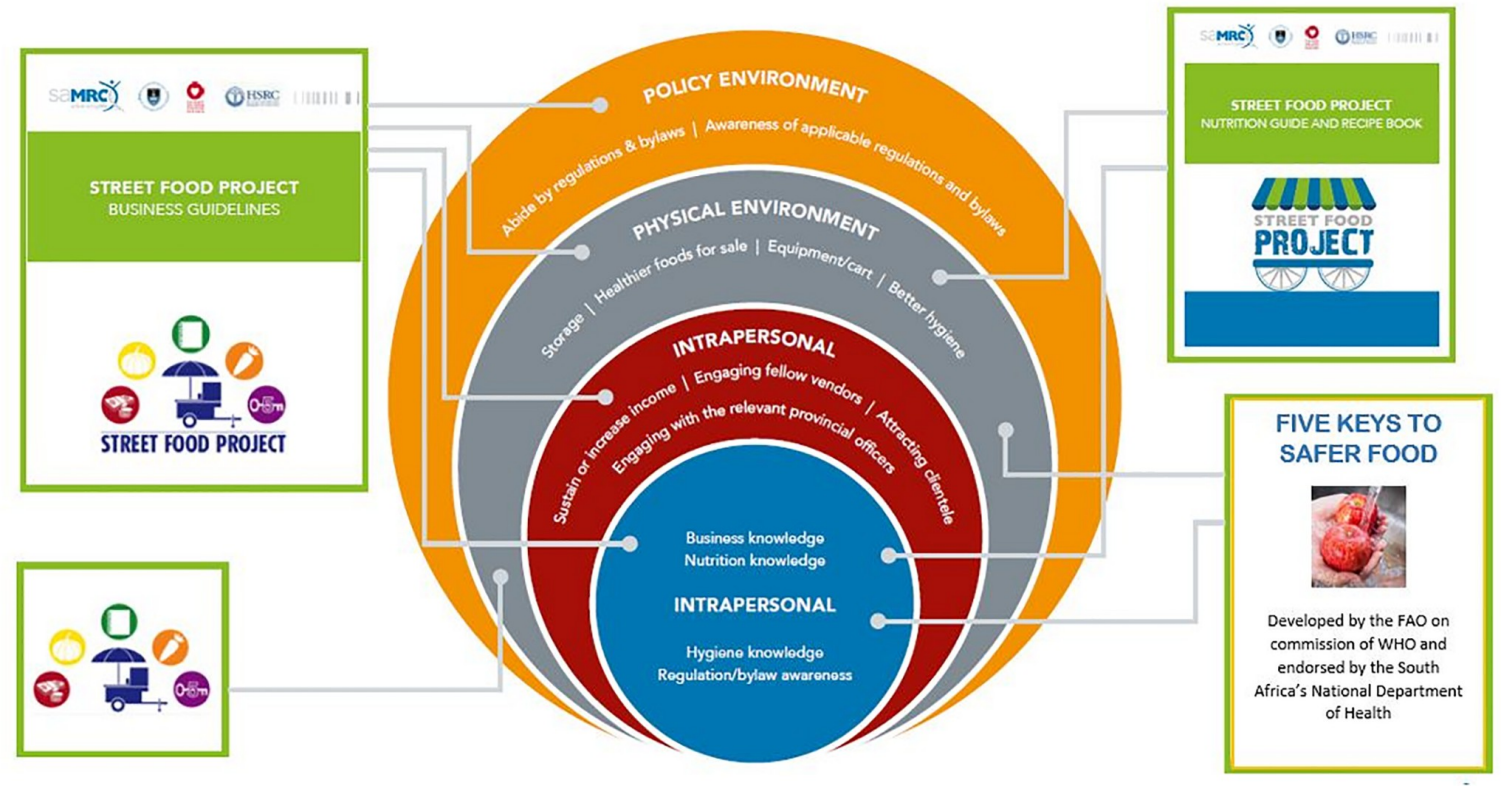
The proposed street food model with its tools *[tools available upon request from corresponding author]*.

The street food vending model components: From the findings of the previous phases and the integration of data in Table 1, the components of the proposed model can be divided into four areas, i.e. a business component, a food and nutrition component, a hygiene component, and a vending cart (Fig 3). These four areas, in turn, influence various areas of the socioecological framework, namely, intrapersonal/individual and interpersonal factors, the physical environment/community as well as the policy environment.

Sustaining any public health effort requires not only addressing the individual and their practices but also the context in which they live [33]. The guidelines provided here have been made as generic as possible to be useful to those in low- and middle-income countries and are visually depicted in Fig 3.

Business guidelines: The findings of the previous phases necessitate basic operational guidelines that will ensure good (legal) practice. The data showed that most vendors practised without any form of licensing or certification. Research from earlier phases showed that many vendors worked long hours, up to seven days a week, but still made less than R1 000 (71 USD) per week. Few vendors made use of a log book or inventory. Instead of buying at wholesalers in bulk, vendors made use of supermarkets or similar shops hence reducing their profits. Many carried their money in their pocket instead of keeping it in a safe place.

Thus, it is proposed that the business operation guidelines consisting of 12 concise messages be introduced to potential vendors looking to start a food vending business as well as to existing vendors. The 12 messages are, to do research; start-up finance; food type/s to sell; contact the DoED re: laws applying to street vendors; obtaining a business license/certificate; the vending cart/unit/stand; storage area for inventory and cart; purchasing wholesale; attracting clientele; promoting the business; keeping log books; safeguarding money.

Food and nutrition guidelines: Findings from the surveys of SF vendors and consumers indicated that most vendors sell packaged snacks, sweets, and sweetened beverages [9, 20]. Many consumers also purchased cooked foods [9]. The survey results also showed that vendors and consumers had poor nutrition knowledge. Basic food and nutrition guidelines can empower the vendors, providing knowledge, and thus enabling them to make better decisions concerning the food they sell and foods available at their homes. The South African food-based based dietary guidelines (FBDG) are recommended as a reference source for SF vendors (Table 12). These FBDG have been specifically designed. They are “short, positive, science-based messages that aim to change the eating behaviour of the general population towards more optimal diets that meet energy and nutrient requirements, while simultaneously helping to protect against the development of non-communicable diseases” [34].

**Table 12:** Revised general food-based dietary guidelines for South Africans.

• Enjoy a variety of foods
• Be active!
• Make starchy foods part of most meals
• Eat plenty of vegetables and fruit every day
• Eat dry beans, split peas, lentils and soya regularly
• Have milk, *maas* or yoghurt every day
• Fish, chicken, lean meat or eggs can be eaten daily
• Drink lots of clean, safe water
• Use fats sparingly. Choose vegetable oils, rather than hard fats
• Use sugar and foods and drinks high in sugar sparingly
• Use salt and food high in salt sparingly

(Vorster *et al*., 2013)

The hygiene component: The findings of the SF vendor survey indicated that hygiene and food safety were not optimal [9, 20]. Interviews with key informants from the DoEH indicated that this Department makes use of the *Five Keys to Safer Food* [32] to ensure food safety. During the document review phase, guidelines for conducting a food-vending business (*Hawking in meals*, 9 [31]) were obtained, which can be used in conjunction with the *Five Keys to Safer Food* [32].

The proposed street food vending cart: The vendor survey highlighted the lack of facilities available to SF vendors as did key informants. So, a mobile cart which adheres to basic requirements, as stipulated by the key informants (especially regarding size and applicable bylaws), as well as the guidelines for conducting a food vending business, should inform the design of a SF vending cart.

The resources outlined, i.e. business guidelines, nutrition guidelines, hygiene guidelines and a proposed cart can either be offered to the vendors as a package/toolkit, with or without training. Both existing and new vendors could access the entire toolkit or parts thereof as required.

## Discussion

Literature indicate that numerous uneducated, those with a low-level education as well as unemployed individuals, often women, find street vending a plausible way to earn money, with little monetary investment necessary [3, 8]. In phase one of this study the vendor survey concluded that SF vendors in the Cape Town and surrounding areas work long hours, up to seven days a week, and only make a minimal income. The types of food items sold by vendors, their nutrition knowledge and their hygiene practices were not ideal. Findings also highlighted the vendors’ struggle because of the lack of basic facilities and services, so essentially operating in a non-conducive (disabling) environment. An environment that enables unhygienic practices. Similarly, findings from Ghana conveyed that the settings where food vending is commonly practiced are scarcely resourced with low environmental and sanitary standards posing a major threat to food safety [35]. Bhowmik, (2005) [36] reported that vendors generally do not make much profit and as a result they tend to be mobile in the hope of finding better markets and increasing profits.

Concerning the nutritional value of food, the cooked foods sold as reflected in the literature are culturally-based dishes and not considered that unhealthy [35, 37–41]. However, the reviewed literature did not refer to packaged snacks, such as chips/crisps and sweets, which were popular items sold by the vendors who partook in the phase one survey.

The main findings of the consumer survey (phase one) indicated that people consume SF frequently (2–3 times per week). They spend a significant amount of their income on SF, and they are open to buying healthier options should these be available for purchasing. Thus, one could assume that should healthy SF be available [at a reasonable price], SF consumers would access these. Consumers’ nutrition knowledge, however, was not ideal. These findings resound findings from previous studies which reported that in developing countries, households which fall into the lower-income category spend up to 50–70% of household income on SF [11]. Chakravarty and Canet, (1996) [42] suggested SF as possibly the most affordable method of finding a nutritionally well-balanced meal option outside of the home environment. However, they added that the consumer should be educated and capable of choosing a healthy meal.

In phase two, key insights from government officials from the Departments of Environmental Health and Economic Development were that SF-vending business should be guided by the national legislature as well as by provincial bylaws. Government officials also strongly recognised the need for vendor and consumer education. For vendors, nutrition and hygiene as well as awareness of regulations for SF vending were emphasised, while health, food and nutrition education were considered important for consumers specifically. In a study conducted in Calcutta, vendors shared the desire to adhere to regulations, even though they were unaware of stipulated food regulations and had no training in food and nutrition. The vendors appreciated the fact that in order for the SF sector to be acknowledged and licensed they would have to comply with the specified regulations and guidelines as well as be open to inspection and food sampling [42], as is the case in this study. A study in Nairobi also recognised a lack of training in food preparation and hygiene practices and thus recommended the institution of SF centres with adequate facilities, training of SF vendors on hygiene as well as setting up a code of conduct for the SF trade [43].

The three phases and three distinct participant categories in this study allowed the research objectives to be explored from various perspectives. Themes from qualitative data echoed the findings of the survey data, such as the aspects pertaining to the business operation of the SF vendors, challenges faced by vendors as well as nutrition and hygiene. Discrepancies were also recognised in the data between key informants and vendors. Key informants felt that certification and licensing are relatively simple, and that support is available to vendors. However, survey results showed most vendors were unlicensed and without any form of certification, and it was noted in their challenges as something they find difficult and with which they would need assistance.

The data from the first two phases and step one and two of phase three were integrated within a socio-ecological framework to develop the proposed SFVM (step 3). The socio-ecological model represents a comprehensive approach to designing, implementing and evaluating interventions which target multiple influences on behaviour [27]. The components of the proposed model are divided into four areas, i.e. a business component, a food and nutrition component, a hygiene component and a vending cart. These four areas, in turn, impact on various areas of the socio-ecological framework, i.e. intrapersonal/individual, interpersonal, the physical environment/community as well as the policy environment as displayed in Fig 3. A proposed tool has been drafted for each component, which would need further development, research and evaluation (Tools available on request from author).

## Conclusion

In literature regarding SF, hygiene considerations are often the most important issues at hand, while the nutritional aspect of foods, the business operation and the guiding legislation are often overlooked. The literature readily acknowledges the contribution that the SF trade has on the economy but fails to explore how one could go about improving the trade and growing the vendors’ knowledge and skills. This research study thus embarked on developing a SFVM that would entail nutrition, hygiene and safety, and business and operational aspects of street-food vending. This was done within a socio-ecological framework, which considers an individual as part of a greater whole. Important elements such as the individual’s physical environment, the policy environment and also basics such as the individual’s knowledge and ‘support’ environment is considered.

This proposed SFVM will hopefully be taken forward, piloted, and adapted as needed to improve the SF-vending trade. Eventually it is endeavoured to not only make the SF trade more lucrative, the vendor more knowledgeable and aware, but also to make the product sold a healthier one. In turn, this should improve the overall nutrition and health of consumers who frequently purchase SF.

### Limitations

Although this was a mixed-methodology study, which enabled a broader contextual appreciation, understanding the vending operation from the ground (the vendor) as well as from the user (the consumer) and from a regulatory point of view (government/municipal officials). The integration step in this study which was the main focus of this paper remain a qualitative process, in which bias cannot be totally excluded.

## Supporting information

S1 Questionnaire (DOCX)

S2 Questionnaire (DOCX)

S1 Checklist (DOCX)

S1 Supporting information (DOCX)

S1 Data (ACCDB)

S2 Data (ACCDB)

S3 Data (RTF)
