# Supplementary material for: The development of an evidence-based street food vending model within a socioecological framework: A guide for African countries
Source: PLoS One. 2019 Oct 22;14(10):e0223535. doi: 10.1371/journal.pone.0223535 (PMC6804966; doi:10.1371/journal.pone.0223535)
Supplement: S1 Questionnaire — (DOCX) [file pone.0223535.s001.docx]

**STREET FOOD SURVEY**


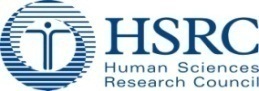


**QUESTIONNAIRE FOR VENDORS**

| ID/study number |  | |  |  |  |  |
| --- | --- | --- | --- | --- | --- | --- |
| Location: | | | | |  |  |
| Date: |  |  |  |  |  |  |
| Time: |  | |  |  |  |  |
| Interviewer: |  | | | |  |  |

**Sections A to F should be asked by the interviewer. (Tick in appropriate box)**

**Section G and H should be completed by the vendors**

**Section A: Socio-demographic information**

|  |  | | | |
| --- | --- | --- | --- | --- |
| 1. Gender (**Do not ask but tick**) | 1. Male 1 | 1. Female 2 |  | |
|  |  | | | |
| 1. How old are you? | 1. Less than 18 years | | 1 |  |
|  | 1. 18 – 24 years | | 2 |  |
|  | 1. 25 – 34 years | | 3 |  |
|  | 1. 35 – 44 years | | 4 |  |
|  | 1. 45 – 54 years | | 5 |  |
|  | 1. 55 - 64 years | | 6 |  |
|  | 1. 65 – 74 years | | 7 |  |
|  | 1. More than 75 years | | 8 |  |
|  |  | | | |
| 1. What is your marital status? | 1. Single | | 1 |  |
|  | 1. Married | | 2 |  |
|  | 1. Living with partner | | 3 |  |
|  | 1. Separated | | 4 |  |
|  | 1. Divorced | | 5 |  |
|  | 1. Widowed | | 6 |  |

| 1. What is your country of origin? | 1. South African | 1 |  |
| --- | --- | --- | --- |
|  | 1. Other (*specify*)   ..................................................... | 2 |  |
|  |  | | |
| 1. If respondent is South African, indicate race **( Do not ask but tick)** | 1. Black African | 1 |  |
|  | 1. Coloured | 2 |  |
|  | 1. Indian/Asian | 3 |  |
|  | 1. White | 4 |  |
|  | e. Not South African | 5 |  |
|  | f. Other (*specify*)  ................................................ | 6 |  |

| 1. What is your highest level of education? | - 1. Primary school | 1 |  |
| --- | --- | --- | --- |
|  | - 1. Some high school | 2 |  |
|  | - 1. Matric | 3 |  |
|  | - 1. Diploma | 4 |  |
|  | - 1. Degree | 5 |  |
|  | - 1. No schooling | 6 |  |

**Section B. Operational information**

| 1. Which days of the week do you work at the stall? | 1. Monday | Yes 1 | No 2 |
| --- | --- | --- | --- |
|  | 1. Tuesday | Yes 1 | No 2 |
|  | 1. Wednesday | Yes 1 | No 2 |
|  | 1. Thursday | Yes 1 | No 2 |
|  | 1. Friday | Yes 1 | No 2 |
|  | 1. Saturday | Yes 1 | No 2 |
|  | 1. Sunday | Yes 1 | No 2 |

| 1. When is your stall open? | Weekdays:… to…… = hrs | 1 |  |
| --- | --- | --- | --- |
|  | Saturdays:……to……… = hrs | 2 |  |
|  | Sundays:…… to……… = hrs | 3 |  |

| 1. Are you | | 1. The full owner of the stall? | | 1 |  |  |
| --- | --- | --- | --- | --- | --- | --- |
|  | | 1. A part owner of the stall? | | 2 |  |  |
|  |  | 1. Not the owner of the stall? | | 3 |  |  |
|  | |  | | | | |
| 1. What is your average income from the stall per week? | | Give in Rand…………………. | | R | | |
|  | | | | | | |
| 1. How much mark-up do you add to the stock you buy? | Give in % | | | % | | |
|  | | | | | |  |
| 1. Where do you buy supplies for the stall? | | ………………………………………  ……………………………………… | |  | |  |
|  | |  |  |  |  |  |
| 1. How do you get to the place/s where you buy supplies? | | 1. Bus | | 1 |  |  |
|  |  | 1. Train | | 2 |  |  |
|  |  | 1. Taxi | | 3 |  |  |
|  |  | 1. Car | | 4 |  |  |
|  |  | 1. Walk | | 5 |  |  |
|  |  | 1. Supplies are delivered | | 6 |  |  |
| 1. How far away is the place where you buy your stock?   If distance not known, please indicate time in minutes spent travelling e.g. 5 min walk/10 min drive etc. | | ……………………………………….Km  ………………………………………..  ……………………………………….. | | | |  |
|  | | | | | |  |
| 1. Where do you keep your stock? | | a. Home | | 1 |  |  |
|  | | b. Stall | | 2 |  |  |
|  | | c. Storeroom | | 3 |  |  |
|  | | 1. Other specify……………….. | | 4 |  |  |
|  | |  |  |  |  |  |
| 1. Do you employ anyone else at the stall? | | a. None | | 1 |  |  |
|  | | b. One | | 2 |  |  |
|  | | c. Two | | 3 |  |  |
|  | | d. More than 2 | | 4 |  |  |
|  | | | | | |  |
| 1. Do you decide what to sell in the stall? | | 1. Yes 1 | 1. No 2 |  | |  |
|  | | | | | |  |
| 1. How is money stored? | | a. In a till | | 1 |  |  |
|  | | b. In a box/pouch | | 2 |  |  |
|  | | c. In vendors pocket | | 3 |  |  |
|  | | d. Other……………… | | 4 |  |  |
|  | | | | | |  |
| 1. Is there an inventory of stock? | | 1. Yes 1 | 1. No 2 |  | |  |
|  | |  | |  | |  |

**Section C. Food items sold**

| 1. Do you sell the same items all year round at the stall? | 1. Yes 1 | 1. No 2 |  |
| --- | --- | --- | --- |

| 1. If no, which items change? | …………………………………………………………………………………………………….………………………………..……………………………………………………………………….. | 1 |  |
| --- | --- | --- | --- |
|  |  | 2 |  |
|  |  | 3 |  |

| 1. Do you sell cooked foods? | a. Yes 1 | b. No 2 |  |
| --- | --- | --- | --- |

| **If no skip to Q 5**  (i) If yes, please list cooked items: | …………………………………………………….…………………………………………………………………..……………..............................................................................................…………………………………………….………………………………………….………………………………………………………………………………………………………………………………………….………. | 1  2  3  4  5  6 |  |
| --- | --- | --- | --- |

| (ii) If yes, where do you cook them? | 1. At the site | 1 |  |
| --- | --- | --- | --- |
|  | 1. Home | 2 |  |
|  | 1. Other, specifiy………………………………………………… | 3 |  |

| (iii) Who cooks the cooked food? | 1. Self | 1 |  |
| --- | --- | --- | --- |
|  | 1. Spouse | 2 |  |
|  | 1. Other, specify…………………………………….……….. | 3 |  |

| (iv) How do you keep cooked food warm at your stall? | …………………………………………………………………………………………….………………………………………………………………………………….……………………………………………………………………………. | 1  2  3  4  5 |  |
| --- | --- | --- | --- |
|  |  |  |  |
|  |  |  |  |
|  |  |  |  |
| 4. If you have leftovers of prepared food at the end of the day what do you do with it? | a. Throw it away | 1 |  |
|  | b. Take home to eat | 2 |  |
|  | c. Sell the next day | 3 |  |

| 1. List all other items you sell at your stall?   **(List only food items)** | ...................................................................................…………………………………………………………..…………………………………………………………..…………………………………………………………..…………………………………………………………..…………………………………………………………..…………………………………………………………..…………………………………………………………..…………………………………………………………..…………………………………………………………..…………………………………………………………..…………………………………………………………..…………………………………………………………..…………………………………………………………..…………………………………………………………..…………………………………………………………..……………………………………………………………………………………………………………………………….……………………….. | 1  2  3  4  5  6  7  8  9  1011121314151617 |
| --- | --- | --- |

**Section D. Facilities available**

| 1. Does your on-site stall have access to | 1. Water? | Yes 1 | No 2 |
| --- | --- | --- | --- |
|  | 1. Electricity? | Yes 1 | No 2 |
|  | 1. Gas? | Yes 1 | No 2 |
|  | 1. Stove? | Yes 1 | No 2 |
|  | 1. Fridge? | Yes 1 | No 2 |
|  | 1. Freezer? (for food storage!) | Yes 1 | No 2 |
|  | 1. Rubbish disposal? | Yes 1 | No 2 |

| 1. Do you have facilities for hand washing at your stall? | 1. Yes 1 | 1. No 2 |  |
| --- | --- | --- | --- |

| (i) If yes, what facility is there for washing hands? **(Indicate by using time i.e. 1 min walk etc.)**  **If no, skip to Q3** | 1. Sink and tap | 1 |  |
| --- | --- | --- | --- |
|  | 1. Basin with water | 2 |  |
|  | 1. Other (specify) ………………. | 3 |  |

| 3. Where are the nearest toilet facilities? | ............................................................................……………………………………………………………… | 1 |  |
| --- | --- | --- | --- |
|  |  | 2 |  |
|  |  | 3 |  |

| 4. Is there a hand washing area at the toilet? | 1. Yes 1 | 1. No 2 |  |
| --- | --- | --- | --- |

| 5. Do you have a fire extinguisher? | 1. Yes 1 | 1. No 2 |  |
| --- | --- | --- | --- |

**Section E. Open-ended questions**

**1. If there was one thing you could change about your vending operation, what would it be?**

**………………………………………………………………………………………………………………………………………………………………………………………………………………………………………………………………………………………………………………………………………………………………………………………………………………………………………………………………………………………………………………………………………..**

**2. Do you have any problems running your vending operation? Describe these.**

**…………………………………………………………………………………………………………………………………………………………………………………………………………………………………………………………………………………………………………………………………………………………………………………………………………………………………………………………………………………………………………………………………………**

**3. Is there anything the municipality can do to help improve your business?**

**…………………………………………………………………………………………………………………………………………………………………………………………………………………………………………………………………………………………………………………………………………………………………………………………………………………………………………………………………………………………………………………………………………**

**…………………………………………………………………………………………………………………………………………………………………………………………………………………………………………………………………………………………………………………………………………………………………………………………………………………………………………………………………………………………………………………………………………**

**Section F: Certification**

| 1. Do you have a certificate of acceptability to sell street food from the DOH? | 1. Yes 1 | 1. No 2 | 1. Not needed 3 |  |
| --- | --- | --- | --- | --- |

| 1. If yes, when did you receive this? | Month: | Year: |  |
| --- | --- | --- | --- |

| 1. Do you have a permit or concession letter or a lease to sell street food? | 1. Permit | 1 |  |
| --- | --- | --- | --- |
|  | 1. Concession letter | 2 |  |
|  | 1. Lease | 3 |  |
|  | 1. None | 4 |  |

| 1. If you could choose an area to trade in, which one would it be? | **………………………………………………………………………………………………………………………………………………………** |  |
| --- | --- | --- |

| 1. How safe is the area where you trade? | 1. Very safe | 1 |  |
| --- | --- | --- | --- |
|  | 1. Safe most of the time | 2 |  |
|  | 1. Dangerous at times | 3 |  |
|  | 1. Often dangerous | 4 |  |
|  | 1. Very dangerous | 5 |  |

**Section G: Knowledge Questions**

**Instructions for completion of this section: Please answer all the questions.** Circle the letter a, b, c or d to indicate your answer. Only one letter may be circled as your answer.

**Instructions for completion of this section:** Please answer all the questions. Circle the letter a, b, c or d to indicate your answer. Only one letter may be circled as your answer.

**Fruits and vegetables**

|  |  | Response |
| --- | --- | --- |
| 1. Which vegetable will help with good eyesight? | 1. Butternut | 1 |
|  | 1. Cabbage | 2 |
|  | 1. Lettuce | 3 |
|  | 1. Cucumber | 4 |

|  |  | Response |
| --- | --- | --- |
| 1. Which fruit will help the body fight colds? | 1. Apple | 1 |
|  | 1. Mango | 2 |
|  | 1. Naartjie | 3 |
|  | 1. Peach | 4 |
|  |  | Response |
| 1. Which vegetable has the **most** fibre (roughage) | 1. Cabbage | 1 |
|  | 1. Cauliflower | 2 |
|  | 1. Green beans | 3 |
|  | 1. Lettuce | 4 |

**Fats and Oils**

|  |  | Response |
| --- | --- | --- |
| 1. Which potato has the **least** fat? | 1. Mashed potato | 1 |
|  | 1. Fried potato | 2 |
|  | 1. Boiled potato | 3 |
|  | 1. Roast potato | 4 |

|  |  | Response |
| --- | --- | --- |
| 1. Which food has the **most** fat? | 1. Atjar | 1 |
|  | 1. Mayonnaise | 2 |
|  | 1. Mustard | 3 |
|  | 1. Chakalaka | 4 |

**Starchy foods**

|  |  | Response |
| --- | --- | --- |
| 1. Why are starchy foods important to eat? | 1. Easy to digest | 1 |
|  | 1. Builds muscles | 2 |
|  | 1. Source of energy | 3 |
|  | 1. Fights diseases | 4 |

|  |  | Response |
| --- | --- | --- |
| 1. When will starchy foods make one gain weight? | 1. When eaten with meat | 1 |
|  | 1. When eaten in large amounts | 2 |
|  | 1. When eaten in the mornings | 3 |
|  | 1. When eaten with vegetables | 4 |

**Meat and milk**

|  |  | Response |
| --- | --- | --- |
| 1. How often should oily fish like pilchards and tuna be eaten? | 1. Every day | 1 |
|  | 1. Once a week | 2 |
|  | 1. Twice a week | 3 |
|  | 1. Twice a month | 4 |
|  |  | Response |
| 1. Which food is **better** for a healthy heart? | 1. Fried chicken | 1 |
|  | 1. Grilled fish | 2 |
|  | 1. Roast beef | 3 |
|  | 1. Boiled sheep brains | 4 |

**Legumes and nuts**

|  |  | Response |
| --- | --- | --- |
| 1. Which food has fibre (roughage)? | 1. Eggs | 1 |
|  | 1. Nuts | 2 |
|  | 1. Fish | 3 |
|  | 1. Chicken | 4 |
|  |  | Response |
| 1. Why can legumes like dried beans and lentils be eaten instead of meat? | 1. They have protein | 1 |
|  | 1. They have vitamins | 2 |
|  | 1. They have fat | 3 |
|  | 1. They have fibre (roughage) | 4 |

**Sugar**

|  |  | Response |
| --- | --- | --- |
| 1. Which food does **not** have added sugar? | 1. Canned apricot | 1 |
|  | 1. Apricot jam | 2 |
|  | 1. Apricot juice | 3 |
|  | 1. Fresh apricot | 4 |
|  |  | Response |
| 1. Which health problem can be caused by drinking sugary cool drinks every day? | 1. Heart disease | 1 |
|  | 1. Tuberculosis (TB) | 2 |
|  | 1. Liver disease | 3 |
|  | 1. Weight gain | 4 |

**Salt**

|  |  | Response |
| --- | --- | --- |
| 1. Which health problem can one get from too much salt? | 1. High blood pressure | 1 |
|  | 1. Liver failure | 2 |
|  | 1. Lung disease | 3 |
|  | 1. High blood sugar | 4 |
|  |  | Response |
| 1. Which has the **leas**t salt? | 1. Braai salt | 1 |
|  | 1. Stock cube | 2 |
|  | 1. Soup powder | 3 |
|  | 1. Dried herbs | 4 |

**Section H: Nutrition attitudes**

**Instructions for completion of this section:** Tick (🗸) the appropriate box for each statement to indicate whether you strongly disagree, disagree, neither agree nor disagree, agree or strongly agree with the following statements. Only one tick may be made for a statement.

|  | **Strongly Disagree** | **Disagree** | **Neither Agree Nor Disagree** | **Agree** | **Strongly Agree** |
| --- | --- | --- | --- | --- | --- |
| **Fruits and vegetables** | | | | | |
| 1. Fruit and vegetables should be eaten every day. | 1 | 2 | 3 | 4 | 5 |
| 1. Fruit and vegetables protect against illnesses. | 1 | 2 | 3 | 4 | 5 |
| 1. The number of fruit and vegetables eaten every day is important. | 1 | 2 | 3 | 4 | 5 |
| 1. It is **not** necessary to eat fruit and vegetables everyday. | 1 | 2 | 3 | 4 | 5 |
| 1. Fruit and vegetables will **not** add to good health. | 1 | 2 | 3 | 4 | 5 |

|  | **Strongly Disagree** | **Disagree** | **Neither Agree Nor Disagree** | **Agree** | **Strongly Agree** |
| --- | --- | --- | --- | --- | --- |
| **Fats and oils** | | | | | |
| 1. I look at the fat content of the food l eat. | 1 | 2 | 3 | 4 | 5 |
| 1. For good health l eat less fatty food. | 1 | 2 | 3 | 4 | 5 |

|  | **Strongly Disagree** | **Disagree** | **Neither Agree Nor Disagree** | **Agree** | **Strongly Agree** |
| --- | --- | --- | --- | --- | --- |
| **Starchy foods** | | | | | |
| 1. Starchy foods should be eaten with meals. | 1 | 2 | 3 | 4 | 5 |
| 1. Starchy food is **healthier** if it has fibre (roughage). | 1 | 2 | 3 | 4 | 5 |

|  | **Strongly Disagree** | **Disagree** | **Neither Agree Nor Disagree** | **Agree** | **Strongly Agree** |
| --- | --- | --- | --- | --- | --- |
| **Legumes and nuts** | | | | | |
| 1. Legumes like dried beans and lentils can replace meat in the diet. | 1 | 2 | 3 | 4 | 5 |
| 1. Soy mince is almost as healthy as meat. | 1 | 2 | 3 | 4 | 5 |
| 1. It is important to eat legumes like dried beans and lentils often. | 1 | 2 | 3 | 4 | 5 |

|  | **Strongly Disagree** | **Disagree** | **Neither Agree Nor Disagree** | **Agree** | **Strongly Agree** |
| --- | --- | --- | --- | --- | --- |
| **Sugar** | | | | | |
| 1. Sugar is **unhealthy** when you eat a lot of it. | 1 | 2 | 3 | 4 | 5 |
| 1. Sugar is **okay** if you use little. | 1 | 2 | 3 | 4 | 5 |
| 1. We do **not** need added sugar to be healthy. | 1 | 2 | 3 | 4 | 5 |

|  | **Strongly Disagree** | **Disagree** | **Neither Agree Nor Disagree** | **Agree** | **Strongly Agree** |
| --- | --- | --- | --- | --- | --- |
| **Salt** | | | | | |
| 1. We should **not** eat a lot of salty food. | 1 | 2 | 3 | 4 | 5 |
| 1. I worry about the amount of salt in food. | 1 | 2 | 3 | 4 | 5 |
| 1. Food can taste good with only a little salt added. | 1 | 2 | 3 | 4 | 5 |
| 1. Food only tastes good if a lot of salt is added. | 1 | 2 | 3 | 4 | 5 |
| 1. I enjoy salty food. | 1 | 2 | 3 | 4 | 5 |
|  | | | | | |

**Thank you for participating in this study and completing this questionnaire.**


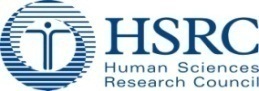


**Observational Checklist Street-Food Vendors**

| SECTION A: General | | | | | | | | |  |  | *Office use* | | | | | | | |
| --- | --- | --- | --- | --- | --- | --- | --- | --- | --- | --- | --- | --- | --- | --- | --- | --- | --- | --- |
|  | | | | | | | | |  |  |  | | | | | | | |
| ID/study number | | | | | | | | |  |  |  |  |  |  |  |  |  |  |
|  | | | | | | | | |  |  |  |  |  |  |  |  |  |  |
| 1. Location: | | | | | | |  |  |  |  |  |  |  |  |  |  |  |  |
|  | | | | | | | | |  |  |  |  |  |  |  |  |  |  |
| 2. Date: | | D | D | M | M | | Y | Y |  |  |  |  |  |  |  |  |  |  |
|  | | | | | | | | |  |  |  |  |  |  |  |  |  |  |
| 3. Time: | | | |  |  | |  |  |  |  |  |  |  |  |  |  |  |  |
|  | | | | | | | | |  |  |  |  |  |  |  |  |  |  |
| 4. Interviewer: | | | | | | |  |  |  |  |  |  |  |  |  |  |  |  |
|  | | | | | | | | |  |  |  |  |  |  |  |  |  |  |
| 5. Does vendor serve **cooked** food like pap, vetkoek, soup, meat? | | | | | | | Yes 1 | No 2 |  |  |  |  |  |  |  |  |  |  |
| 6. Does the vendor serve **baked** foods like scones and muffins? | | | | | | | Yes 1 | No 2 |  |  |  |  |  |  |  |  |  |  |
| 7. Does the vendor sell **ready to eat** foods like biscuits, crisps? | | | | | | | Yes 1 | No 2 |  |  |  |  |  |  |  |  |  |  |
| 8. Does the vendor sell **beverages** (drinks cold and hot drinks)? | | | | | | | Yes 1 | No 2 |  |  |  |  |  |  |  |  |  |  |
| 9**. Food sold by vendors:** | | | | | | | | |  |  |  |  |  |  |  |  |  |  |
| Porridge and beef/chicken | | | | | | | Yes 1 | No 2 |  |  |  |  |  |  |  |  |  |  |
| Rice and beef/chicken | | | | | | | Yes 1 | No 2 |  |  |  |  |  |  |  |  |  |  |
| White bread sandwiches | | | | | | | Yes 1 | No 2 |  |  |  |  |  |  |  |  |  |  |
| Brown bread sandwiches | | | | | | | Yes 1 | No 2 |  |  |  |  |  |  |  |  |  |  |
| Vetkoek(plain) | | | | | | | Yes 1 | No 2 |  |  |  |  |  |  |  |  |  |  |
| Vetkoek with protein filling | | | | | | | Yes 1 | No 2 |  |  |  |  |  |  |  |  |  |  |
| Gatsby | | | | | | | Yes 1 | No 2 |  |  |  |  |  |  |  |  |  |  |
| Kota | | | | | | | Yes 1 | No 2 |  |  |  |  |  |  |  |  |  |  |
| Vegetables | | | | | | | Yes 1 | No 2 |  |  |  |  |  |  |  |  |  |  |
| Salad | | | | | | | Yes 1 | No 2 |  |  |  |  |  |  |  |  |  |  |
| Fruit | | | | | | | Yes 1 | No 2 |  |  |  |  |  |  |  |  |  |  |
| Rice | | | | | | | Yes 1 | No 2 |  |  |  |  |  |  |  |  |  |  |
| Porridge | | | | | | | Yes 1 | No 2 |  |  |  |  |  |  |  |  |  |  |
| Chicken | | | | | | | Yes 1 | No 2 |  |  |  |  |  |  |  |  |  |  |
| Beef | | | | | | | Yes 1 | No 2 |  |  |  |  |  |  |  |  |  |  |
| Mogudo/mutton | | | | | | | Yes 1 | No 2 |  |  |  |  |  |  |  |  |  |  |
| Fish | | | | | | | Yes 1 | No 2 |  |  |  |  |  |  |  |  |  |  |
| Hotdogs | | | | | | | Yes 1 | No 2 |  |  |  |  |  |  |  |  |  |  |
| Burgers | | | | | | | Yes 1 | No 2 |  |  |  |  |  |  |  |  |  |  |
| Soup | | | | | | | Yes 1 | No 2 |  |  |  |  |  |  |  |  |  |  |
| Hot chips | | | | | | | Yes 1 | No 2 |  |  |  |  |  |  |  |  |  |  |
| Biscuits/cakes/muffins | | | | | | | Yes 1 | No 2 |  |  |  |  |  |  |  |  |  |  |
| Sweets | | | | | | | Yes 1 | No 2 |  |  |  |  |  |  |  |  |  |  |
| Chocolates | | | | | | | Yes 1 | No 2 |  |  |  |  |  |  |  |  |  |  |
| Chips/crisps | | | | | | | Yes 1 | No 2 |  |  |  |  |  |  |  |  |  |  |
| Tea/coffee | | | | | | | Yes 1 | No 2 |  |  |  |  |  |  |  |  |  |  |
| Soft drinks | | | | | | | Yes 1 | No 2 |  |  |  |  |  |  |  |  |  |  |
| Juices | | | | | | | Yes 1 | No 2 |  |  |  |  |  |  |  |  |  |  |
| Water | | | | | | | Yes 1 | No 2 |  |  |  |  |  |  |  |  |  |  |
|  | | | | | | | | |  |  |  |  |  |  |  |  |  |  |
| Other: |  | | | | | | Yes 1 | No 2 |  |  |  |  |  |  |  |  |  |  |
|  | | | | | | | | |  |  |  |  |  |  |  |  |  |  |
|  | | | | | | | | |  |  |  |  |  |  |  |  |  |  |
| SECTION B: The vendor’s site | | | | | | | | |  |  | *Office use* | | | | | | | |
| 1. What does the street-food vendor site look like?  : | | | | | | | | |  |  |  |  |  |  |  |  |  |  |
| Does it have a roof cover? | | | | | | Yes 1 | | No 2 |  |  |  |  |  |  |  |  |  |  |
| Does it have walls? | | | | | | Yes 1 | | No 2 |  |  |  |  |  |  |  |  |  |  |
| Does it have a counter? | | | | | | Yes 1 | | No 2 |  |  |  |  |  |  |  |  |  |  |
| Is it a caravan? | | | | | | Yes 1 | | No 2 |  |  |  |  |  |  |  |  |  |  |
| Is food placed on the pavement? | | | | | | Yes 1 | | No 2 |  |  |  |  |  |  |  |  |  |  |
| Is it a kiosk with sides & roof? | | | | | | Yes 1 | | No 2 |  |  |  |  |  |  |  |  |  |  |
| Is it a shipping container? | | | | | | Yes 1 | | No 2 |  |  |  |  |  |  |  |  |  |  |
| Is it a temporary stall packed up after use? | | | | | | Yes 1 | | No 2 |  |  |  |  |  |  |  |  |  |  |
|  | | | | | | | | |  |  |  |  |  |  |  |  |  |  |
| Other: | Specify details | | | | |  | | |  |  |  |  |  |  |  |  |  |  |
|  | | | | | | | | |  |  |  |  |  |  |  |  |  |  |
|  | | | | | | | | |  |  |  |  |  |  |  |  |  |  |

| SECTION C: Hygiene status of vendors and site | | | |  |  | *Office use* | | | | | | | | |
| --- | --- | --- | --- | --- | --- | --- | --- | --- | --- | --- | --- | --- | --- | --- |
| 1. Hygiene status of vendors: | | | |  |  |  |  | |  |  |  |  |  |  |
| Short, clean nails? | | Yes 1 | No 2 |  |  |  |  | |  |  |  |  |  |  |
| Hands free of sores? | | Yes 1 | No 2 |  |  |  |  | |  |  |  |  |  |  |
| Smoking while working with food? | | Yes 1 | No 2 |  |  |  |  | |  |  |  |  |  |  |
| Jewellery/bangles on hands? | | Yes 1 | No 2 |  |  |  |  | |  |  |  |  |  |  |
| Handling money without washing hands in-between? | | Yes 1 | No 2 |  |  |  |  | |  |  |  |  |  |  |
| Vendor appears to have a cold/runny nose? | | Yes 1 | No 2 |  |  |  |  | |  |  |  |  |  |  |
|  | | | |  |  |  |  | |  |  |  |  |  |  |
| Other: |  |  | |  |  |  |  | |  |  |  |  |  |  |
|  | | | |  |  |  |  | |  |  |  |  |  |  |
| 2. Protective clothing worn by vendor: Full apron? | | Yes 1 | No 2 |  |  |  | |  | |  |  |  |  |  |
| Half apron? | | Yes 1 11 | No 2 |  |  |  | |  | |  |  |  |  |  |
| Overall? | | Yes1 | No 2 |  |  |  | |  | |  |  |  |  |  |
| Hair-covering? | | Yes1 | No 2 |  |  |  | |  | |  |  |  |  |  |
| Gloves? | | Yes 1 | No 2 |  |  |  | |  | |  |  |  |  |  |
| 3. Does apron/overall appear to be clean? | | Yes 1 | No 2 |  |  |  | |  | |  |  |  |  |  |
| 4. Is there a basin or tap for washing hands? | | Yes1 | No 2 |  |  |  | |  | |  |  |  |  |  |
| 5. Is there soap for washing hands? | | Yes 1 | No 2 |  |  |  | |  | |  |  |  |  |  |
| 6. Is there any antiseptic solution for washing? | | Yes 1 | No 2 |  |  |  | |  | |  |  |  |  |  |
| 7. Is there a cloth for drying hands? | | Yes 1 | No 2 |  |  |  | |  | |  |  |  |  |  |
| 8. Is there a clean washing cloth/sponge for washing utensils/food? | | Yes1 | No 2 |  |  |  | |  | |  |  |  |  |  |
| 9. Is there a clean drying cloth for utensils/food? | | Yes 1 | No 2 |  |  |  | |  | |  |  |  |  |  |
| 10. Any other comments about hygiene?................................................. | | | |  |  |  |  | |  |  |  |  | | |
| ………………………………………………………………………………………………… | | | |  |  |  |  | |  |  |  |  | | |
|  | | | |  |  |  |  | |  |  |  |  |  |  |
|  | | | |  |  |  |  | |  |  |  |  |  |  |
| The next questions are **ONLY if the vendor prepares food at the stall** | | | |  |  |  |  | |  |  |  |  | | |
|  | | | |  |  |  |  | |  |  |  |  |  |  |
| 11. Surface on which food is prepared? | |  | |  |  |  |  | |  |  |  |  |  |  |
| Plastic | | Yes 1 | No 2 |  |  |  |  | |  |  |  |  |  |  |
| Wood | | Yes 1 | No |  |  |  |  | |  |  |  |  |  |  |
| Metal | | Yes 1 | No |  |  |  |  | |  |  |  |  |  |  |
| Cement | | Yes 1 | No |  |  |  |  | |  |  |  |  |  |  |
| Cardboard/newspaper | | Yes 1 | No |  |  |  |  | |  |  |  |  |  |  |
| Glass | | Yes 1 | No |  |  |  |  | |  |  |  |  |  |  |
| Cloth | | Yes 1 | No |  |  |  |  | |  |  |  |  |  |  |
|  | | | |  |  |  |  | |  |  |  |  |  |  |
| The next questions are **ONLY if the vendor prepares food at the stall (**CONT**)** | | | |  |  |  |  | |  |  |  |  | | |
|  | | | |  |  |  |  | |  |  |  |  |  |  |
| 12. Does the vendor use separate utensils for cooked and raw food? | | Yes 1 | No 2 |  |  |  |  | |  |  |  |  |  |  |
| 13. Does there appear to be adequate take away containers? | | Yes 1 | No 2 |  |  |  |  | |  |  |  |  |  |  |
| 14. Does there appear to be adequate cutlery? | | Yes 1 | No 2 |  |  |  |  | |  |  |  |  |  |  |
| 15. Does the cutlery appear to be clean?  1  18. | | Yes 1 | No 2 |  |  |  |  | |  |  |  |  |  |  |
| 16. Does the vendor use his hands? | | Yes 1 | No 2 |  |  |  |  | |  |  |  |  |  |  |
| 17. Does the vendor use gloves? | | Yes 1 | No 2 |  |  |  |  | |  |  |  |  |  |  |
| 18. Does the vendor use cutlery to pick up food? | | Yes 1 | No 2 |  |  |  |  | |  |  |  |  |  |  |
| 19. Is cooked food kept covered? | | Yes1 | No 2 |  |  |  |  | |  |  |  |  |  |  |
| 20. Is cooked food kept warm? | | Yes1 | No 2 |  |  |  |  | |  |  |  |  |  |  |
| 21. If yes, how? ..................................................................................... | | | | Yes1 | No 2 |  |  | |  |  |  |  |  |  |
