# Supplementary material for: The development of an evidence-based street food vending model within a socioecological framework: A guide for African countries
Source: PLoS One. 2019 Oct 22;14(10):e0223535. doi: 10.1371/journal.pone.0223535 (PMC6804966; doi:10.1371/journal.pone.0223535)
Supplement: S2 Questionnaire — (DOCX) [file pone.0223535.s002.docx]

**STREET FOOD SURVEY**


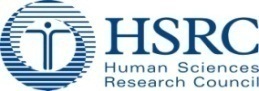


**QUESTIONNAIRE FOR CONSUMERS**

**SECTION A: Socio-demographic information**

| ID/study number |  | |  |  |  |  |
| --- | --- | --- | --- | --- | --- | --- |
| Location: | | | | |  |  |
| Date: |  |  |  |  |  |  |
| Time: |  | |  |  |  |  |
| Interviewer: |  | | | |  |  |

| 1. Gender (Do not ask but tick) | 1. Male | 1 |
| --- | --- | --- |
|  | 1. Female | 2 |

| 1. Age in years | 1. 13-17 | 1 |
| --- | --- | --- |
|  | 1. 18 - 24 | 2 |
|  | 1. 25 - 34 | 3 |
|  | 1. 35 - 44 | 4 |
|  | 1. 45 – 54 | 5 |
|  | 1. 55 - 64 | 6 |
|  | 1. 65 + | 7 |

| 1. Marital status | 1. Single | 1 |
| --- | --- | --- |
|  | 1. Married | 2 |
|  | 1. Living as married | 3 |
|  | 1. Separated | 4 |
|  | 1. Divorced | 5 |
|  | 1. Widowed | 6 |

| 1. What is your country of origin? | 1. South African | | 1 |
| --- | --- | --- | --- |
|  | 1. Other (*specify*) ……………………………………………… | | 2 |
| 1. If respondent is South African, indicate race | 1. Black African | 1 | |
| **( Do not ask but tick)** | 1. Coloured | 2 | |
|  | 1. Indian/Asian | 3 | |
|  | 1. White | 4 | |
|  | 1. Other (*specify*)   ................................................................ | 5 | |
| 1. Employment status | 1. Unemployed, | 1 | |
|  | 1. Full time employed, | 2 | |
|  | 1. Part time employed, | 3 | |
|  | 1. Scholar / student / training, | 4 | |
|  | 1. Self-employed | 5 | |
|  | 1. Other (specify) …………………………... | 6 | |

| 1. Level of monthly income | 1. < R3 000, | 1 |
| --- | --- | --- |
|  | 1. R3 000-R4 000, | 2 |
|  | 1. R4 000-R6 000, | 3 |
|  | 1. > R6 000 | 4 |
|  | 1. Student | 5 |
|  | 1. Unemployed | 6 |

| 1. Highest level education | 1. Primary school | 1 |
| --- | --- | --- |
|  | 1. Some high school | 2 |
|  | 1. Matric | 3 |
|  | 1. Diploma | 4 |
|  | 1. Degree | 5 |
|  | 1. No Schooling | 6 |

| 1. Main mode of transport | - 1. Train, | 1 |
| --- | --- | --- |
|  | - 1. Bus, | 2 |
|  | - 1. Taxi, | 3 |
|  | - 1. Car | 4 |

**SECTION B. Purchasing habits**

| 1. How often do you buy food/snack items/drinks from vendors/street sellers? | - 1. Almost every day; | 1 |
| --- | --- | --- |
|  | - 1. 2 to 3x a week, | 2 |
|  | - 1. About once a week; | 3 |
|  | - 1. About once or twice a month; | 4 |
|  | - 1. Never | 5 |

| 1. What time of day do you usually buy food/snack items/drinks from vendors/street sellers? | - 1. Before 10am | 1 |
| --- | --- | --- |
|  | - 1. Between 10am and 12pm | 2 |
|  | - 1. Between 12pm and 3pm | 3 |
|  | - 1. Between 3pm and 6pm | 4 |
|  | - 1. After 6pm | 5 |

| 1. Where do you usually buy your breakfast/snack/lunch from vendors/street sellers? | 1. Near home | 1 |
| --- | --- | --- |
|  | 1. Near work | 2 |
|  | 1. Near school | 3 |
|  | 1. Near college | 4 |
|  | 1. Other (specify)……….. | 5 |

1 2 3

|  |  |  |
| --- | --- | --- |

1. About how much money do you spend a week on street food in rands?

OR

About how much money do you spend a month on street foods in rands? 1 2 3

|  |  |  |
| --- | --- | --- |

**SECTION C. Consumption Preferences**

| 1. Which types of foods do you buy most often from vendors / street sellers? (Can give more than 1 answer) | 1. Fruit, | 1 |
| --- | --- | --- |
|  | 1. cold drinks, | 2 |
|  | 1. crisps; | 3 |
|  | 1. biscuits; | 4 |
|  | 1. sweets; | 5 |
|  | 1. chocolates; | 6 |
|  | 1. cooked food; | 7 |
|  | 1. peanuts; | 8 |
|  | 1. fruit juice; | 9 |
|  | 1. Other (specify)…………………………… | 10 |

1. If you buy cooked food, what is your favourite cooked street food (**If no, skip to question 5)** (specify)…………………………………………………………………………………………………………….

| 1. What does it cost? | - 1. < R10 | 1 |
| --- | --- | --- |
|  | - 1. R10 – R20 | 2 |
|  | - 1. R20 – R30 | 3 |
|  | - 1. R30 – R40 | 4 |
|  | - 1. > R40 | 5 |

| 1. Would you like vendors to sell healthier foods? | - 1. Yes | 1 |
| --- | --- | --- |
|  | - 1. No | 2 |

| 1. Which of the following would you be willing to buy from a vendor / street seller? (Can give more than 1 answer) | - 1. Milk, or milk drinks; | 1 |
| --- | --- | --- |
|  | - 1. yoghurt, | 2 |
|  | - 1. yoghurt and muesli; | 3 |
|  | - 1. yoghurt and fruit; | 4 |
|  | - 1. nuts, | 5 |
|  | - 1. fresh fruit juice; | 6 |
|  | - 1. fresh vegetable juice ie. carrot juice; | 7 |
|  | - 1. salad; | 8 |
|  | - 1. fruit; | 9 |
|  | - 1. fruit salad; | 10 |
|  | - 1. dried fruits; | 11 |
|  | - 1. peanuts and raisins; | 12 |
|  | - 1. cooked vegetables eg mealie, | 13 |
|  | - 1. vegetable skewers; | 14 |
|  | - 1. fruit skewers; | 15 |
|  | - 1. baked potato; | 16 |
|  | - 1. whole wheat sandwich; | 17 |
|  | - 1. meat or chicken cooked with vegetables (not fried); | 18 |
|  | - 1. veggie burgers; | 19 |
|  | - 1. high fibre muffins; | 20 |
|  | - 1. pita bread with salad fillings; | 21 |
|  | - 1. wraps with healthy fillings | 22 |

| 1. Do you ever purchase fruit from street food vendors? | - 1. Yes | 1 |
| --- | --- | --- |
|  | - 1. No | 2 |

| 1. How often? | - 1. Every day | 1 |
| --- | --- | --- |
|  | - 1. 2-3 times /week | 2 |
|  | - 1. 2-3 times /month | 3 |
|  | - 1. Hardly ever/never | 4 |
| 8. Do you ever purchase vegetables from street food vendors? | a. Yes |  |
|  | b. No |  |
|  |  |  |
| 9. How often? | a. Every day | 1 |
|  | b. 2-3 times /week | 2 |
|  | c. 2-3 times /month | 3 |
|  | d. Hardly ever/never | 4 |

**Section D: Knowledge Questions**

**Instructions for completion of this section: Please answer all the questions.** Circle the letter a, b, c or d to indicate your answer. Only one letter may be circled as your answer.

**Fruits and vegetables**

|  |  | Response |
| --- | --- | --- |
| 1. Which vegetable will help with good eyesight? | 1. Butternut | 1 |
|  | 1. Cabbage | 2 |
|  | 1. Lettuce | 3 |
|  | 1. Cucumber | 4 |

|  |  | Response |
| --- | --- | --- |
| 1. Which fruit will help the body fight colds? | 1. Apple | 1 |
|  | 1. Mango | 2 |
|  | 1. Naartjie | 3 |
|  | 1. Peach | 4 |
|  |  | Response |
| 1. Which vegetable has the **most** fibre (roughage) | 1. Cabbage | 1 |
|  | 1. Cauliflower | 2 |
|  | 1. Green beans | 3 |
|  | 1. Lettuce | 4 |

**Fats and Oils**

|  |  | Response |
| --- | --- | --- |
| 1. Which potato has the **least** fat? | 1. Mashed potato | 1 |
|  | 1. Fried potato | 2 |
|  | 1. Boiled potato | 3 |
|  | 1. Roast potato | 4 |

|  |  | Response |
| --- | --- | --- |
| 1. Which food has the **most** fat? | 1. Atjar | 1 |
|  | 1. Mayonnaise | 2 |
|  | 1. Mustard | 3 |
|  | 1. Chakalaka | 4 |

**Starchy foods**

|  |  | Response |
| --- | --- | --- |
| 1. Why are starchy foods important to eat? | 1. Easy to digest | 1 |
|  | 1. Builds muscles | 2 |
|  | 1. Source of energy | 3 |
|  | 1. Fights diseases | 4 |
|  |  | Response |
| 1. When will starchy foods make one gain weight? | 1. When eaten with meat | 1 |
|  | 1. When eaten in large amounts | 2 |
|  | 1. When eaten in the mornings | 3 |
|  | 1. When eaten with vegetables | 4 |

**Meat and milk**

|  |  | Response |
| --- | --- | --- |
| 1. How often should oily fish like pilchards and tuna be eaten? | 1. Every day | 1 |
|  | 1. Once a week | 2 |
|  | 1. Twice a week | 3 |
|  | 1. Twice a month | 4 |
|  |  | Response |
| 1. Which food is **better** for a healthy heart? | 1. Fried chicken | 1 |
|  | 1. Grilled fish | 2 |
|  | 1. Roast beef | 3 |
|  | 1. Boiled sheep brains | 4 |

**Legumes and nuts**

|  |  | Response |
| --- | --- | --- |
| 1. Which food has fibre (roughage)? | 1. Eggs | 1 |
| [Fibre (roughage) helps with constipation] | 1. Nuts | 2 |
|  | 1. Fish | 3 |
|  | 1. Chicken | 4 |
|  |  | Response |
| 1. Why can legumes like dried beans and lentils be eaten instead of meat? | 1. They have protein | 1 |
|  | 1. They have vitamins | 2 |
|  | 1. They have fat | 3 |
|  | 1. They have fibre (roughage) | 4 |

**Sugar**

|  |  | Response |
| --- | --- | --- |
| 1. Which food does **not** have added sugar? | 1. Canned apricot | 1 |
|  | 1. Apricot jam | 2 |
|  | 1. Apricot juice | 3 |
|  | 1. Fresh apricot | 4 |

|  |  | Response |
| --- | --- | --- |
| 1. Which health problem can be caused by drinking sugary cool drinks every day? | 1. Heart disease | 1 |
|  | 1. Tuberculosis (TB) | 2 |
|  | 1. Liver disease | 3 |
|  | 1. Weight gain | 4 |

**Salt**

|  |  | Response |
| --- | --- | --- |
| 1. Which health problem can one get from too much salt? | 1. High blood pressure | 1 |
|  | 1. Liver failure | 2 |
|  | 1. Lung disease | 3 |
|  | 1. High blood sugar | 4 |
|  |  | Response |
| 1. Which has the **leas**t salt? | 1. Braai salt | 1 |
|  | 1. Stock cube | 2 |
|  | 1. Soup powder | 3 |
|  | 1. Dried herbs | 4 |

**Section E: Nutrition attitudes**

**Instructions for completion of this section:** Tick (🗸) the appropriate box for each statement to indicate whether you strongly disagree, disagree, neither agree nor disagree, agree or strongly agree with the following statements. Only one tick may be made for a statement.

|  | **Strongly Disagree** | **Disagree** | **Neither Agree Nor Disagree** | **Agree** | **Strongly Agree** |
| --- | --- | --- | --- | --- | --- |
| **Fruits and vegetables** | | | | | |
| 1. Fruit and vegetables should be eaten every day. | 1 | 2 | 3 | 4 | 5 |
| 1. Fruit and vegetables protect against illnesses. | 1 | 2 | 3 | 4 | 5 |
| 1. The number of fruit and vegetables eaten every day is important. | 1 | 2 | 3 | 4 | 5 |
| 1. It is **not** necessary to eat fruit and vegetables everyday. | 1 | 2 | 3 | 4 | 5 |
| 1. Fruit and vegetables will **not** add to good health. | 1 | 2 | 3 | 4 | 5 |
|  | **Strongly Disagree** | **Disagree** | **Neither Agree Nor Disagree** | **Agree** | **Strongly Agree** |
| **Fats and oils** | | | | | |
| 1. I look at the fat content of the food l eat. | 1 | 2 | 3 | 4 | 5 |
| 1. For good health l eat less fatty food. | 1 | 2 | 3 | 4 | 5 |
|  | **Strongly Disagree** | **Disagree** | **Neither Agree Nor Disagree** | **Agree** | **Strongly Agree** |
| **Starchy foods** | | | | | |
| 1. Starchy foods should be eaten with meals. | 1 | 2 | 3 | 4 | 5 |
| 1. Starchy food is **healthier** if it has fibre (roughage). | 1 | 2 | 3 | 4 | 5 |

|  | **Strongly Disagree** | **Disagree** | **Neither Agree Nor Disagree** | **Agree** | **Strongly Agree** |
| --- | --- | --- | --- | --- | --- |
| **Legumes and nuts** | | | | | |
| 1. Legumes like dried beans and lentils can replace meat in the diet. | 1 | 2 | 3 | 4 | 5 |
| 1. Soy mince is almost as healthy as meat. | 1 | 2 | 3 | 4 | 5 |
| 1. It is important to eat legumes like dried beans and lentils often. | 1 | 2 | 3 | 4 | 5 |

|  | **Strongly Disagree** | **Disagree** | **Neither Agree Nor Disagree** | **Agree** | **Strongly Agree** |
| --- | --- | --- | --- | --- | --- |
| **Sugar** | | | | | |
| 1. Sugar is **unhealthy** when you eat a lot of it. | 1 | 2 | 3 | 4 | 5 |
| 1. Sugar is **okay** if you use little. | 1 | 2 | 3 | 4 | 5 |
| 1. We do **not** need added sugar to be healthy. | 1 | 2 | 3 | 4 | 5 |

|  | **Strongly Disagree** | **Disagree** | **Neither Agree Nor Disagree** | **Agree** | **Strongly Agree** |
| --- | --- | --- | --- | --- | --- |
| **Salt** | | | | | |
| 1. We should **not** eat a lot of salty food. | 1 | 2 | 3 | 4 | 5 |
| 1. I worry about the amount of salt in food. | 1 | 2 | 3 | 4 | 5 |
| 1. Food can taste good with only a little salt added. | 1 | 2 | 3 | 4 | 5 |
| 1. Food only tastes good if a lot of salt is added. | 1 | 2 | 3 | 4 | 5 |
| 1. I enjoy salty food. | 1 | 2 | 3 | 4 | 5 |
|  | | | | | |

24 Hour Recall

Please list all the foods you ate yesterday

| Please describe the foods (meals and snacks and drinks) you ate yesterday during the day and night | | | | | |
| --- | --- | --- | --- | --- | --- |
| **Breakfast** | **Mid-morning** | **Lunch** | **Mid afternoon** | **Supper** | **After supper** |
|  |  |  |  |  |  |

**Thank You for Completing This Questionnaire**

**List of Food groups to be completed by the field worker**

|  | **Group** | **Foods** | **Code** |
| --- | --- | --- | --- |
| 1 | Cereals | Corn/maize/samp, rice, wheat, sorghum, porridge, phutu, bread, pasta, breakfast cereals, oats, Mabella, Morvite, fortified cereals | Yes…… 1  No....… 2 |
| 2 | White roots and tubers | Potato, white sweet potato | Yes… … 1  No... ...2 |
| 3 | Yellow/orange vegetables | Carrot, butternut, pumpkin, orange-fleshed sweet potato | Yes…… 1  No... . 2 |
| 4 | Dark-green leaves | Spinach, imifino, morogo | Yes…....1  No..……2 |
| 5 | Vegetables other than dark-green leafy and yellow/orange | Beetroot, brinjals, broccoli, brussels sprouts, cabbage, cauliflower, gem squash, green beans, onion, peas, tomato, turnip, thepe | Yes.…...1  No ….…2 |
| 6 | Yellow / orange fruits | Apricot, mango, pawpaw, sweet melon, yellow flesh peach, yellow flesh plums, 100% fruit juice made from these | Yes……..1  No ….…2 |
| 7 | Fruit other than yellow / orange fleshed | Apple, avocado, banana, berries, fig, granadilla, grape, grapefruit, guava, lemon, litchi, maroela, melon, orange, naartjie, peach, pear, pineapple, plum, strawberry, watermelon, 100% fruit juice made from these | Yes…....1  No..……2 |
| 8 | Organ meat (offal) | Liver, kidney, heart, spleen, lungs, chicken giblets, malomogudo (offal), intestines | Yes…....1  No.….…2 |
| 9 | Meat and poultry (flesh meats) | Beef, goat, lamb, mutton, pork, venison, game, chicken, birds, ostrich, insects, mopani worms, chicken head/feet, sheep head | Yes…....1  No.….…2 |
| 10 | Eggs | Any type of egg | Yes…....1  No.….…2 |
| 11 | Fish and seafood | Fresh, frozen fish or canned fish (sardines, pilchards, tuna), dried fish, shellfish | Yes…....1  No.….…2 |
| 12 | Legumes, nuts and seeds | Dried beans, dried peas, lentils, nuts, peanuts, seeds (or foods made from these e.g. peanut butter) | Yes…....1  No.….…2 |
| 13 | Milk and milk products | Milk, sour milk, cheese, yogurt, custard, or any other milk products, or any drinks made with milk eg. cocoa | Yes…....1  No.….…2 |
| 14 | Fats and oils | Oils, fats, margarine or butter added to foods or used for cooking | Yes…....1  No.….…2 |
| 16 | Sugars and sweets | Sugar, sweets, chocolates, cake and sweetened biscuits, honey, jam, sugar sweetened drinks e.g. cold drinks, sugary foods, sweetened condensed milk | Yes……..1  No.... …2 |
| 17 | Spices and condiments | Spices (salt, pepper, etc), condiments (e.g. chutney, tomato sauce) | Yes…....1  No…..…2 |
| 18 | Drinks | Coffee, tea | Yes…....1  No....…2 |
| 19 | Drinks | Alcoholic drinks | Yes……..1  No........2 |
| 20 | Drinks | Cold drinks (except diet cold drinks) and sweetened beverages | Yes……..1  No.....…2 |
| 21 | Snacks | Chips | Yes……..1  No.....…2 |
| 22 | Spreads | Fish paste, sandwich spread | Yes……..1  No.....…2 |
| 23 | Other | Anything not listed as part of other food groups | Yes……..1  No.....…2 |
