# Supplementary material for: The development of an evidence-based street food vending model within a socioecological framework: A guide for African countries
Source: PLoS One. 2019 Oct 22;14(10):e0223535. doi: 10.1371/journal.pone.0223535 (PMC6804966; doi:10.1371/journal.pone.0223535)
Supplement: S1 Checklist — (DOCX) [file pone.0223535.s003.docx]

**STREET FOOD STUDY**


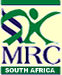

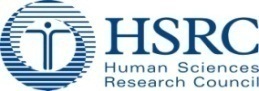


**Observational Checklist Street-Food Vendors (ADDENDUM B)**

| SECTION A: General | | | | | | | | |  |  | *Office* *use* | | | | | | | |
| --- | --- | --- | --- | --- | --- | --- | --- | --- | --- | --- | --- | --- | --- | --- | --- | --- | --- | --- |
|  | | | | | | | | |  |  |  | | | | | | | |
| ID/study number | | | | | | | | |  |  |  |  |  |  |  |  |  |  |
|  | | | | | | | | |  |  |  |  |  |  |  |  |  |  |
| 1. Location: | | | | | | |  |  |  |  |  |  |  |  |  |  |  |  |
|  | | | | | | | | |  |  |  |  |  |  |  |  |  |  |
| 2. Date: | | D | D | M | M | | Y | Y |  |  |  |  |  |  |  |  |  |  |
|  | | | | | | | | |  |  |  |  |  |  |  |  |  |  |
| 3. Time: | | | |  |  | |  |  |  |  |  |  |  |  |  |  |  |  |
|  | | | | | | | | |  |  |  |  |  |  |  |  |  |  |
| 4. Interviewer: | | | | | | |  |  |  |  |  |  |  |  |  |  |  |  |
|  | | | | | | | | |  |  |  |  |  |  |  |  |  |  |
| 5. Does vendor serve **cooked** food like pap, vetkoek, soup, meat? | | | | | | | Yes 1 | No 2 |  |  |  |  |  |  |  |  |  |  |
| 6. Does the vendor serve **baked** foods like scones and muffins? | | | | | | | Yes 1 | No 2 |  |  |  |  |  |  |  |  |  |  |
| 7. Does the vendor sell **ready to eat** foods like biscuits, crisps? | | | | | | | Yes 1 | No 2 |  |  |  |  |  |  |  |  |  |  |
| 8. Does the vendor sell **beverages**? | | | | | | | Yes 1 | No 2 |  |  |  |  |  |  |  |  |  |  |
| 9**. Food sold by vendors:** | | | | | | | | |  |  |  |  |  |  |  |  |  |  |
| Porridge and beef/chicken | | | | | | | Yes 1 | No 2 |  |  |  |  |  |  |  |  |  |  |
| Rice and beef/chicken | | | | | | | Yes 1 | No 2 |  |  |  |  |  |  |  |  |  |  |
| White bread sandwiches | | | | | | | Yes 1 | No 2 |  |  |  |  |  |  |  |  |  |  |
| Brown bread sandwiches | | | | | | | Yes 1 | No 2 |  |  |  |  |  |  |  |  |  |  |
| Vetkoek(plain) | | | | | | | Yes 1 | No 2 |  |  |  |  |  |  |  |  |  |  |
| Vetkoek with protein filling | | | | | | | Yes 1 | No 2 |  |  |  |  |  |  |  |  |  |  |
| Gatsby | | | | | | | Yes 1 | No 2 |  |  |  |  |  |  |  |  |  |  |
| Kota | | | | | | | Yes 1 | No 2 |  |  |  |  |  |  |  |  |  |  |
| Vegetables | | | | | | | Yes 1 | No 2 |  |  |  |  |  |  |  |  |  |  |
| Salad | | | | | | | Yes 1 | No 2 |  |  |  |  |  |  |  |  |  |  |
| Fruit | | | | | | | Yes 1 | No 2 |  |  |  |  |  |  |  |  |  |  |
| Rice | | | | | | | Yes 1 | No 2 |  |  |  |  |  |  |  |  |  |  |
| Porridge | | | | | | | Yes 1 | No 2 |  |  |  |  |  |  |  |  |  |  |
| Chicken | | | | | | | Yes 1 | No 2 |  |  |  |  |  |  |  |  |  |  |
| Beef | | | | | | | Yes 1 | No 2 |  |  |  |  |  |  |  |  |  |  |
| Mogudo/mutton | | | | | | | Yes 1 | No 2 |  |  |  |  |  |  |  |  |  |  |
| Fish | | | | | | | Yes 1 | No 2 |  |  |  |  |  |  |  |  |  |  |
| Hotdogs | | | | | | | Yes 1 | No 2 |  |  |  |  |  |  |  |  |  |  |
| Burgers | | | | | | | Yes 1 | No 2 |  |  |  |  |  |  |  |  |  |  |
| Soup | | | | | | | Yes 1 | No 2 |  |  |  |  |  |  |  |  |  |  |
| Hot chips | | | | | | | Yes 1 | No 2 |  |  |  |  |  |  |  |  |  |  |
| Biscuits/cakes/muffins | | | | | | | Yes 1 | No 2 |  |  |  |  |  |  |  |  |  |  |
| Sweets | | | | | | | Yes 1 | No 2 |  |  |  |  |  |  |  |  |  |  |
| Chocolates | | | | | | | Yes 1 | No 2 |  |  |  |  |  |  |  |  |  |  |
| Chips/crisps | | | | | | | Yes 1 | No 2 |  |  |  |  |  |  |  |  |  |  |
| Tea/coffee | | | | | | | Yes 1 | No 2 |  |  |  |  |  |  |  |  |  |  |
| Soft drinks | | | | | | | Yes 1 | No 2 |  |  |  |  |  |  |  |  |  |  |
| Juices | | | | | | | Yes 1 | No 2 |  |  |  |  |  |  |  |  |  |  |
| Water | | | | | | | Yes 1 | No 2 |  |  |  |  |  |  |  |  |  |  |
|  | | | | | | | | |  |  |  |  |  |  |  |  |  |  |
| Other: |  | | | | | | Yes 1 | No 2 |  |  |  |  |  |  |  |  |  |  |
|  | | | | | | | | |  |  |  |  |  |  |  |  |  |  |
|  | | | | | | | | |  |  |  |  |  |  |  |  |  |  |
| SECTION B: The vendor’s site | | | | | | | | |  |  | *Office* *use* | | | | | | | |
| 1. What does the street-food vendor site look like?  : | | | | | | | | |  |  |  |  |  |  |  |  |  |  |
| Does it have a roof cover? | | | | | | Yes 1 | | No 2 |  |  |  |  |  |  |  |  |  |  |
| Does it have walls? | | | | | | Yes 1 | | No 2 |  |  |  |  |  |  |  |  |  |  |
| Does it have a counter? | | | | | | Yes 1 | | No 2 |  |  |  |  |  |  |  |  |  |  |
| Is it a caravan? | | | | | | Yes 1 | | No 2 |  |  |  |  |  |  |  |  |  |  |
| Is food placed on the pavement? | | | | | | Yes 1 | | No 2 |  |  |  |  |  |  |  |  |  |  |
| Is it a kiosk with sides & roof? | | | | | | Yes 1 | | No 2 |  |  |  |  |  |  |  |  |  |  |
| Is it a shipping container? | | | | | | Yes 1 | | No 2 |  |  |  |  |  |  |  |  |  |  |
| Is it a temporary stall packed up after use? | | | | | | Yes 1 | | No 2 |  |  |  |  |  |  |  |  |  |  |
|  | | | | | | | | |  |  |  |  |  |  |  |  |  |  |
| Other: | Specify details | | | | |  | | |  |  |  |  |  |  |  |  |  |  |
|  | | | | | | | | |  |  |  |  |  |  |  |  |  |  |
|  | | | | | | | | |  |  |  |  |  |  |  |  |  |  |

| SECTION C: Hygiene status of vendors and site | | | |  |  | *Office* *use* | | | | | | | | |
| --- | --- | --- | --- | --- | --- | --- | --- | --- | --- | --- | --- | --- | --- | --- |
| 1. Hygiene status of vendors: | | | |  |  |  |  | |  |  |  |  |  |  |
| Short, clean nails? | | Yes 1 | No 2 |  |  |  |  | |  |  |  |  |  |  |
| Hands free of sores? | | Yes 1 | No 2 |  |  |  |  | |  |  |  |  |  |  |
| Smoking while working with food? | | Yes 1 | No 2 |  |  |  |  | |  |  |  |  |  |  |
| Jewellery/bangles on hands? | | Yes 1 | No 2 |  |  |  |  | |  |  |  |  |  |  |
| Handling money without washing hands in-between? | | Yes 1 | No 2 |  |  |  |  | |  |  |  |  |  |  |
| Vendor appears to have a cold/runny nose? | | Yes 1 | No 2 |  |  |  |  | |  |  |  |  |  |  |
|  | | | |  |  |  |  | |  |  |  |  |  |  |
| Other: |  |  | |  |  |  |  | |  |  |  |  |  |  |
|  | | | |  |  |  |  | |  |  |  |  |  |  |
| 2. Protective clothing worn by vendor: Full apron? | | Yes 1 | No 2 |  |  |  | |  | |  |  |  |  |  |
| Half apron? | | Yes 1 11 | No 2 |  |  |  | |  | |  |  |  |  |  |
| Overall? | | Yes1 | No 2 |  |  |  | |  | |  |  |  |  |  |
| Hair-covering? | | Yes1 | No 2 |  |  |  | |  | |  |  |  |  |  |
| Gloves? | | Yes 1 | No 2 |  |  |  | |  | |  |  |  |  |  |
| 3. Does apron/overall appear to be clean? | | Yes 1 | No 2 |  |  |  | |  | |  |  |  |  |  |
| 4. Is there a basin or tap for washing hands? | | Yes1 | No 2 |  |  |  | |  | |  |  |  |  |  |
| 5. Is there soap for washing hands? | | Yes 1 | No 2 |  |  |  | |  | |  |  |  |  |  |
| 6. Is there any antiseptic solution for washing? | | Yes 1 | No 2 |  |  |  | |  | |  |  |  |  |  |
| 7. Is there a cloth for drying hands? | | Yes 1 | No 2 |  |  |  | |  | |  |  |  |  |  |
| 8. Is there a clean washing cloth/sponge for washing utensils/food? | | Yes1 | No 2 |  |  |  | |  | |  |  |  |  |  |
| 9. Is there a clean drying cloth for utensils/food? | | Yes 1 | No 2 |  |  |  | |  | |  |  |  |  |  |
| 10. Any other comments about hygiene?................................................. | | | |  |  |  |  | |  |  |  |  | | |
| ………………………………………………………………………………………………… | | | |  |  |  |  | |  |  |  |  | | |
|  | | | |  |  |  |  | |  |  |  |  |  |  |
|  | | | |  |  |  |  | |  |  |  |  |  |  |
| The next questions are **ONLY if the vendor prepares food at the stall** | | | |  |  |  |  | |  |  |  |  | | |
|  | | | |  |  |  |  | |  |  |  |  |  |  |
| 11. Surface on which food is prepared? | |  | |  |  |  |  | |  |  |  |  |  |  |
| Plastic | | Yes 1 | No 2 |  |  |  |  | |  |  |  |  |  |  |
| Wood | | Yes 1 | No |  |  |  |  | |  |  |  |  |  |  |
| Metal | | Yes 1 | No |  |  |  |  | |  |  |  |  |  |  |
| Cement | | Yes 1 | No |  |  |  |  | |  |  |  |  |  |  |
| Cardboard/newspaper | | Yes 1 | No |  |  |  |  | |  |  |  |  |  |  |
| Glass | | Yes 1 | No |  |  |  |  | |  |  |  |  |  |  |
| Cloth | | Yes 1 | No |  |  |  |  | |  |  |  |  |  |  |
|  | | | |  |  |  |  | |  |  |  |  |  |  |
| The next questions are **ONLY if the vendor prepares food at the stall (**CONT**)** | | | |  |  |  |  | |  |  |  |  | | |
|  | | | |  |  |  |  | |  |  |  |  |  |  |
| 12. Does the vendor use separate utensils for cooked and raw food? | | Yes 1 | No 2 |  |  |  |  | |  |  |  |  |  |  |
| 13. Does there appear to be adequate take away containers? | | Yes 1 | No 2 |  |  |  |  | |  |  |  |  |  |  |
| 14. Does there appear to be adequate cutlery? | | Yes 1 | No 2 |  |  |  |  | |  |  |  |  |  |  |
| 15. Does the cutlery appear to be clean?  1  18. | | Yes 1 | No 2 |  |  |  |  | |  |  |  |  |  |  |
| 16. Does the vendor use his hands use gloves? | | Yes 1 | No 2 |  |  |  |  | |  |  |  |  |  |  |
| 17. Does the vendor use cutlery to pick up food? | | Yes 1 | No 2 |  |  |  |  | |  |  |  |  |  |  |
| 18. Is cooked food kept covered? | | Yes1 | No 2 |  |  |  |  | |  |  |  |  |  |  |
| 19. Is cooked food kept warm? | | Yes1 | No 2 |  |  |  |  | |  |  |  |  |  |  |
| 20. If yes, how?................................................... | | | | Yes1 | No 2 |  |  | |  |  |  |  |  |  |
