# Supplementary material for: The development of an evidence-based street food vending model within a socioecological framework: A guide for African countries
Source: PLoS One. 2019 Oct 22;14(10):e0223535. doi: 10.1371/journal.pone.0223535 (PMC6804966; doi:10.1371/journal.pone.0223535)
Supplement: S1 Supporting information — (DOCX) [file pone.0223535.s004.docx]

Street Food Interview Schedule

**Municipal Officers**

- Thank you for agreeing to this interview. Your time is most appreciated.

As you know the overall aims of the Street Food Project is to:

i) To evaluate the nutritional contribution of street food to the diet of the population of the Western Cape (urban areas, townships and informal settlements) and **to develop an operational model for selling healthy street foods.**

ii) To understand and evaluate existing models (options) for providing street food which enables people to eat more fruit and vegetables, provides a sustainable income for the vendors, while maintaining optimal food safety.

- I would specifically like to talk to you about the proposed SFVBM
- Especially with regards to the licensing and certification and all procedures involved there.
- But first I would like to present to you a list of challenges SF vendors identified during their questionnaire interviews and get your response to it:

Vendors face an array of challenges on a daily basis, making it very difficult for them to keep their businesses afloat. Vendors were asked three open ended questions in which they were asked to express what they would change about the vending operation, the problems they experienced running their vending operation and if there is anything the municipality could do to help improve their business.

1. The most vendors (n=573) mentioned the **lack of facilities i.e. access to electricity, water, toilets etc**. as a major challenge in their operation.
2. The **need for permanent structure** (n=512) and **shelter and storage** (n=180) came through very strong which would address the **challenge vendors face with weather conditions** (n=263) as well as the **problem of building and dismantling of stall** (n=29) daily.
3. Vendors reported incidents **with law enforcement/securities as a major challenge** (n=186), those trading in or on train stations also reported having issues with Metrorail securities (n=37).
4. **Crime and theft** (n=222) were a big issue vendors had to contend with and expressed **the need for improved security or policing** (n=57).
5. **The need of permits** (n=186) were expressed, along with **some issues surrounding permits** (n=30) were expressed.

- **Can I get a few comments and your views about these issues as I mention them?**

1. The existing policies relating to Street Foods…

- The Foodstuffs, Cosmetics and Disinfectants Act 54 of 1972.
- The Health Act 63 of 1977.
- The International Health Regulations Act 28 of 1974
- The Agricultural Product Standards Act 119 of 1990
- The Liquor Products Act 60 of 1989
- The Abattoir Hygiene Act 121 of 1992
- The Animal Diseases Act 35 of 1984
- The Fertilizers, Farm Feeds, Agricultural Remedies and Stock Remedies Act 36 of 1947
- The Medicines and Related Substances Act 101 of 1965
- The Standards Act 29 of 1993
- The Plant Breeders Rights Act 15 of 1976
- The Agricultural Pest Act 36 of 1983
- The Trade Metrology Act 77 of 1973 and the Trade Marks Act 62 of 1963 both relate to food labelling.
- **What are the bylaws of the CCT?** By-laws of local authorities. Many local authorities have food hygiene by-laws which they enforce in addition to the national regulations.

1. A big portion of our vendor sample did not have any form of certification. Please tell me about your application process and its requirements.

- *How often do applications get turned down?*
- *What is the waiting period?*
- *What are the financial implications?*
- *How long is a license/certificate valid?*
- *What is the difference between a license and certificate?*
- *Are there any other forms of permitting/authorising documentation a vendor could have?*
- *Is there something like a temporary permit? And how does it work?*
- *What are the key factors in being granted a certificate/license?*

1. Do vendors require a business plan?

- *If so, what should it entail?*
- *Is there any institution or organisation vendors can specifically look to for financial assistance?*

1. What are the hygiene requirements for street food vendors specifically?

- *Water? Electricity requirements?*
- *Is there any specific guide that you adhere to pertaining to safety?*
- *Do you provide any training in this regard?*

1. Is there any support available for vendors from the municipality or local/provincial government?

- *If so, do they know it is available and who to contact?*

1. Does the city have a vision for street food vendors?

- *If so, what is the vision?*
- *Would the aims of the SFVBM fit in with this vision?*

1. **TCP project:** What was CPT’s involvement? Were any street food vendors reached?

- Permissions to use their materials?
