# Supplementary material for: The development of an evidence-based street food vending model within a socioecological framework: A guide for African countries
Source: PLoS One. 2019 Oct 22;14(10):e0223535. doi: 10.1371/journal.pone.0223535 (PMC6804966; doi:10.1371/journal.pone.0223535)
Supplement: S3 Data — (RTF) [file pone.0223535.s007.rtf]

Codes-quotations list
Code-Filter: All
______________________________________________________________________

HU:	SF - EHP - ED - CCT
File:	 [C:\Users\jhill\Documents\Scientific Software\ATLASti\TextBank\SF - EHP - ED - CCT.hpr7]
Edited by:	Super
Date/Time:	2015-09-02 11:16:57
______________________________________________________________________

Code: 3 sets of 5 keys {1-0}

P 1: Interview with XXXXX.docx - 1:9 [I think there are altogether t..]  (42:42)   (Super)
Codes:	[3 sets of 5 keys] 
No memos

 I think there are altogether three sets of 5 keys, of key things if you know what I mean. I can't remember what the other one is. 

______________________________________________________________________

Code: 5 keys to nutrition {1-0}

P 1: Interview with XXXXX.docx - 1:8 [Ja and then they brought it do..]  (40:40)   (Super)
Codes:	[5 keys to nutrition] 
No memos

Ja and then they brought it down. But they also have 5 keys to nutrition and 5 keys to this, there is a couple of…

______________________________________________________________________

Code: 5 keys to safer foods {7-0}

P 1: Interview with XXXXX.docx - 1:7 [no, absolutely. Actually inter..]  (38:38)   (Super)
Codes:	[5 keys to safer foods] 
No memos

 no, absolutely. Actually interesting to note; the five safer food was developed for Africa and the re-, for every region they sort of took certain things under consideration. So this is really something that has been researched for SA. This particular one that we use. 

P 1: Interview with XXXXX.docx - 1:49 [Food safety. 5 keys of safety,..]  (107:107)   (Super)
Codes:	[5 keys to safer foods] [COA_requirements] 
No memos

 Food safety. 5 keys of safety, they are quite happy as far as that is concerned. Food covered refrigerated, temperature control. Temperature control, very important for them, absolutely important. 

P 1: Interview with XXXXX.docx - 1:52 [Training in this regard. We do..]  (111:111)   (Super)
Codes:	[5 keys to safer foods] 
No memos

Training in this regard. We don't really but we try and get to the hawkers through the 5 keys to safer food initiative. I know in Khayelitsha they have a nice thing going with the hawkers, they get then in from time to time, and actually train them and things like that. But it is not actually a pre-requisite for us. 

P 1: Interview with XXXXX.docx - 1:53 [They have, I think they have a..]  (113:113)   (Super)
Codes:	[5 keys to safer foods] 
No memos

They have, I think they have a whole session with them and things like that. But they do give the 5 keys to safer food pamphlet if they have it. 

P 2: Meeting EHManagers.doc - 2:42 [For instance the five keys the..]  (75:75)   (Super)
Codes:	[5 keys to safer foods] 
No memos

For instance the five keys the five keys most of them we have done quite a few of them.

P 2: Meeting EHManagers.doc - 2:49 [But, please I don't know I wou..]  (104:104)   (Super)
Codes:	[5 keys to safer foods] [R962] [R962 diagram] 
No memos

 But, please I don't know I would not want you to leave it out now that we've got this ideal cut work space, how the work space should like and then we've got the five keys let us also have him or her to say that this is how you as a food vendor should look like so that we've got a full picture. And then probably also if in this cart you could also maybe have the space where they could maybe later [unclear 0:40:25] something there where they would – their name could be so that even if somebody a customer comes then they buy something then he or she gets food poisoning then she can quickly tell us that she got it from Zandile Sparsa shop you work from Zandile's braai stand and then you know that there is only one Zandile braai stand there and that would also make us fully compliant in terms of R962 because those are the type of other things that are needed.

P 2: Meeting EHManagers.doc - 2:51 [we use five keys that is one o..]  (111:111)   (Super)
Codes:	[5 keys to safer foods] 
No memos

we use five keys that is one of the things that is everybody in the city we adopt world organization five keys that is what we preach. 

______________________________________________________________________

Code: 5 keys to safer foods_training {1-0}

P 2: Meeting EHManagers.doc - 2:52 [I will give you an example we ..]  (111:111)   (Super)
Codes:	[5 keys to safer foods_training] 
No memos

 I will give you an example we had a project with funding that we had that we for example used EPWP Workers it's an expanded public work's program that we use then and we had funding where we would as a starter pack, you would get the traders together have a workshop five keys whatever with the requirements of the city, legislation and you have the starter pack so we would give him like 500 milliliter or 250 milliliter dishwasher a table those plastic table cloth, small jik and an apron and a little cap. So that would be a starter kit so if you have gone through the program then this is what you get plus you get your not the COA but a certificate so we would rather say a Certificate of Attendance Act city of Cape Town Food Vendors Workshop.

______________________________________________________________________

Code: Allocated vs non-allocated zones {4-0}

P 2: Meeting EHManagers.doc - 2:30 [I will give you an example the..]  (55:55)   (Super)
Codes:	[Allocated vs non-allocated zones] 
No memos

I will give you an example there are certain areas in I'm talking about my area now – certain areas you will be allowed Delft for example there is not allocated space. So maybe ride through the road and stand at a certain place but the moment you go to for example Bellville CBD there are allocated zones where people are allocated areas where people can and that is a permit system in order words you have to apply for it you have to pay for it and they have certain rules.

P 3: Meeting_XXXXX.doc - 3:52 [Now at the moment, if you were..]  (98:98)   (Super)
Codes:	[Allocated vs non-allocated zones] [Permit] 
No memos

Now at the moment, if you were to look at the Cape Town municipal area roughly 60% of the entire municipal area is an area where you need a location permit so take a silly example which Mitchell's plain town centre if you want to take there you need to get permission to trade in that yellow block and if you don't have a permit you can't trade so that is the one side of it. And by the way, the other 40% is effectively area where we don't need a location permit so as long as you comply with bylaws you can go and stand. So again using a silly example if you are AZ Berman Drive of Mitchells Plan you can stand alongside the road you don't need permission.

P 3: Meeting_XXXXX.doc - 3:90 [ou see there is a tension. let..]  (167:167)   (Super)
Codes:	[Allocated vs non-allocated zones] [Concession letters] [Vendor challenges] 
No memos

ou see there is a tension. let's just talk first of all about the 40% on municipal area that you don't need a location permit and just so that you can get a picture of it at the moment the Blouberg that whole area up the coast the whole of Khayelitsha there two pockets of Khayelitsha, but most of Khayelitsha town centre and then the area from Khayelitsha heading across towards Durbanville so that area is effectively a free trading area excluding the townships and the mentality of the community onto the politicians the ward councilor is that every single key informal trader is illegal so law enforcement will go there and the first thing is where is your permit. The fact that they talk to my staff and you don't need a permit the law says you don't need a permit but the mentality of law enforcement and the South African Police where's your permit and the trader gets very confused, I don't need a permit, but. So what we do and I'm only talking about the 40% of the area we issue what we call concession letters, its got no legal status it simply says you can trade these are the basic conditions and it is a piece of paper that the trader can get. Now, the councilors are very unhappy that we are issuing that letter and I have been challenged all over the place on it but I have succeeded with the challenge…

P 3: Meeting_XXXXX.doc - 3:92 [The other challenge funny enou..]  (171:171)   (Super)
Codes:	[Allocated vs non-allocated zones] 
No memos

The other challenge funny enough I've got a meeting this afternoon on it, it has to do with the fish traders now remember fish trade is they pick up the fish from the boats, put it on their bakkie and then they go and sell on the roadside now again most of that is within the no permit no trade areas, but yet the demand is there people want to buy.

______________________________________________________________________

Code: Any improvement welcome {1-0}

P 4: SF Workshop_transcript.docx - 4:17 [So anything would be an improv..]  (26:26)   (Super)
Codes:	[Any improvement welcome] 
No memos

So anything would be an improvement from my side. So something like this I would welcome it. 

______________________________________________________________________

Code: Applicable legislation {5-0}

P 1: Interview with XXXXX.docx - 1:15 [This one the existing legislat..]  (64:64)   (Super)
Codes:	[Applicable legislation] 
No memos

 This one the existing legislation, the foodstuffs and cosmetics and disinfectants act is definitely applicable. 

P 1: Interview with XXXXX.docx - 1:16 [Because the certificate of acc..]  (64:64)   (Super)
Codes:	[Applicable legislation] 
No memos

Because the certificate of acceptability is a regulation and has been promulgated under the food stuffs act, ok. The health act will probably also be applicable but to a lesser extent. 

P 1: Interview with XXXXX.docx - 1:20 [What is going to be important ..]  (71:71)   (Super)
Codes:	[Applicable legislation] 
No memos

What is going to be important for them to understand is the meat safety act. So all meat that is slaughtered, gets inspected and these are the kinds of things they need to look out for. So a carcass that has been inspected and marked and things like that. 

P 1: Interview with XXXXX.docx - 1:23 [What is applicable is if they ..]  (71:71)   (Super)
Codes:	[Applicable legislation] 
No memos

What is applicable is if they were to sell fruit and vegetables on a stand, and if we were to take samples, those residues on the product, we will test and that is in terms of the health legislation, ok. Although the use of that stuff is guided by this particular act, that is a bit different. 

P 1: Interview with XXXXX.docx - 1:24 [Medicines and related substanc..]  (71:71)   (Super)
Codes:	[Applicable legislation] 
No memos

Medicines and related substances act (silence…sigh and quint laugh)…I don't know, sometimes these people have these herbal remedies and, so lets leave that. 

______________________________________________________________________

Code: Application process {7-0}

P 1: Interview with XXXXX.docx - 1:36 [I don't think we turn down a l..]  (90:90)   (Super)
Codes:	[Application process] 
No memos

I don't think we turn down a lot, but that is very difficult to say. You know that would be very difficult to find. 

P 3: Meeting_XXXXX.doc - 3:57 [or the location permit you fil..]  (104:104)   (Super)
Codes:	[Application process] 
No memos

or the location permit you fill in an application form and then we as the city we've got a screening process so key to that are you an existing business person or are you an unemployed person we lean toward unemployed people more than business people. And then are you in South Africa legally so if you are foreign national, do you have a work certificate or work permit, if you are a South African citizen, ID and then there is the basic screening because our focus is on unemployed people. S

P 3: Meeting_XXXXX.doc - 3:58 [So we ask a number of question..]  (104:104)   (Super)
Codes:	[Application process] 
No memos

 So we ask a number of questions and they the questions so do you employ more than 20 people – yes so obviously an informal trader, no so… And then we have an approval process within the city but then once a permit is issued to a vendor or a street trader that permit doesn't have a lifespan to it. 

P 3: Meeting_XXXXX.doc - 3:65 [You will come to one of my sta..]  (115:115)   (Super)
Codes:	[Application process] 
No memos

You will come to one of my staff they at eight districts you say I'm selling fruit and vegetables I would like to trade with this location my staff member will say okay is that a trading plan area or non trading plan area. If it is a trading plan area is there a demarcated bay yes or no, and if yes, is it vacant. And if it is vacant fill in the form comply with the criteria we get the approval you then go to cash office, you pay your R80 and you get a permit and then that permit will operate on a month to month.

P 3: Meeting_XXXXX.doc - 3:95 [It is a valid comment we've un..]  (178:178)   (Super)
Codes:	[Application process] 
No memos

It is a valid comment we've undergone as a city undergone a process change initially it was literally a case of I'm just going to do Bellville as an example a trader wants to trade in Bellville they will go and see the area coordinator, fill in an application form, she will make sure that there is compliance and then she will issue the permit and you go to the cash office and pay for it. so it was literally as quick as that.

P 3: Meeting_XXXXX.doc - 3:96 [There was a concern that it ma..]  (178:178)   (Super)
Codes:	[Application process] 
No memos

There was a concern that it may the area coordinator is vulnerable in terms of favoritism so now there has been the separation, she would fill in the application form check the compliance as it were but she would make a recommendation to me and I need to make sure that she has complied that she followed the right policies guides. And then I would then sign it off and it goes through to my director he will then authorize it and then the information will come back where before he literally would walk in and walk out with the permit the delay now, best case scenario about two to three weeks. 

P 3: Meeting_XXXXX.doc - 3:98 [The city policy on communicati..]  (180:180)   (Super)
Codes:	[Application process] [Vendor challenges] 
No memos

The city policy on communication that you need to communicate, you need to advise, you need to inform you shouldn't wait for more than seven days without some form of communication but I know the reality it's at least true. And then also, I think traders are opportunistic I had a lady phone me up and she said she applied for a permit eight years ago and she hasn't heard anything and my response to her I said sorry, I just struggle eight years is a long time, but 48 months ago surely when you apply and you haven't heard in a month or two months time you then… And if you still not getting an answer then talk to the senior escalator, eight yes no something is not right.

______________________________________________________________________

Code: Banting {1-0}

P 2: Meeting EHManagers.doc - 2:91 [A similar tradition and now wi..]  (229:229)   (Super)
Codes:	[Banting] 
No memos

A similar tradition and now with banting as well you – the banting thing is another thing is that you have to consider because I mean Tim has now put the span in the works with…

______________________________________________________________________

Code: Business Act 1991 {3-0}

P 3: Meeting_XXXXX.doc - 3:4 [Maybe just to talk about the l..]  (25:25)   (Super)
Codes:	[Business Act 1991] 
No memos

Maybe just to talk about the legislative framework around street trading and that essentially started in 1991 where parliament at that stage scrapped all legislation governing informal trading and street trading and that was the Business Act of 1991. 

P 3: Meeting_XXXXX.doc - 3:8 [Now, one of the challenges tha..]  (31:31)   (Super)
Codes:	[Business Act 1991] [Bylaws of the city] 
No memos

Now, one of the challenges that municipalities have is that the 1991 Business Act is very prescriptive in what you can or can't do as a city and at one stage the city of Cape Town went to seek legal opinion on are we still bound by the 1991 Business Act or can we formulate our own policies and bylaws. And the response was that first of all the 1991 legislation is I used the word I have never seen before is it's good law but it acknowledges that it is a 1991 legislation so it is outdated in terms of the constitution of South Africa, the structures of the systems and things have changed. And so what the senior council said to us, he said maybe this was city of Cape Town specific you can draft your own bylaw and obviously your own policy and the powers we do that come from the constitution and it can be at variants to the 1991 Business Act. 

P 3: Meeting_XXXXX.doc - 3:14 [Okay one might debate selling ..]  (37:37)   (Super)
Codes:	[Business Act 1991] 
No memos

Okay one might debate selling hot dogs, fatty food but that's not also necessary a bad thing, but having a trader selling cigarettes outside of schools… Now, the 91 legislation says that you are not allowed to be prescriptive on which sales so as long as you are selling legal goods the city is not allowed to be prescriptive in terms of 91 legislation. Our councilors and they have to use the cigarette selling as over stated but they don't agree they said no, as a city we should be able to be prescriptive of what is sold and not sold, and as a city official, I in my mind haven't drawn that line yet. I absolutely understand cigarettes and school children but to me where do you start drawing the line, what about somebody selling hamburgers where you fry it in oil that is also not healthy.

______________________________________________________________________

Code: Business license {12-0}

P 1: Interview with XXXXX.docx - 1:12 [Now I understand and then of c..]  (48:48)   (Super)
Codes:	[Business license] 
No memos

Now I understand and then of course the business licensing and things like that. 

P 1: Interview with XXXXX.docx - 1:29 [Now what happens when people a..]  (85:85)   (Super)
Codes:	[Business license] 
No memos

Now what happens when people apply for their license, they apply for their license, they apply for a hawker and meals license, they basically pay their R10. 

P 1: Interview with XXXXX.docx - 1:30 [Yes. Since 1991 R10. It is mor..]  (87:87)   (Super)
Codes:	[Business license] 
No memos

Yes. Since 1991 R10. It is more of a nuisance factor than anything else. Formal premises is R25.but the idea is to have them on our books so we know where they are, ok. Okay, they apply for their license, they go to the environmental health office, they apply for their license, they pay the R10. For hawkers it gets referred to the environmental health practitioner, and if there is gas on the stall it goes to the department of fire and safety as well. For the gas issue. Everyone is happy; they get issued with their license. 

P 1: Interview with XXXXX.docx - 1:32 [Now also remember that when we..]  (87:87)   (Super)
Codes:	[Business license] 
No memos

Now also remember that when we look at these things we do the license, we actually look at the home premises as the basis. This is where the people takes their carts to clean at night and store their things and things like that. So we look at the base premises alright, and that, that is the important part of it.

P 1: Interview with XXXXX.docx - 1:38 [Financial implications, as I s..]  (92:92)   (Super)
Codes:	[Business license] 
No memos

Financial implications, as I said R10. 

P 1: Interview with XXXXX.docx - 1:39 [How long is the license valid?..]  (93:93)   (Super)
Codes:	[Business license] 
No memos

How long is the license valid? Until the person sells the business. And the certificate as well. And there …

P 1: Interview with XXXXX.docx - 1:40 [That R10 is forever. If you se..]  (95:95)   (Super)
Codes:	[Business license] 
No memos

That R10 is forever. If you sell your business. 

P 2: Meeting EHManagers.doc - 2:29 [he other thing that I will sen..]  (55:55)   (Super)
Codes:	[Business license] [Permit] 
No memos

he other thing that I will send you is the Business License Act so those are the two pieces of legislation that we would look at. Johan has said that besides even if you have both of those legislations or you… Even if you have the certificate and the business license it still doesn't allow you to trade in certain areas because that is where the permit system comes in so if you have – 

P 2: Meeting EHManagers.doc - 2:35 [But, however, you've got to co..]  (61:61)   (Super)
Codes:	[Business license] [Certificate of acceptability] 
No memos

But, however, you've got to comply it doesn't matter where you stand whether it is private or whether it's on the open you still got to have both those pieces of paper, the business license dependent like for example the business license the COA will sell. The COAs is low cost the business license has got a ridiculous cost of R10.

P 2: Meeting EHManagers.doc - 2:36 [A once off thing which is 1991..]  (65:65)   (Super)
Codes:	[Business license] 
No memos

A once off thing which is 1991 piece of legislation it's R25 for example R25 that's a business license I think they looking at.

P 3: Meeting_XXXXX.doc - 3:53 [But then the second side of it..]  (98:98)   (Super)
Codes:	[Business license] [Certificate of acceptability] 
No memos

But then the second side of it, it comes down to and this is more Christa's area has to deal with depending on the nature of the food that you are selling determines what permission you need. So you need a Certificate of Acceptability and you need business license those are the two key ones so if you are cooking food for example you need both those certificates.

P 3: Meeting_XXXXX.doc - 3:54 [It's a Certificate of Acceptab..]  (100:100)   (Super)
Codes:	[Business license] [Certificate of acceptability] [Confusion_licensing/permits] 
No memos

It's a Certificate of Acceptability and a business license. Now, I do need to say that those two bits of document cause confusion with the vendor because the vendor in his mind comes to the municipality and says I want to sell boerewors okay this is what you need and they go to the health department they gets those two bits pieces of paper and then they go down to Adderley Street and there is not trading and next thing law enforcement hits them hard, then I say but I got them… This just gives you permission from a health point of view to sell it doesn't give you a right to go onto private property and sell, it doesn't give you a right to go anywhere so you need the location permit. But, in the street vendors mind there is a lot of… [speaking simultaneously]

______________________________________________________________________

Code: Business license_homebase accepted as premises {1-0}

P 1: Interview with XXXXX.docx - 1:57 [Of course it would be, but the..]  (122:122)   (Super)
Codes:	[Business license_homebase accepted as premises] [Vendor challenges] 
No memos

Of course it would be, but then we use the home premises as a base. So we accept that for a base. If it were to be close by t their stalls the people would look into it, but that would not be the main stumbling block for us not to give somebody a license. For a hawkers license we are happy with the home base premises. 

______________________________________________________________________

Code: Business marketing {3-0}

P 2: Meeting EHManagers.doc - 2:53 [The second thing is you've got..]  (113:113)   (Super)
Codes:	[Business marketing] 
No memos

The second thing is you've got to introduce certain business principles as well which will maybe not our co-function[?] but which will influence maybe the outcome that will help us with the product that is sold. For example, you would be looking at Zandile spoke about it the appearance of the person, how you present yourself to the customer, your surfaces how do you present if you have an open like a cart – the cart is completely open right around so people would be able to see from either side depending where you stand to see how you prepare your food so in other words what you need to have is you need to have your clean water, you need to have your clean rags or whatever you have, your nails those things. So those are the corporate image of your little business that you take on so somebody has got to look at business principles 

P 2: Meeting EHManagers.doc - 2:54 [but connected to as well becau..]  (113:113)   (Super)
Codes:	[Business marketing] 
No memos

but connected to as well because what we also do is like she says now, part of telling your community or your people I'm safe I've got my COA put my thing there whatever it counts in my favor also to say I have been certified by the city, and then also the safety of your product as well. What are the principles and that is where five keys come in – what are the principles to keep your products safe. 

P 2: Meeting EHManagers.doc - 2:56 [That's what we always remind t..]  (115:115)   (Super)
Codes:	[Business marketing] 
No memos

That's what we always remind them when we give them those workshops that you must try and invest in your business and in that way in you trying to comply with our health regulations and requirements you are also investing in your own business that means that you will be attracting more customers to come to you nobody would want to come and buy from someone who is very dirty, this person just woke up and decided to go to a shop we always try and make them see things from that broader perspective.

______________________________________________________________________

Code: Bylaws of the city {9-0}

P 1: Interview with XXXXX.docx - 1:27 [Urhm, then basically you can j..]  (73:73)   (Super)
Codes:	[Bylaws of the city] 
No memos

Urhm, then basically you can just the bylaws of the city, we have bylaws and some of them are supplementing legislation, the national legislation, so any bylaws from the towns and things they need to be aware of that. Because those things go around nuisances, and so on. So if there is a nuisance and they do not clean up after them and things like that, there may be other bylaws not just environmental health but also solid waste and things like that. 

P 1: Interview with XXXXX.docx - 1:28 [Look in fact the thing is, the..]  (79:79)   (Super)
Codes:	[Bylaws of the city] 
No memos

Look in fact the thing is, the city, I don't understand the city's bylaw itself, you know. Because solid waste has a bylaw. Paul and them have a trading bylaw. Urhm, you have bylaws on fire and safety and things like that. That we do not deal with, and I am not even aware of some of the bylaws in there. I know our own specific bylaw. And that as I say deals with nuisances, and so on. So, I am going to see what I can do but I am not going to promise that it is going to be a comprehensive list. 

P 3: Meeting_XXXXX.doc - 3:1 [To the informal trading and th..]  (7:7)   (Super)
Codes:	[Bylaws of the city] 
No memos

To the informal trading and therefore the economy.

P 3: Meeting_XXXXX.doc - 3:8 [Now, one of the challenges tha..]  (31:31)   (Super)
Codes:	[Business Act 1991] [Bylaws of the city] 
No memos

Now, one of the challenges that municipalities have is that the 1991 Business Act is very prescriptive in what you can or can't do as a city and at one stage the city of Cape Town went to seek legal opinion on are we still bound by the 1991 Business Act or can we formulate our own policies and bylaws. And the response was that first of all the 1991 legislation is I used the word I have never seen before is it's good law but it acknowledges that it is a 1991 legislation so it is outdated in terms of the constitution of South Africa, the structures of the systems and things have changed. And so what the senior council said to us, he said maybe this was city of Cape Town specific you can draft your own bylaw and obviously your own policy and the powers we do that come from the constitution and it can be at variants to the 1991 Business Act. 

P 3: Meeting_XXXXX.doc - 3:9 [as a municipality the constitu..]  (31:31)   (Super)
Codes:	[Bylaws of the city] 
No memos

as a municipality the constitution and the structures at an assistants act allows you to make bylaw and policies within your areas of competencies and street trading it is interesting they use the word street trading not informal trading, it's one of those competencies. So that gave rise to us to draft the policy and then from that we drafted our own bylaw.

P 3: Meeting_XXXXX.doc - 3:10 [Let me finish talking about… T..]  (35:35)   (Super)
Codes:	[Bylaws of the city] [Policies vs legislation] [Various departments involved] 
No memos

Let me finish talking about… The city has got a policy its got its bylaw and what we did in terms of both the document took very much into account the 1991 document, so the backbone to our policies and bylaws and then also took into account other legislations around for example food trading. So when it comes to environmental health aspects and more specifically street trading and food, we don't specify in our bylaw just to say that any other bylaw that exists or any other legislation you need to comply with it and we had a huge debate in one sense and if you think from an informal trader perspective they don't want to have to go and search the entire legislative framework to find out what is applicable to them. And then the city we don't want to necessarily take one document and pull in from every other so you got environmental health legislation, you've got this legislation to do with solid waste your cleansing, you got traffic bylaws and all that that impacts on informal economy because then when you change your environmental health legislation you've got to remember you've got to change your informal trading bylaws so it was a debate and I don't think we settled that finally so we have said, Christa will talk about all the environmental health aspects and that embodied in their policies and their legislation. In the city's policy and bylaws we have just made reference to that you need to be aware of health issues and health and sanitation we don't specify…

P 2: Meeting EHManagers.doc - 2:21 [But if it can mean that I mean..]  (44:44)   (Super)
Codes:	[Bylaws of the city] 
No memos

But if it can mean that I mean those bylaws were passed by the city those policies were passed by the city if it means that city would need to change the bylaws to accommodate those wheeling trolleys let it be because it is an investment to the health of the very same citizens that we claim to be servicing.  

P 2: Meeting EHManagers.doc - 2:25 [the air pollution bylaw, the e..]  (49:49)   (Super)
Codes:	[Bylaws of the city] 
No memos

the air pollution  bylaw, the equality bylaw are being contravened by all the food vendors of the city they are using wood according to equality bylaw none of them we should really be fining them we should be getting rid of all of them if they are using wood. So if maybe we could maybe one could invent this piece of equipment that is then going to be working towards helping us meeting that goal of reducing the emissions and whatever and helping us to comply then maybe it is something that they would want to support.

P 3: Meeting_XXXXX.doc - 3:7 [And then the city has got its ..]  (31:31)   (Super)
Codes:	[Bylaws of the city] 
No memos

And then the city has got its policy and from that we formulated a bylaw. 

______________________________________________________________________

Code: Cape Town Partnership {3-0}

P 3: Meeting_XXXXX.doc - 3:46 [What I will also do is I am ju..]  (85:85)   (Super)
Codes:	[Cape Town Partnership] 
No memos

What I will also do is I am just taking a gap here I will give you the names of two people within the Cape Town partnership. Now, the Cape Town partnership is a non profitable organization which focuses mainly on the Cape Town CBD to promote and develop the Cape Town CBD but Bulelwa[?] who is the CEO of the Cape Town partnership she has two very strong passions the one is healthy food,

P 3: Meeting_XXXXX.doc - 3:47 [The reason I mentioned her and..]  (85:85)   (Super)
Codes:	[Cape Town Partnership] 
No memos

The reason I mentioned her and then Andrew I think it is Andrew Fleming one of the researchers in the Cape Town partnership they doing a lot of work with CPUT on the informal economy it's in the context of Cape Town CBD but it's talking about healthy food mobile vendor units, alternative lifestyle and that sort of thing. 

P 4: SF Workshop_transcript.docx - 4:25 [he city – Cape Town Partnershi..]  (51:51)   (Super)
Codes:	[Cape Town Partnership] 
No memos

he city – Cape Town Partnership is also highly facilitated with informal traders, they just happen to use the informal traders in the CBD area because it is convenient. But the idea is that they have been talking to traders about what works and what doesn't work. So maybe part of this exercise is to tap into that, whoever the people are there. 

______________________________________________________________________

Code: Cart requirements {15-0}

P 1: Interview with XXXXX.docx - 1:5 [Ja, but you also have to get t..]  (32:32)   (Super)
Codes:	[Cart requirements] 
No memos

Ja, but you also have to get the environmental health people involved in this. Because we have seen some of these carts and although they look very good on paper and their design. They have definite shortcomings in them. 

P 2: Meeting EHManagers.doc - 2:1 [Maybe what I could say is rega..]  (4:4)   (Super)
Codes:	[Cart requirements] 
No memos

Maybe what I could say is regarding the cart it is a very good idea but I'm just looking at the financing. Have you maybe looked at can the people afford this that is the

P 2: Meeting EHManagers.doc - 2:6 [But, even if you've got a sola..]  (19:19)   (Super)
Codes:	[Cart requirements] 
No memos

But, even if you've got a solar thing you've got to have the backup thing with the gas or something for when there is no sun and stuff like that and that safety aspect the gas is always the size of the bottles and stuff.

P 2: Meeting EHManagers.doc - 2:7 [I think the main thing is that..]  (21:21)   (Super)
Codes:	[Cart requirements] 
No memos

I think the main thing is that gas thing and stuff like a cooling facility the most important ones are usually your gas or whatever your preparation things what you use and then your storage facilities, your cool bags or cool facility, and something to wash hands I think that is the main aspects of the preparation thing that cold chain stuff that cooling system and your cleaning system.

P 2: Meeting EHManagers.doc - 2:8 [You see what the people usuall..]  (23:23)   (Super)
Codes:	[Cart requirements] 
No memos

You see what the people usually do they put in something like a little washing plastic basin thing like they do in caravans with a little 25 litre or 20 litre plastic can draining it down and one at the bottom where your waste water is going in which you can empty tonight because that is usually what they do with stuff like that.

P 2: Meeting EHManagers.doc - 2:15 [I think the cart of Cape Winel..]  (44:44)   (Super)
Codes:	[Cart requirements] [Cart_example] [R962] 
No memos

I think the cart of Cape Winelands will bring with it some answers to what you are looking for because they have a safe component in it there is I can't remember if it is a tap or 25 litre water container – clean water thing in whereby one can use the sink maybe to wash maybe your vegetables or whatever or the meat or anything and then at the bottom there is also an empty 25 litre container to drain the thing actually. So it's a typical – an ideal model that really satisfies us as EHPs because its got all the components of what we are looking for and then I think it also has a space for them to label their stalls because our regulations are R962 requires that if it is shop that you are selling then it must be clearly labeled with your name and everything.

P 2: Meeting EHManagers.doc - 2:26 [So if maybe we could maybe one..]  (49:49)   (Super)
Codes:	[Cart requirements] 
No memos

So if maybe we could maybe one could invent this piece of equipment that is then going to be working towards helping us meeting that goal of reducing the emissions and whatever and helping us to comply then maybe it is something that they would want to support.

P 2: Meeting EHManagers.doc - 2:45 [please forgive me is someone h..]  (94:94)   (Super)
Codes:	[Cart requirements] 
No memos

please forgive me is someone has already suggested it but if you can also have a cart that would allow for the washing of hands not just for the cleaning of the utensils and stuff just to prevent that that cross combination that we have a compartment that would be considered for the washing of hands and everything because I know that one is not there on that Winelands farm the cart.

P 2: Meeting EHManagers.doc - 2:80 [Did we include waste managemen..]  (196:196)   (Super)
Codes:	[Cart requirements] [Waste management] 
No memos

Did we include waste management?

P 2: Meeting EHManagers.doc - 2:81 [It's a big issue for us especi..]  (199:199)   (Super)
Codes:	[Cart requirements] [Waste management] 
No memos

It's a big issue for us especially what do you do with waste so you've got to try and build in some… A waste minimisation strategy with your thing because people don't like to take the waste home.

P 3: Meeting_XXXXX.doc - 3:48 [Because to me, the units are f..]  (85:85)   (Super)
Codes:	[Cart requirements] [Cart_challenges] 
No memos

Because to me, the units are fantastic on one side because if you've got a healthy looking unit it attracts people to buy and be selling healthy food even better, but there are enormous amount of challenges around the units and the key of that is well to forget the finance it costs money to build a unit but the storage and ability so when you operating at Bellville BTI where do you store it. And again, that is why I say my tension again as an official in the city and our politicians in the city – our politician turn around and say we want that, that is fine but then the practicality where do you store it.

P 4: SF Workshop_transcript.docx - 4:4 [in Cape Town a big issue is th..]  (7:7)   (Super)
Codes:	[Cart requirements] [Cart_challenges] 
No memos

in Cape Town a big issue is the weather. If we look at the extremes, a black south easter where you have to anchor things down, or a really bad rainy winter north wester where rain comes in sideways. There it is that onw would go for the three sided gazebos. It is very practical. Sturdy. So we should look at what the unit is being aimed for, can it cope with the weather?

P 4: SF Workshop_transcript.docx - 4:24 [Another comment I want to make..]  (41:41)   (Super)
Codes:	[Cart requirements] [Legislative requirements] 
No memos

Another comment I want to make is that when the design happens it obviously happens around public safety in terms of what is permitted. For example, a simple example is what is allowed in gas capacity that can be accommodated on a trolley like this? because  what you find is whatever gas capacity is permitted, the person would have 2-3 tanks also on board as part of their back-up, so those things, what is permitted from a public safety for petroleum gas and also how would that preparation happen if, when using gas. It is also taking those things into consideration in the design. The advice I mean is sometimes we design something without the users input. 

P 4: SF Workshop_transcript.docx - 4:27 [a lot of the input will also c..]  (55:55)   (Super)
Codes:	[Cart requirements] [Legislative requirements] 
No memos

 a lot of the input will also come from the environmental health colleagues, their basic requirements when it comes to the cart. Immediately I am thinking spit screens, all kinds of elements to make that cart sort of health compliant. Their requirements. It would be good to have their inputs as well. In the design that you may have. 

P 4: SF Workshop_transcript.docx - 4:28 [I think where it helps is arou..]  (67:67)   (Super)
Codes:	[Cart requirements] [Legislative requirements] 
No memos

 I think where it helps is around the preparation, on the kind of cart, or mechanism or material they use to prepare, that I think is important. If you look at the requirements imposed on them again the health and safety and the environmental health requirements, it is quite ominous on them, the traders. And obviously what they use is important and the design. 

______________________________________________________________________

Code: Cart_challenges {5-0}

P 3: Meeting_XXXXX.doc - 3:48 [Because to me, the units are f..]  (85:85)   (Super)
Codes:	[Cart requirements] [Cart_challenges] 
No memos

Because to me, the units are fantastic on one side because if you've got a healthy looking unit it attracts people to buy and be selling healthy food even better, but there are enormous amount of challenges around the units and the key of that is well to forget the finance it costs money to build a unit but the storage and ability so when you operating at Bellville BTI where do you store it. And again, that is why I say my tension again as an official in the city and our politicians in the city – our politician turn around and say we want that, that is fine but then the practicality where do you store it.

P 4: SF Workshop_transcript.docx - 4:4 [in Cape Town a big issue is th..]  (7:7)   (Super)
Codes:	[Cart requirements] [Cart_challenges] 
No memos

in Cape Town a big issue is the weather. If we look at the extremes, a black south easter where you have to anchor things down, or a really bad rainy winter north wester where rain comes in sideways. There it is that onw would go for the three sided gazebos. It is very practical. Sturdy. So we should look at what the unit is being aimed for, can it cope with the weather?

P 4: SF Workshop_transcript.docx - 4:5 [At the moment mobile vending i..]  (8:8)   (Super)
Codes:	[Cart_challenges] 
No memos

At the moment mobile vending in the CPT municipal area is not encouraged. So having something with wheels, why do you want wheels? If only to get it from storage to

P 4: SF Workshop_transcript.docx - 4:6 [Now the Gimme that unit, The Y..]  (8:8)   (Super)
Codes:	[Cart_challenges] 
No memos

 Now the Gimme that unit, The Yolanda … The basis behind that is complaints about traders taking up too much trading space. So trying to confine the vendor to the 1X2 trading space available. She then designed a unit to fit that space. Which was a pack up and go uinit.

P 4: SF Workshop_transcript.docx - 4:8 [The proposed vending structure..]  (10:10)   (Super)
Codes:	[Cart_challenges] 
No memos

The proposed vending structure a bit small. If I look at my area; the guy would have his hotdogs, his steak-rolls , his salomies and curries at ones stall. Cause as time goes by they tend to grow. 

______________________________________________________________________

Code: Cart_example {19-0}

P 2: Meeting EHManagers.doc - 2:2 [There was another cart we had ..]  (4:4)   (Super)
Codes:	[Cart_example] 
No memos

There was another cart we had a discussion now what was designed by the Cape Winelands municipality and they actually donated to their vendors I don't know if you know about that? 

P 2: Meeting EHManagers.doc - 2:3 [So maybe just look at the way ..]  (6:6)   (Super)
Codes:	[Cart_example] 
No memos

So maybe just look at the way their one was designed.

P 2: Meeting EHManagers.doc - 2:4 [Then about two years ago I als..]  (8:8)   (Super)
Codes:	[Cart_example] 
No memos

Then about two years ago I also attended they had also launched a similar product like this here in the airport industry for the fish guys so I don't know if you maybe interacted with those guys as well.

P 2: Meeting EHManagers.doc - 2:5 [Yes, they drop them off at cer..]  (13:13)   (Super)
Codes:	[Cart_example] [Traffic bylaw] 
No memos

Yes, they drop them off at certain points and so on. And those things also had wheels on and I mean there were guys from law enforcement and they didn't mention anything about this unit has a wheel is going to be a problem or anything because it was various departments also the informal traders as well as people from the business sector and then the vendors themselves and it was us and law enforcement and we gave some input on there – on their design. I'm just looking at the… For the normal vendor maybe the affordability could be a bit of a challenge.

P 2: Meeting EHManagers.doc - 2:9 [Because there's UCT in their d..]  (31:31)   (Super)
Codes:	[Cart_example] 
No memos

Because there's UCT in their department they were busy trying to design a stove a coal heating thing with less emissions but it uses less wood but I'll provide more details, but I will provide those details.

P 2: Meeting EHManagers.doc - 2:10 [It's sort of like a wood stove..]  (33:33)   (Super)
Codes:	[Cart_example] 
No memos

It's sort of like a wood stove.

P 2: Meeting EHManagers.doc - 2:11 [They design a bigger one but w..]  (35:35)   (Super)
Codes:	[Cart_example] 
No memos

They design a bigger one but we said no it's going to be heavy for carrying and moving around so they designed a smaller one.

P 2: Meeting EHManagers.doc - 2:15 [I think the cart of Cape Winel..]  (44:44)   (Super)
Codes:	[Cart requirements] [Cart_example] [R962] 
No memos

I think the cart of Cape Winelands will bring with it some answers to what you are looking for because they have a safe component in it there is I can't remember if it is a tap or 25 litre water container – clean water thing in whereby one can use the sink maybe to wash maybe your vegetables or whatever or the meat or anything and then at the bottom there is also an empty 25 litre container to drain the thing actually. So it's a typical – an ideal model that really satisfies us as EHPs because its got all the components of what we are looking for and then I think it also has a space for them to label their stalls because our regulations are R962 requires that if it is shop that you are selling then it must be clearly labeled with your name and everything.

P 3: Meeting_XXXXX.doc - 3:45 [And then maybe also I know tha..]  (81:81)   (Super)
Codes:	[Cart_example] 
No memos

And then maybe also I know that CPUT they have done through some of their final year students they develop a number of different models for mobile vending.

P 3: Meeting_XXXXX.doc - 3:49 [Yes, he has now moved he is no..]  (89:89)   (Super)
Codes:	[Cart_example] [Mobile cart business example] 
No memos

Yes, he has now moved he is now down in Ottery somewhere but the model there is that he will fund the building of that unit and then every morning he would stock the unit so I don't know how many he has at the moment, but I think about ten or 20, he will stock the units and then deliver it to the roadside place and then that is where the vendor will be selling and it's fresh fish and his whole business model it is healthy fish it is not old fish and it picks up your passing trade and he's trying first of all he's trying not to compete with your typical fish vendor from the Cape Flats he is trying to create an opportunity for new vendors to be on the Atlantic Seaboard than the posh areas. But, I don't know if that is what you are looking at because this is fresh fish that you have to take home to cook this is not selling food on the pavement which you can eat straight away.

P 4: SF Workshop_transcript.docx - 4:7 [She then designed a unit to fi..]  (8:8)   (Super)
Codes:	[Cart_example] 
No memos

She then designed a unit to fit that space. Which was a pack up and go uinit. The unit hire fee would then cover storage, security, insurance, wear and tear. So all you did as a vendor was to arrive at your bay. So you unlock and start trading. The unit was designed to be versatile. Where that unit failed was its costing structure. The trader just found it too expensive, even if realistically it was affordable, the traders did not find it affordable. 

P 4: SF Workshop_transcript.docx - 4:9 [The Coca-Cola vending stall/st..]  (11:11)   (Super)
Codes:	[Cart_example] 
No memos

The Coca-Cola vending stall/stand for example, for packaged snacks, cigarettes and beverages was sold at about R500. With a cooler on the side for beverages. It has a canopy. It covers them as well as their commodities. 

P 4: SF Workshop_transcript.docx - 4:12 [At the moment there is a proje..]  (22:22)   (Super)
Codes:	[Cart_example] 
No memos

 At the moment there is a project at CPUT and the Cape Town Partnership with the city's support, where they are designing a structure which will possibly be used for the entire CBD area and further on. There are 3 lecturers working on it, when Janice gets back we can ask her to get details. They have a few designs already. At the moment they are liaising with traders as to what they want etc. it is supposed to cater for various commodities. 

P 4: SF Workshop_transcript.docx - 4:14 [most units in “my” area in New..]  (25:25)   (Super)
Codes:	[Cart_example] 
No memos

most units in “my” area in Newlands is a skottelbraai. They don't have or come with fancy contraptions or innovative thinking, they come something that can fit in that 2X2 and something that can cook. And that's a skottelbraai. 

P 4: SF Workshop_transcript.docx - 4:18 [the fish cart model that Bruce..]  (29:30)   (Super)
Codes:	[Cart_example] 
No memos

the fish cart model that Bruce developed. He wanted to sell fresh fish on a daily basis. It had to be kept healthy, not exposed to the sun. If the unit looked sexy/attractive, customers would come. But the target was not the Cape Flats. It was more the Atlantic seaboard, the leafy suburbs, that type of market. 
He designed a unit with four wheels, a nice canopy. The only reason the unit had wheels was that he has a huge trailor where he could store 6-8 of them on. So he can wheel them on and off. The unit itself had iceboxes, it has a gas cooker, waste and water. And he did the business model. I can give you Bruce's details. That unit worked for fish. That unit was designed in conjunction with the city's health and law enforcement directorates. So it covered all the legislative aspects. It is bigger than your average trading bay. 

P 4: SF Workshop_transcript.docx - 4:20 [The focus there was clean and ..]  (37:37)   (Super)
Codes:	[Cart_example] 
No memos

The focus there was clean and smart. So it attracted the customer. 

P 4: SF Workshop_transcript.docx - 4:26 [Because they at CPUT, they sta..]  (51:51)   (Super)
Codes:	[Cart_example] 
No memos

Because they at CPUT, they started off with the 1st and 2nd year students just to do conceptual; designs and so on. It has now progressed to the stage where come 3rd year students are doing papers on it, but the 4th year students design a real model. But underpinning that is the discussion with city officials, the discussions with traders to see what works. So maybe we can tap into that instead of trying to re-invent the wheel. At CPUT they are looking at the costing, the feasibility etc. 

P 4: SF Workshop_transcript.docx - 4:39 [But for me the CPUT initiative..]  (109:109)   (Super)
Codes:	[Cart_example] [CPUT initiative] 
No memos

But for me the CPUT initiative is exciting because they are engaging with the informal sector, already engaging them, with them. 

P 1: Interview with XXXXX.docx - 1:6 [Yeah that may be a good idea. ..]  (34:34)   (Super)
Codes:	[Cart_example] 
No memos

Yeah that may be a good idea. Because I know there is a guy who is doing fish carts, and my colleagues had a look at it and I noticed that they had a lot of comments on it. 

______________________________________________________________________

Code: CCT's commitment to project {1-0}

P 4: SF Workshop_transcript.docx - 4:37 [And from the city's side we wo..]  (109:109)   (Super)
Codes:	[CCT's commitment to project] 
No memos

And from the city's side we would certainly love to continue being part of the project. And I know you meeting with our environmental health colleagues and I am sure you will find a way that we can continue. 

______________________________________________________________________

Code: CCT's infrastructure_challenge {8-0}

P 3: Meeting_XXXXX.doc - 3:80 [Now, if you are creating your ..]  (141:141)   (Super)
Codes:	[CCT's infrastructure_challenge] 
No memos

 Now, if you are creating your typical street vendor the opportunities on the street, it's difficult then for the city to provide water, electricity because ultimately you then creating shops on pavements and so we're exploring particularly in your established areas the creation of municipal infrastructure but then what that looks like we still early days it's a two year program and it's year one. Then maybe part of that is to provide electricity and then not necessary provide water, but access to water in proximity. And then the challenge of creating market together comes down to location so what we do, and it is in our program so when we refurbish any of our existing transport interchanges or build new ones, we immediately tap into that question, so where it is easy, is and I'm just thinking an example, we in Walasdene piece of ground three caravan traders and a few street vendors so we built a transport facility it's one the green star rating and then as part of that facility we built two kiosks to accommodate the two caravan vendors I said three but anyway, and then the opportunity for your street trading and there you have water, electricity there is a car wash facility so we do create a…

P 3: Meeting_XXXXX.doc - 3:81 [So the big challenge with that..]  (145:145)   (Super)
Codes:	[CCT's infrastructure_challenge] 
No memos

So the big challenge with that is in established areas and areas which we are establishing so where you got your built up areas already it is very difficult to provide that infrastructure and then also when you investing money in the informal economy in that area, the decision makers also look at the bad experiences and bad bits of practice come forward for example this is a silly example it's not related to food but in Adderley Street you've got the Trafalgar Place flower market now a lot of people look at the flower market and oh it's beautiful and it's wonderful and all that sort of thing and that is true, but yet those I forget the number of families there, they are habitual law breakers a number of cases so to talk to a city about upgrading that facility and investing money in it – it sounds goods but it's for tourists and that sort of thing but yet the beneficiaries of that have a record of law breaking so…

P 3: Meeting_XXXXX.doc - 3:82 [One of the other challenges th..]  (147:147)   (Super)
Codes:	[CCT's infrastructure_challenge] 
No memos

One of the other challenges that we have in terms of infrastructure is the nature of street vending to be mobile for the opportunity so this year there might be passing of foot traffic in this area, next year for some reason it changes. Now, we had a good example but it is a sad case where we built a fish market in Grassy Park and the business model and the numbers and everything worked out but that facility never took off so eventually we demolished that facility because it was just lying there and being abused by gangs and all that sort of thing. Because of that the city is very nervous about building something.

P 3: Meeting_XXXXX.doc - 3:83 [And also the city is not very ..]  (149:149)   (Super)
Codes:	[CCT's infrastructure_challenge] 
No memos

And also the city is not very good in being asset managers and facility managers so again simplistically what we do, we provide minimum infrastructure on hard surface water, electricity and we just on the water side we have been introduced to very recently that you can have prepaid water opportunities. 

P 3: Meeting_XXXXX.doc - 3:85 [So it's intensive but certainl..]  (155:155)   (Super)
Codes:	[CCT's infrastructure_challenge] [Storage_challenge] 
No memos

So it's intensive but certainly because of the historical negotiations took place with the town centre the issue of storage facilities is on that agenda there. City wise it causes a tension because first of all it's just city it's a municipality and business are providing storage facilities for traders and if your answer is yes are the traders prepared to pay for that and often you find that the traders are not prepared to pay for it.

P 3: Meeting_XXXXX.doc - 3:87 [But, what they are actually as..]  (155:155)   (Super)
Codes:	[CCT's infrastructure_challenge] 
No memos

But, what they are actually asking for it's a need they not disputing that but they want it provided and then it must be close to where they are and they don't want to pay for it. Now one of the tensions that I am having against my colleagues in the city is that in the tariff and charges apart from saying that if you trading here too the tariff goes up the tariff will also distinguish between are you having just a simply a pavement or are you having some sort of infrastructure and that cost goes up. Now, one would justify in terms of you got to pay for the services etcetera but I don't think the informal traders can afford to pay it.

P 3: Meeting_XXXXX.doc - 3:88 [And then that means that the b..]  (157:157)   (Super)
Codes:	[CCT's infrastructure_challenge] 
No memos

And then that means that the bulk of your traders can't trade I mean just an example one of the… And it is really a concern I'm not disputing it, but from the communities and unfortunately it's more your wealthy communities than your township Cape Flats community. I don't want traders to do it where there no toilet facilities. In some cases there is no public toilet so what do you do does the city then build public toilets because you've got vendors here. In some cases like just going to give you a silly example Cape Point the traders hire their own toilet facilities.

P 3: Meeting_XXXXX.doc - 3:89 [Yes, they've got a mobile vend..]  (159:159)   (Super)
Codes:	[CCT's infrastructure_challenge] 
No memos

Yes, they've got a mobile vending[?]. What we try and do simplistically is where they are vending they should have access to public toilets and water. If they don't then they must at least provide proof that they have access to some water or some private facility so there might be a petrol station where they go and use the toilets or something like that.

______________________________________________________________________

Code: Certificate of acceptability {11-0}

P 1: Interview with XXXXX.docx - 1:11 [And then of course the certifi..]  (44:44)   (Super)
Codes:	[Certificate of acceptability] 
No memos

And then of course the certificate of acceptability and things like that, you are obviously aware of that?

P 1: Interview with XXXXX.docx - 1:31 [And at the same time they shou..]  (87:87)   (Super)
Codes:	[Certificate of acceptability] 
No memos

And at the same time they should ideally get issued with their certificate of acceptability for which there is no cost implications. On the certificate they will obviously stipulate what they are allowed to sell, and not. The basically endorse that certificate and things like that. So if your stall allows you to do certain things and you now all of a sudden want to go and do other things, the stall may not be equipped to do that, or your cart may not be equipped to do that.

P 1: Interview with XXXXX.docx - 1:33 [You only need, no. you only ne..]  (89:89)   (Super)
Codes:	[Certificate of acceptability] 
No memos

 You only need, no. you only need a, that is quite right. COA for fruit and vegetables, do not need it. does not mean that you do not have to comply with requirements, because if there is a health nuisance we can act.

P 1: Interview with XXXXX.docx - 1:35 [But fruit and veg and fish at ..]  (89:89)   (Super)
Codes:	[Certificate of acceptability] [If non-compliant] 
No memos

But fruit and veg and fish at the moment is the one that we do not give an COA to. but those are the challenging ones because if they make a mess, we can get to them. So you know, I can, other legislation allow us to confiscate the goods, condemn it or contain it or things like that. 

P 1: Interview with XXXXX.docx - 1:41 [COA is if you have somebody an..]  (95:95)   (Super)
Codes:	[Certificate of acceptability] 
No memos

COA is if you have somebody and you have a stall and you put that somebody down as the person in charge and that person leaves, then you would re-apply for that certificate, but mostly for walkers, it remains the licensed walker as well.

P 2: Meeting EHManagers.doc - 2:28 [I will send it to you now. But..]  (55:55)   (Super)
Codes:	[Certificate of acceptability] [R962] 
No memos

I will send it to you now. But I mean like Johan was saying it covers the definitions, it covers Certificate of Acceptability which one of the things that you asked about certification that is required. 

P 2: Meeting EHManagers.doc - 2:35 [But, however, you've got to co..]  (61:61)   (Super)
Codes:	[Business license] [Certificate of acceptability] 
No memos

But, however, you've got to comply it doesn't matter where you stand whether it is private or whether it's on the open you still got to have both those pieces of paper, the business license dependent like for example the business license the COA will sell. The COAs is low cost the business license has got a ridiculous cost of R10.

P 3: Meeting_XXXXX.doc - 3:51 [There are two components the o..]  (98:98)   (Super)
Codes:	[Certificate of acceptability] [Permit] 
No memos

There are two components the one is what I call a location permit and the other one deals with your health certificate I'm going to use the broad word health certificate. 

P 3: Meeting_XXXXX.doc - 3:53 [But then the second side of it..]  (98:98)   (Super)
Codes:	[Business license] [Certificate of acceptability] 
No memos

But then the second side of it, it comes down to and this is more Christa's area has to deal with depending on the nature of the food that you are selling determines what permission you need. So you need a Certificate of Acceptability and you need business license those are the two key ones so if you are cooking food for example you need both those certificates.

P 3: Meeting_XXXXX.doc - 3:54 [It's a Certificate of Acceptab..]  (100:100)   (Super)
Codes:	[Business license] [Certificate of acceptability] [Confusion_licensing/permits] 
No memos

It's a Certificate of Acceptability and a business license. Now, I do need to say that those two bits of document cause confusion with the vendor because the vendor in his mind comes to the municipality and says I want to sell boerewors okay this is what you need and they go to the health department they gets those two bits pieces of paper and then they go down to Adderley Street and there is not trading and next thing law enforcement hits them hard, then I say but I got them… This just gives you permission from a health point of view to sell it doesn't give you a right to go onto private property and sell, it doesn't give you a right to go anywhere so you need the location permit. But, in the street vendors mind there is a lot of… [speaking simultaneously]

P 3: Meeting_XXXXX.doc - 3:56 [anywhere else in Mitchells Pla..]  (102:102)   (Super)
Codes:	[Certificate of acceptability] 
No memos

anywhere else in Mitchells Plain you don't need a location permit but then you need to get your Certificate of Acceptability and your business permit. 

______________________________________________________________________

Code: Challenges to selling healthy food {1-0}

P 3: Meeting_XXXXX.doc - 3:27 [What are the impediments to en..]  (50:50)   (Super)
Codes:	[Challenges to selling healthy food] 
No memos

What are the impediments to encourage healthy food? To me it is very easy to simply say you can't sell food that you have to fry and cook and you can do that.

______________________________________________________________________

Code: Child nutrition {1-0}

P 2: Meeting EHManagers.doc - 2:76 [And that is their meal or thei..]  (171:171)   (Super)
Codes:	[Child nutrition] [Vendors at schools] 
No memos

And that is their meal or their lunch for the afternoon or whatever, so maybe that is one of the focus areas because that is a sensitive area. It's either I know one or two schools have done it before, but that is in the school where you would have your tuck shop to look at their menu in the tuck shop but also then combine that with the vendor that is outside. I know at your more affluent schools they don't allow vendors but if you look at the Delft areas Mannenberg areas those areas die aunties sit almal so they all sit on the road.

______________________________________________________________________

Code: COA_requirements {5-0}

P 1: Interview with XXXXX.docx - 1:47 [We don't require a business pl..]  (101:101)   (Super)
Codes:	[COA_requirements] 
No memos

We don't require a business plan from them. But we do need to know what they are selling. And if that is a business plan or part of a business plan that would come out in their application and their inspection at home and thing like that, ok. so that would be good if they draw up a business plan, but that is just something very good, because that also guides them to specifically what it is that they require and it starts getting their thoughts together on how do they treat their business and how do they handle their business and I have to buy in so much and I have to start planning and things like that. And that is where reality hits home for them you know.

P 1: Interview with XXXXX.docx - 1:48 [Water, is obvious what they ha..]  (104:104)   (Super)
Codes:	[COA_requirements] 
No memos

 Water, is obvious what they have to have on the stand that is specific. And I will send those guidelines to you. We do not have electricity requirements. 

P 1: Interview with XXXXX.docx - 1:49 [Food safety. 5 keys of safety,..]  (107:107)   (Super)
Codes:	[5 keys to safer foods] [COA_requirements] 
No memos

 Food safety. 5 keys of safety, they are quite happy as far as that is concerned. Food covered refrigerated, temperature control. Temperature control, very important for them, absolutely important. 

P 1: Interview with XXXXX.docx - 1:50 [But that is why we accept the ..]  (109:109)   (Super)
Codes:	[COA_requirements] [Temperature control] 
No memos

But that is why we accept the cooler boxes, and so on. So if your product goes out of your home in the morning at 4 degrees Celsius, you have ice boxes and coolerbags and things like that, you can maintain it at a safe temperature, unless you are going to leave that bin open or that container open, or you going to open and close, open and close it, and things like that. So that is why I say that business model is very important. 

P 1: Interview with XXXXX.docx - 1:68 [Because when people apply for ..]  (146:146)   (Super)
Codes:	[COA_requirements] 
No memos

Because when people apply for their license we guide them to the COA as well. and a COA really and truly for somebody that only sells peanuts on the street and things like that, now really and truly, we have to do it but really it just becomes a little bit tedious and things like that. 

______________________________________________________________________

Code: Communication_municipal vs provincial {1-0}

P 3: Meeting_XXXXX.doc - 3:2 [I think to me one of the bigge..]  (11:11)   (Super)
Codes:	[Communication_municipal vs provincial] 
No memos

I think to me one of the biggest challenges would be that left hand doesn't talk to right hand and often conversations taking place like even within the municipal area I don't want to say within the city so you will have provincial government employing consultants to look at the informal economy and have all sorts of interviews and they don't necessary talk to the city. The city also does its own research and investigation and then aside from that you have the South Africa Local Government Association their Western Cape unit also doing similar work. What generally happens is that people somewhere along the conversation they say get hold of Paul and then suddenly then I am connecting the dots my question is about provincial government why you doing research on the city of Cape Town's informal trading policy so surely your starting point is talk to the city of Cape Town.

______________________________________________________________________

Code: Compliments CCT's vision {1-0}

P 3: Meeting_XXXXX.doc - 3:74 [It will certainly cut into the..]  (129:129)   (Super)
Codes:	[Compliments CCT's vision] 
No memos

It will certainly cut into the city's vision over -arching vision. In terms of economic development obviously hygiene and food security and healthy eating and healthy living that is part of our model. So to me the more interesting part is how does one facilitate and encourage the transition from current unhealthy food to… And then there is a component about I spoke earlier on about urban agriculture we trying to promote that.

______________________________________________________________________

Code: Compliments_salt reduction campaign {1-0}

P 2: Meeting EHManagers.doc - 2:44 [And maybe also fitting in with..]  (88:88)   (Super)
Codes:	[Compliments_salt reduction campaign] 
No memos

And maybe also fitting in with the Minister of Health the reduction of salt in the diets and all of those things and how this fits in because at the end of the day the consumer or the community or the people consuming these foods pay a heavy price maybe they think that they are scoring now by purchasing but 20 years later we having all of these… The hospitals are flooded and the rate we carrying on the health services that we are rendering in this country is not sustainable because of I mean we can see the strain all over the day hospitals, the hospitals, because of these diabetes, it's high blood, all of these things so it presents maybe not now, but ten 15/20 years later.

______________________________________________________________________

Code: Concession letters {1-0}

P 3: Meeting_XXXXX.doc - 3:90 [ou see there is a tension. let..]  (167:167)   (Super)
Codes:	[Allocated vs non-allocated zones] [Concession letters] [Vendor challenges] 
No memos

ou see there is a tension. let's just talk first of all about the 40% on municipal area that you don't need a location permit and just so that you can get a picture of it at the moment the Blouberg that whole area up the coast the whole of Khayelitsha there two pockets of Khayelitsha, but most of Khayelitsha town centre and then the area from Khayelitsha heading across towards Durbanville so that area is effectively a free trading area excluding the townships and the mentality of the community onto the politicians the ward councilor is that every single key informal trader is illegal so law enforcement will go there and the first thing is where is your permit. The fact that they talk to my staff and you don't need a permit the law says you don't need a permit but the mentality of law enforcement and the South African Police where's your permit and the trader gets very confused, I don't need a permit, but. So what we do and I'm only talking about the 40% of the area we issue what we call concession letters, its got no legal status it simply says you can trade these are the basic conditions and it is a piece of paper that the trader can get. Now, the councilors are very unhappy that we are issuing that letter and I have been challenged all over the place on it but I have succeeded with the challenge…

______________________________________________________________________

Code: Confusion_licensing/permits {1-0}

P 3: Meeting_XXXXX.doc - 3:54 [It's a Certificate of Acceptab..]  (100:100)   (Super)
Codes:	[Business license] [Certificate of acceptability] [Confusion_licensing/permits] 
No memos

It's a Certificate of Acceptability and a business license. Now, I do need to say that those two bits of document cause confusion with the vendor because the vendor in his mind comes to the municipality and says I want to sell boerewors okay this is what you need and they go to the health department they gets those two bits pieces of paper and then they go down to Adderley Street and there is not trading and next thing law enforcement hits them hard, then I say but I got them… This just gives you permission from a health point of view to sell it doesn't give you a right to go onto private property and sell, it doesn't give you a right to go anywhere so you need the location permit. But, in the street vendors mind there is a lot of… [speaking simultaneously]

______________________________________________________________________

Code: Consumer education {15-0}

P 2: Meeting EHManagers.doc - 2:63 [I think I mentioned it right a..]  (141:141)   (Super)
Codes:	[Consumer education] 
No memos

I think I mentioned it right at the beginning your consumer education you've got to really look at because one thing is the vendor but also the vendor will turn if there is no profit from overhauling or having this new approach, then the vendor would say but I am sorry, I'm paying R10 a day doing that now I'm making R5 a day doing this. You telling me long term I will make R12 a day but at the moment it's R5 a day I can't support my family on this. So that is an issue so your client is not buying into the concept.

P 2: Meeting EHManagers.doc - 2:64 [nd the education to the client..]  (142:142)   (Super)
Codes:	[Consumer education] 
No memos

nd the education to the client is also important to know if we can – I mean if I look at my job over the years, if you can educate the client more it will make my job so much easier because you will get somebody complaining coming to the office and saying listen, I bought food yesterday from that takeaway and they were handling their food with their hands and it doesn't look nice inside and you can now… Did you buy the stuff – yes, yes I bought it there I just want to complain about it. Why did you buy it? And that is the truth I would say if you could convince them then we don't have a job, but at the end of the day it will help a lot because if the premises or that little cart or where the guy is preparing the food is dirty and the customer said to him sorry, I'm not going to buy from you your place is dirty and everyone doesn't go to the nice guy he we don't have to fight with him his got to do it otherwise he is out of business. 

P 2: Meeting EHManagers.doc - 2:65 [I'm talking about from what le..]  (142:142)   (Super)
Codes:	[Consumer education] 
No memos

 I'm talking about from what level of population doesn't matter, they all do it, they do it in Constantia as well I suppose they buy from a place that doesn't look nice and they come and complain about it, but they did buy it and they didn't buy it and then show it, they went into the shop they see it is dirty, buy the stuff and then complain about it, and that's reality and that's a problem for us to get that together.

P 2: Meeting EHManagers.doc - 2:66 [The hygiene and health is not ..]  (145:145)   (Super)
Codes:	[Consumer education] [health and safety] 
No memos

The hygiene and health is not a big enough issue for the people out there. I mean if you can use the media and stuff like that to tell people just for instance sake labeling you've got to buy something and look at the label, the guy must look at what is on the label, if there is no date of expiry then just give it back to the guy, say no I don't want this product just for instance. If you can do that thing, but people with the media whose going to read something on the front of Die Burger if they said about the labeling that must have the date on, no, no… Look here, if somebody stirred the Parliament and a big fight then everybody is reading it, but put something about their health they don't do it.

P 2: Meeting EHManagers.doc - 2:67 [I would love to have that thin..]  (147:147)   (Super)
Codes:	[Consumer education] 
No memos

 I would love to have that thing where somebody can go on local radio stations because remember there are local radio stations who won't charge you for that, they've got slots here use this all this local radio Tygerberg and Bok Radio and all those who have got slots in the morning I listen to it sometimes when we driving up and down where they've got a lawyer in the studio, and all can phone in and ask the lawyer questions they don't pay for that, and they've got somebody about this, they've got it on all the radio stations. They've got a slot in the afternoon where somebody can phone about a vet, about their dogs and treating your animals, I mean there are slots like that but you never heard… Maybe we out of line here, but there is nothing about health I would love to get somebody on the radio and talk about these food vendors the five keys to safe health so that the listener can hear the stuff as well.

P 2: Meeting EHManagers.doc - 2:69 [But still, the minster is not ..]  (154:154)   (Super)
Codes:	[Consumer education] [Minister of health] 
No memos

But still, the minster is not going to talk about the five keys of health and the labeling and stuff, we need the ground level thing not on a big national radio there local radios playing in this area that area local where they understand the things.

P 2: Meeting EHManagers.doc - 2:70 [You don't want the people – yo..]  (160:160)   (Super)
Codes:	[Consumer education] 
No memos

You don't want the people – you don't want it on SABC one and two tonight everybody is not listening, you want it during the day when people are listening on the radio while they are preparing their food and they've got a little radio there this local thing.

P 2: Meeting EHManagers.doc - 2:71 [What I wanted to say earlier w..]  (161:161)   (Super)
Codes:	[Consumer education] 
No memos

What I wanted to say earlier when Vanesh was speaking on the education of the audience, was I remember last year we had a workshop where there was a nutritionist that came to do a presentation on healthy food, replacing that big plate which we showed you earlier with more of other options and I remember the resilience[?] that was on the floor, so yes because they part of the because [unclear 1:06:05] is working with all the sick people and sometimes people believe in them more than the doctors and the clinic. So then just for them to also give out education on healthier options and what to eat and so on. But, I think it will be follow up sessions will be needed and things like that until then they themselves they buy into it because then you know they are showing in a party instead of having snacks, sweets and all those things to replace them with carrots, cucumbers [speaking simultaneously]. But, they were just looking at it and this other one no, no, that is not possible but I mean they are…

P 2: Meeting EHManagers.doc - 2:72 [And they are also the first co..]  (163:163)   (Super)
Codes:	[Consumer education] 
No memos

And they are also the first contact in the communities where people do consultations and put out this message of healthy eating and so on. So I was just saying I wanted to add on to what Vanesh was saying earlier that you know and maybe what you will be requesting from them because depending on the audience and depending on the who will be attending the workshops and whatever that…

P 2: Meeting EHManagers.doc - 2:73 [I just wanted to say something..]  (164:164)   (Super)
Codes:	[Consumer education] [Culture] 
No memos

I just wanted to say something people we think inside the box, we are supposed to be outside the box I'm thinking about like you saying that meeting was all about people caring for people who are sick or whatever if you ask about the sicknesses of those people, they come from the food that they eat, but people are concentrating on the sick person now, and not trying to change the environment that made that particular person to be sick. It's good I know they always okay when you talk health you not wanted anyway but by asking always out there then we one way or the other we going to be accepted by the community because things have changed. I remember the way there were times where you wouldn't even talk about things that relates to nutrition because people are going to tell you that no, it's my culture and all those kind of things but nowadays people are changing because when you try to ask them how is it your culture and they will tell you, my grandmother used to do it like this and whatever I don't want to change the recipe and all those kind of things, but when you probe the person how did your mother prepare it or your grandmother, and then it goes back to less salt, less fat and just like that because it started without having all these and because now we can buy more ingredients and then we improve it and it's all about that like I'm trying to say we just need to be our own advocates actually, make sure that we talk about that they should get sick of us, but we should talk about it.

P 2: Meeting EHManagers.doc - 2:87 [I think like Johan said you ju..]  (223:223)   (Super)
Codes:	[Consumer education] [Culture] 
No memos

I think like Johan said you just need to target the consumers because some people especially Xhosa men really we were cultured to… In a way that men of the house must really the plate of the men of the house must not be same as mine plate or my child's plate so that's how our men are used to. 

P 2: Meeting EHManagers.doc - 2:88 [So now we just slowly need to ..]  (223:223)   (Super)
Codes:	[Consumer education] [Culture] 
No memos

So now we just slowly need to do that changing of the mindset that although you can still get just reduce your quantities because to us really when a person dishes at least three full spoons of rice and the plate is already like this, then the other vegetables must still go… That is how we are you go to each and every woman in the township three full spoons.

P 2: Meeting EHManagers.doc - 2:89 [We eat from the same pot from ..]  (224:224)   (Super)
Codes:	[Consumer education] 
No memos

We eat from the same pot from within the same houses the same food maybe the same amount of food like my husband and I but then when we are serving we find women are better than… I'm trying to think about the mindset because you tell those people no don't eat too much and they tell you no I am not fat and I've got to use all that kind of energy which is fine at some stage, but there is a stage where they not going to be working so hard or bending over kind of energy that they because our men when they at a certain age they fine, but once that time comes and then you see pot bellies and…

P 2: Meeting EHManagers.doc - 2:92 [It's the same as the ads on TV..]  (240:240)   (Super)
Codes:	[Consumer education] 
No memos

It's the same as the ads on TV when they show this little thin woman there eating whatever it is she's eating and they selling and there are still people believing it, that's the biggest problem for me not what they do, but there are still people believing them.

P 3: Meeting_XXXXX.doc - 3:31 [To me the approach would be tw..]  (57:57)   (Super)
Codes:	[Consumer education] [Vendor education] 
No memos

To me the approach would be two fold one is to educate the trader absolutely but one needs to also educate the community and it has taken me a long time to suddenly realize that what I am eating is unhealthy and most of the time I change my…

______________________________________________________________________

Code: CPUT initiative {1-0}

P 4: SF Workshop_transcript.docx - 4:39 [But for me the CPUT initiative..]  (109:109)   (Super)
Codes:	[Cart_example] [CPUT initiative] 
No memos

But for me the CPUT initiative is exciting because they are engaging with the informal sector, already engaging them, with them. 

______________________________________________________________________

Code: Culture {4-0}

P 2: Meeting EHManagers.doc - 2:73 [I just wanted to say something..]  (164:164)   (Super)
Codes:	[Consumer education] [Culture] 
No memos

I just wanted to say something people we think inside the box, we are supposed to be outside the box I'm thinking about like you saying that meeting was all about people caring for people who are sick or whatever if you ask about the sicknesses of those people, they come from the food that they eat, but people are concentrating on the sick person now, and not trying to change the environment that made that particular person to be sick. It's good I know they always okay when you talk health you not wanted anyway but by asking always out there then we one way or the other we going to be accepted by the community because things have changed. I remember the way there were times where you wouldn't even talk about things that relates to nutrition because people are going to tell you that no, it's my culture and all those kind of things but nowadays people are changing because when you try to ask them how is it your culture and they will tell you, my grandmother used to do it like this and whatever I don't want to change the recipe and all those kind of things, but when you probe the person how did your mother prepare it or your grandmother, and then it goes back to less salt, less fat and just like that because it started without having all these and because now we can buy more ingredients and then we improve it and it's all about that like I'm trying to say we just need to be our own advocates actually, make sure that we talk about that they should get sick of us, but we should talk about it.

P 2: Meeting EHManagers.doc - 2:87 [I think like Johan said you ju..]  (223:223)   (Super)
Codes:	[Consumer education] [Culture] 
No memos

I think like Johan said you just need to target the consumers because some people especially Xhosa men really we were cultured to… In a way that men of the house must really the plate of the men of the house must not be same as mine plate or my child's plate so that's how our men are used to. 

P 2: Meeting EHManagers.doc - 2:88 [So now we just slowly need to ..]  (223:223)   (Super)
Codes:	[Consumer education] [Culture] 
No memos

So now we just slowly need to do that changing of the mindset that although you can still get just reduce your quantities because to us really when a person dishes at least three full spoons of rice and the plate is already like this, then the other vegetables must still go… That is how we are you go to each and every woman in the township three full spoons.

P 2: Meeting EHManagers.doc - 2:90 [You must also not forget that ..]  (226:226)   (Super)
Codes:	[Culture] 
No memos

You must also not forget that you talking about I do see the culture aspect of your [unclear 1:25:51] you must also not forget that if I take my wife home to my parents if she is thin then that means I don't care of her so [speaking simultaneously]. If she is well fed she is nice and chubby then she is happier and… So we cannot ignore those.

______________________________________________________________________

Code: EHP_duties {4-0}

P 1: Interview with XXXXX.docx - 1:66 [And you know it is restaurants..]  (146:146)   (Super)
Codes:	[EHP_duties] 
No memos

And you know it is restaurants, it is informal traders, it is complaints, and it is all sorts of things. It is different trades, so there is a host of activities that the colleagues have to attend to and I can well understand if they do not always get to a new hawker and is able to guide them. The responsibility is on the person, to come to us. not for us to chase them. 

P 2: Meeting EHManagers.doc - 2:37 [No, no there is a – we re-look..]  (67:67)   (Super)
Codes:	[EHP_duties] 
No memos

No, no there is a – we re-looking at but our performance management system is based on visiting each formal area and informal area at least once every six months.

P 2: Meeting EHManagers.doc - 2:38 [Obviously, if you have a probl..]  (69:69)   (Super)
Codes:	[EHP_duties] 
No memos

Obviously, if you have a problem at a particular area or particular shop you would go back more but the bare minimum like if you go to for example a Woolworths they've got a very high corporate image so the chances are that will let something the quality or something influence the quality of the product slide is very minimal so you would probably go there once every six months just to do an inspection and see if everything is fine. But, the moment you go to a shop where you will see I've got problems here you would go more often until the problem is resolved, but the bare minimum is six months.

P 2: Meeting EHManagers.doc - 2:39 [You will know your area if you..]  (70:70)   (Super)
Codes:	[EHP_duties] 
No memos

You will know your area if you do a specific area you will know your area you will know which business is registered, if they are not registered you must be part of the registration and then the inspection onto that will be according to just what he said now. There are some places you've got to visit one month or whatever it is I'm just it's a figure but you will know your area and you will know, so you will know who are registered or not.

______________________________________________________________________

Code: Facilities provided not used {2-0}

P 3: Meeting_XXXXX.doc - 3:23 [I'm going to call it a market ..]  (46:46)   (Super)
Codes:	[Facilities provided not used] 
No memos

I'm going to call it a market but we built the facility with the intention that it be a distribution point for the fruit and vegetable growers in the Philippi farm lands it didn't work. And I still don't know why it didn't work so we sitting with a problem we got a huge facility which is costing money but I think we've got 20 ripening rooms which is ideal for bananas and avos and mangoes and that I think we are using a third of it if we are lucky and the rest is being used for storage. And to me that's why I am talking about the challenges of the business model but the way I think we can address it is creating the opportunities and educating trading and what is available. 

P 3: Meeting_XXXXX.doc - 3:24 [I mean just using that as an e..]  (48:48)   (Super)
Codes:	[Facilities provided not used] 
No memos

I mean just using that as an example we got about eight fruit and vegetable they call themselves traders but they not, they fruit and vegetable distributors but they are using the town centre as a distribution point and they overnight they call all sort of challenges so I said to them literally down the road to get into your car and drive straight down the road it's eight - ten minutes there's the Philippe fresh produce market and they just said sorry it's just not cost effective for us. And I'm struggling to do that, that's why I say at the end of the day for me what would make sense how far do we go to become prescriptive as government on people in terms of their business model.

______________________________________________________________________

Code: Finance assistance {4-0}

P 3: Meeting_XXXXX.doc - 3:75 [We have a unit within economic..]  (131:131)   (Super)
Codes:	[Finance assistance] 
No memos

We have a unit within economic development called business support and we refer people to do it through that and then throughout the municipal area there are organizations which the city funds we can provide that and that helps you develop your business plan, your business case, looks at various financial options so those models are available.

P 3: Meeting_XXXXX.doc - 3:76 [So we have a number of organiz..]  (135:135)   (Super)
Codes:	[Finance assistance] 
No memos

So we have a number of organizations they will then assess the vendor so it's almost a two way what are you selling, what are you interested in, what are your skills and then say okay, this is what we think and this is the financial options available, this is your business plan.

P 3: Meeting_XXXXX.doc - 3:77 [Yes, not directly through the ..]  (137:137)   (Super)
Codes:	[Finance assistance] 
No memos

Yes, not directly through the city, but their financial options but various financial institutions have finances available it's just to know how to tap into that.

P 3: Meeting_XXXXX.doc - 3:78 [I mean you know, last year Abs..]  (139:139)   (Super)
Codes:	[Finance assistance] 
No memos

I mean you know, last year Absa was really promoting, making finance available for young entrepreneurs but you need to know how to tap into it. If you go to Absa enquiries counter…

______________________________________________________________________

Code: Food labelling {1-0}

P 1: Interview with XXXXX.docx - 1:10 [Otherwise the food labelling b..]  (44:44)   (Super)
Codes:	[Food labelling] 
No memos

Otherwise the food labelling becomes a problem. Er and so on, okay. 

______________________________________________________________________

Code: Food sampling {3-0}

P 2: Meeting EHManagers.doc - 2:57 [Safety product, what we do is ..]  (116:116)   (Super)
Codes:	[Food sampling] 
No memos

Safety product, what we do is we also do random sampling as well like food sampling. It is easier to do it when you have fixed premises for example then you can go back and give the results like we have at Bellville that station we on a quarterly basis take samples there because we struggling with the upgrade but in the meanwhile we trying to at least have sampling should… Because you need to guard against food poisoning as well, so that is one of the things that can be used as well.

P 2: Meeting EHManagers.doc - 2:58 [Guys I must tell you that some..]  (123:123)   (Super)
Codes:	[Food sampling] 
No memos

Guys I must tell you that some of these results are better than the formal trading.

P 2: Meeting EHManagers.doc - 2:59 [But, you see that is also prod..]  (125:125)   (Super)
Codes:	[Food sampling] 
No memos

But, you see that is also product dependent – it depends what you sell because you get your more sensitive products as well and you get other products where your bacteria and whatever is cooked or when it is properly prepared that's not an issue some of the times but you do get your sensitive and you're not so sensitive approach.

______________________________________________________________________

Code: Food vending (sml in number) {2-0}

P 4: SF Workshop_transcript.docx - 4:32 [I think the difficulty for us ..]  (84:84)   (Super)
Codes:	[Food vending (sml in number)] 
No memos

I think the difficulty for us when we developing these trading plans are we not really specific about who is going to sell what. we just create the plan and put whoever is to go into that space. To find those people selling food is few and in-between. 

P 4: SF Workshop_transcript.docx - 4:33 [In Wynberg and Claremont I hav..]  (86:86)   (Super)
Codes:	[Food vending (sml in number)] 
No memos

In Wynberg and Claremont I have two people who sell boerewors or prepared foods, I that context. And everybody else sells everything else, but we have that information for you. 

______________________________________________________________________

Code: Formulation of informal trading policy {2-0}

P 3: Meeting_XXXXX.doc - 3:5 [And then I won't go into detai..]  (25:25)   (Super)
Codes:	[Formulation of informal trading policy] 
No memos

And then I won't go into detail, but there were a combination of factors within the city of Cape Town where we were of the view that we needed to form an informal trading policy and in the late 90s and early 2000s we did extensive work around informal economy in Cape Town and that resulted in a policy framework which was approved by our council. An enormous amount of work was done within informal traders, with anybody who is interested in the informal economy and it's interesting the moment that document was approved by our council, the other major cities got to hear about it and they sent delegations down to see us, we made that document available and that started a process where my counterparts in the other major cities and I met together on a regular basis to talk about the informal economy and more specifically street trading to try and learn lessons from each other and that resulted in us then together with the South African Local Government Association do you know SALGA at all?

P 3: Meeting_XXXXX.doc - 3:6 [What we did there effectively ..]  (29:29)   (Super)
Codes:	[Formulation of informal trading policy] 
No memos

What we did there effectively is from all the different municipalities major and small we took lessons learnt and pulled in all those lessons[?] to formulate a generic policy and bylaw and that document now has been sent out to every single municipality saying this is the basket, you can choose what aspect is relevant for you or what is not relevant for you. So that document is available sorry I am just going to make notes otherwise I am going to forget.

______________________________________________________________________

Code: health and safety {3-0}

P 2: Meeting EHManagers.doc - 2:23 [I think the main thing with en..]  (48:48)   (Super)
Codes:	[health and safety] 
No memos

I think the main thing with environmental health I think the hygiene and the safety guidelines is more our line. I can't speak about viability of the business or the recipe book or stuff like that. And I'm always afraid to speak for another department.

P 2: Meeting EHManagers.doc - 2:55 [You said in your survey raw an..]  (113:113)   (Super)
Codes:	[health and safety] 
No memos

You said in your survey raw and cooked products the percentage was very high but it wasn't separated from each other so those are the things that you got to link to that and then that will improve the business of the person as well. 

P 2: Meeting EHManagers.doc - 2:66 [The hygiene and health is not ..]  (145:145)   (Super)
Codes:	[Consumer education] [health and safety] 
No memos

The hygiene and health is not a big enough issue for the people out there. I mean if you can use the media and stuff like that to tell people just for instance sake labeling you've got to buy something and look at the label, the guy must look at what is on the label, if there is no date of expiry then just give it back to the guy, say no I don't want this product just for instance. If you can do that thing, but people with the media whose going to read something on the front of Die Burger if they said about the labeling that must have the date on, no, no… Look here, if somebody stirred the Parliament and a big fight then everybody is reading it, but put something about their health they don't do it.

______________________________________________________________________

Code: Healthy food = expensive {4-0}

P 3: Meeting_XXXXX.doc - 3:32 [And then to me the frustration..]  (61:61)   (Super)
Codes:	[Healthy food = expensive] 
No memos

And then to me the frustration of the healthy food would be Woolworths or Checkers it is also more expensive than the…

P 3: Meeting_XXXXX.doc - 3:33 [No, no, but when you look at i..]  (63:63)   (Super)
Codes:	[Healthy food = expensive] 
No memos

No, no, but when you look at it when I've got a choice of buying sushi which costs R50 or go and buy a sandwich which costs R12, I will buy…

P 3: Meeting_XXXXX.doc - 3:34 [You not with me, I'm saying wh..]  (71:71)   (Super)
Codes:	[Healthy food = expensive] 
No memos

You not with me, I'm saying what is on the shelf when you see the price tag, the salad at Checkers and Woolworths is around about R40, so my choice is, do I go and buy a R40 worth of food or do I go and buy a R12 sandwich?

P 3: Meeting_XXXXX.doc - 3:35 [And I also think that Woolwort..]  (73:73)   (Super)
Codes:	[Healthy food = expensive] 
No memos

And I also think that Woolworths and Checkers they need to relook at their pricing structure because as you say, I don't believe a salad should be R40 it can easily be R15.

______________________________________________________________________

Code: Healthy food_focus {1-0}

P 3: Meeting_XXXXX.doc - 3:43 [the commodities they trade and..]  (75:75)   (Super)
Codes:	[Healthy food_focus] 
No memos

the commodities they trade and there is an element now coming through more of the nutritional healthy food it's still very low key not in your face

______________________________________________________________________

Code: How to market SFVM {1-0}

P 2: Meeting EHManagers.doc - 2:50 [There are two things that I wa..]  (107:107)   (Super)
Codes:	[How to market SFVM] 
No memos

There are two things that I want to highlight is how you going to sell this to whoever is going to want to pursue this direction. So how you going to sell you need to look at something how you going to present it to this person so we will have to look at a communication strategy with the people that you work with either be it in a workshop format or whatever the case may be which will include all the things that Zandile has spoken about. In other words if you do, you have to package this thing for the person and introduce this whole concept with a sort of a package to say this is your requirement this is what you need, this is whatever, whatever. That will be important and bearing in mind the careful nature of how you approach things with people. Then, you going to look at, not all people would go, because you get different people who sell different products as well so you got to focus on those that want to completely overall their business and taking on this business model, but there are some that would take on this model partially like you said earlier with the vetkoek for example you have a lot of mince but now introduce veggies into your mince for instance and reduce your content of your mince. So you've got to look at different strategies how to introduce your topic when you have a workshop.

______________________________________________________________________

Code: Hygiene practices {3-0}

P 2: Meeting EHManagers.doc - 2:60 [liked the point that you highl..]  (126:126)   (Super)
Codes:	[Hygiene practices] 
No memos

 liked the point that you highlighted about our model where someone is going to sit with the cart and people can stand around you. I think that on its own is enforcing will make people be cautious of their business environment. When you saying there is more contamination from your own studies you assume that there is more contamination with the actual form of business?

P 2: Meeting EHManagers.doc - 2:61 [Because you get the caravans f..]  (129:129)   (Super)
Codes:	[Hygiene practices] 
No memos

Because you get the caravans for example or those sort of stands which are quite closed it's very dingy inside of them whereas you have something that is completely open.

P 2: Meeting EHManagers.doc - 2:62 [It's more fresh that's true.]  (131:131)   (Super)
Codes:	[Hygiene practices] 
No memos

It's more fresh that's true.

______________________________________________________________________

Code: If non-compliant {3-0}

P 1: Interview with XXXXX.docx - 1:34 [does not mean that you do not ..]  (89:89)   (Super)
Codes:	[If non-compliant] 
No memos

does not mean that you do not have to comply with requirements, because if there is a health nuisance we can act. If the product is unsafe we can act. If you selling biscuits, chocolates things like that, sweets and things like that, technically you need it. 

P 1: Interview with XXXXX.docx - 1:35 [But fruit and veg and fish at ..]  (89:89)   (Super)
Codes:	[Certificate of acceptability] [If non-compliant] 
No memos

But fruit and veg and fish at the moment is the one that we do not give an COA to. but those are the challenging ones because if they make a mess, we can get to them. So you know, I can, other legislation allow us to confiscate the goods, condemn it or contain it or things like that. 

P 3: Meeting_XXXXX.doc - 3:62 [a lot of our councilors are sa..]  (110:110)   (Super)
Codes:	[If non-compliant] 
No memos

 a lot of our councilors are saying informal traders are not very compliant in terms of rules and regulations they over trade they litter, they you know so therefore the pressure to increase your law enforcement capacity and law enforcement costs money. 

______________________________________________________________________

Code: Incentivisation {2-0}

P 4: SF Workshop_transcript.docx - 4:22 [If the city is serious about g..]  (41:41)   (Super)
Codes:	[Incentivisation] 
No memos

If the city is serious about getting food out there that is acceptable standard, we must look at the design of a trolley of this nature, it may be this design would be applicable to a area where you have a covered area where you don't have those elements of wind etc. so you know it would also work around location. Maybe also as part of a long term design it is the city needs to be convinced. The powers that be, maybe give incentives to those who take up a trolley of this nature. Incentivise them. I think your presentation spoke a lot about, what's the word, subsidisation. So incentivise that, if you have this trolley we can look at it in terms of the tariff perhaps that they are charged to trade, that could also be something that we can consider. 

P 4: SF Workshop_transcript.docx - 4:38 [And one of the things I have f..]  (110:110)   (Super)
Codes:	[Incentivisation] 
No memos

And one of the things I have flagged here, you know you are talking about sustainability and creating that incentive and it is wrought with all types of challenges. But if someone were to come to the city and say I am an accredited healthy food vendor; do we give them a reduced tariff for a trading bay? Do we give them other kinds of services; the rest of the conventional trader doesn't have access to? It is wrought with difficulties but it is an opportunity. If you have a cart with solar panels you get 10 stars instead of one. 

______________________________________________________________________

Code: Informal trader law enforcement agency {1-0}

P 4: SF Workshop_transcript.docx - 4:30 [But a lot of stuff you are aim..]  (82:82)   (Super)
Codes:	[Informal trader law enforcement agency] [Legislative requirements] 
No memos

But a lot of stuff you are aiming at I think it should be driven by our policy. I mean the health should be driven through our policy. I mean the health issue should be through our health department. It should be a mandatory thing. people out there should wear gloves, the policy should stipulate you know the gloves or type of material that should be used or whatever. And those things should be enforced by law enforcement that is why they are there. That is why we have the informal trader law enforcement agency. 

______________________________________________________________________

Code: Informal trading plan {1-0}

P 3: Meeting_XXXXX.doc - 3:13 [Now, in Cape Town one of the a..]  (37:37)   (Super)
Codes:	[Informal trading plan] 
No memos

Now, in Cape Town one of the areas that we are focusing on is developing what we call an informal trading plan for an area so in terms of the 91 legislation as well, except the language that they use is different, so we will look at an area and say okay for various reasons we want to create an informal trading plan in this area and then within in that geographic area we determine where best trading should take place or shouldn't take place and then also what sort of trading should take place. 

______________________________________________________________________

Code: Informal trading summit {2-0}

P 3: Meeting_XXXXX.doc - 3:11 [And then just talking about th..]  (37:37)   (Super)
Codes:	[Informal trading summit] 
No memos

And then just talking about the legal framework just to sort of close that chapter, in I think it was March or May last year, our mayor had an informal trading summit she called all the different informal trading organizations together and shared the city's vision on informal economy and got input from them. And interesting when I just look at the broad profile of the informal sector that attended that summit in terms of food I think one of the key players it wasn't the majority player was the fruit and vegetable industries. And then maybe one or two people who sold fresh fish but the majority had to deal with your clothing and so… 

P 3: Meeting_XXXXX.doc - 3:12 [And then from that summit the ..]  (37:37)   (Super)
Codes:	[Informal trading summit] [Revision of policy + bylaws] 
No memos

And then from that summit the city has revised its policy and bylaws, so I can give you copies of that. But, again, it wasn't focusing on food and health specifically more a generic document to say this is how we want to grow and enhance the sector. 

______________________________________________________________________

Code: Inspections {2-0}

P 2: Meeting EHManagers.doc - 2:40 [Vendors are very difficult bec..]  (72:72)   (Super)
Codes:	[Inspections] 
No memos

Vendors are very difficult because you must remember a vendor can come to an office in Brakenfell registered as a vendor whatever, but he's going to hawk all over he can today be in Gordon's Bay and tomorrow in Simonstown that's the difficult part of it to hands on with that. A hawker has still got a base premises which is normally his house or somewhere where he is taking that little trolley tonight which we do inspections as well. But now, for instance if a guy is in a certain area and he is staying in Bellville and he has licensed now in Bellville but his hawking in Gordon's Bay and in Table View and in Simonstown and all over, so that cart of his is difficult to be hands on, on the cart because you don't know where she is tomorrow.

P 2: Meeting EHManagers.doc - 2:41 [And in some instances for inst..]  (73:73)   (Super)
Codes:	[Inspections] 
No memos

And in some instances for instance in Khayelitsha you have like stationed hawkers we do visit them especially the ones that are in containers at the taxi rank and depending on the problem like my colleague was saying earlier depending on the problem that is there and we do conduct a health education from time to time now with there's  a theme you would conduct health education with them every other time this is the thing around that particular season then you would conduct health education with them especially the ones who are…

______________________________________________________________________

Code: Kiosks {1-0}

P 4: SF Workshop_transcript.docx - 4:15 [Then you get individuals in CP..]  (26:26)   (Super)
Codes:	[Kiosks] 
No memos

Then you get individuals in CPT centre we moved them into kiosks with all their trims and fittings. 

______________________________________________________________________

Code: Law enforcement_complaints {1-0}

P 3: Meeting_XXXXX.doc - 3:93 [So law enforcement does react ..]  (173:173)   (Super)
Codes:	[Law enforcement_complaints] 
No memos

So law enforcement does react on complaints and if there are particular problematic areas they do deal with those.

______________________________________________________________________

Code: Legislation {1-0}

P 1: Interview with XXXXX.docx - 1:42 [The license and the certificat..]  (95:95)   (Super)
Codes:	[Legislation] 
No memos

The license and the certificate are both governed by legislation and things like that. 

______________________________________________________________________

Code: Legislative requirements {4-0}

P 4: SF Workshop_transcript.docx - 4:24 [Another comment I want to make..]  (41:41)   (Super)
Codes:	[Cart requirements] [Legislative requirements] 
No memos

Another comment I want to make is that when the design happens it obviously happens around public safety in terms of what is permitted. For example, a simple example is what is allowed in gas capacity that can be accommodated on a trolley like this? because  what you find is whatever gas capacity is permitted, the person would have 2-3 tanks also on board as part of their back-up, so those things, what is permitted from a public safety for petroleum gas and also how would that preparation happen if, when using gas. It is also taking those things into consideration in the design. The advice I mean is sometimes we design something without the users input. 

P 4: SF Workshop_transcript.docx - 4:27 [a lot of the input will also c..]  (55:55)   (Super)
Codes:	[Cart requirements] [Legislative requirements] 
No memos

 a lot of the input will also come from the environmental health colleagues, their basic requirements when it comes to the cart. Immediately I am thinking spit screens, all kinds of elements to make that cart sort of health compliant. Their requirements. It would be good to have their inputs as well. In the design that you may have. 

P 4: SF Workshop_transcript.docx - 4:28 [I think where it helps is arou..]  (67:67)   (Super)
Codes:	[Cart requirements] [Legislative requirements] 
No memos

 I think where it helps is around the preparation, on the kind of cart, or mechanism or material they use to prepare, that I think is important. If you look at the requirements imposed on them again the health and safety and the environmental health requirements, it is quite ominous on them, the traders. And obviously what they use is important and the design. 

P 4: SF Workshop_transcript.docx - 4:30 [But a lot of stuff you are aim..]  (82:82)   (Super)
Codes:	[Informal trader law enforcement agency] [Legislative requirements] 
No memos

But a lot of stuff you are aiming at I think it should be driven by our policy. I mean the health should be driven through our policy. I mean the health issue should be through our health department. It should be a mandatory thing. people out there should wear gloves, the policy should stipulate you know the gloves or type of material that should be used or whatever. And those things should be enforced by law enforcement that is why they are there. That is why we have the informal trader law enforcement agency. 

______________________________________________________________________

Code: M'Plain Towncentre {1-0}

P 3: Meeting_XXXXX.doc - 3:84 [I think one has to distinguish..]  (151:151)   (Super)
Codes:	[M'Plain Towncentre] [Storage_challenge] 
No memos

I think one has to distinguish between the Mitchells Plain town centre as a city wide model but you don't know in Mitchells Plain as the part of the whole discussion I don't know if you know of the model there, but really of an old bus terminus and effectively what we did we created a market underneath that terminus so it is an undercover market under roof market and then along the edges you've got kiosks and you have different size kiosks, you have large kiosks and you have small kiosks. But, the bulk of the not the bulk but the sizeable number of traders in the town centre are fruit and vegetable traders but there are also some who are not fruit and vegetable traders and the question is you know, when your means of transport is a mini bus taxi, what do you do with your goods at the end of the day.

______________________________________________________________________

Code: Methods of info dissemination {2-0}

P 3: Meeting_XXXXX.doc - 3:26 [One of the things that has hap..]  (50:50)   (Super)
Codes:	[Methods of info dissemination] 
No memos

One of the things that has happened because of the mayors having had summit last year and we had a follow up beginning of this year, is that we have access a large number of informal trading organizations and maybe what we should be doing is somehow work out how it works is to distribute this type of information to the informal sector and then perhaps even engage with the informal sectors I'm talking more about the food side of it not their… And to say this is healthy food and this is not healthy now how can we work together. 

P 3: Meeting_XXXXX.doc - 3:29 [And that maybe the education a..]  (52:52)   (Super)
Codes:	[Methods of info dissemination] 
No memos

And that maybe the education and opportunities.

______________________________________________________________________

Code: Might not require license/COA {3-0}

P 1: Interview with XXXXX.docx - 1:64 [Also the question I think abou..]  (145:145)   (Super)
Codes:	[Might not require license/COA] 
No memos

Also the question I think about what people are selling, so you may find hawkers that may not require a license but when you ask them the question they might think they need a license, because they may want something in paper. That is also a sense of , of, people that are trading informally, and that I think are under scrutiny or maybe because of where they are trading, they may want a piece of paper that says I am legitimately here.

P 1: Interview with XXXXX.docx - 1:67 [rhm and I suppose to a certain..]  (146:146)   (Super)
Codes:	[Might not require license/COA] 
No memos

rhm and I suppose to a certain extent it is difficult for people coming from rural areas coming here and trying to make a living and things like that. And let I just say the question about when do they need the licenses, that is the key. They sometimes think they need a license but they don't need that license, and that is sometimes where the and depending on who you interviewed that say they have not got something, the 60% that does not have permits and things like that how many of them required a license and how many did not. That would be the finer detail that you would then need to go into, to see is that 60% a 100 people and of that 100 people how many sold just sweets and how many just sold, and I am not talking about COA, I am just talking license. 

P 2: Meeting EHManagers.doc - 2:43 [You must remember with the lic..]  (77:77)   (Super)
Codes:	[Might not require license/COA] 
No memos

You must remember with the licensing and the COA not all of the hawkers need a business license and a COA it's only those who prepare food so you've got the difference between and like people who sell fruit and veg they don't need like a COA or business license and people who are selling like clothing and shoes and non food items they – but where they stand they do need the permit based on the area that they are based.

______________________________________________________________________

Code: Minister of health {2-0}

P 2: Meeting EHManagers.doc - 2:68 [No, no Vanesh[?] let me defend..]  (152:152)   (Super)
Codes:	[Minister of health] 
No memos

No, no Vanesh[?] let me defend other I don't know Vanesh if you have got enough help, but the Minister of Health he's always advocating for healthy lifestyles and there is even new regulations now 

P 2: Meeting EHManagers.doc - 2:69 [But still, the minster is not ..]  (154:154)   (Super)
Codes:	[Consumer education] [Minister of health] 
No memos

But still, the minster is not going to talk about the five keys of health and the labeling and stuff, we need the ground level thing not on a big national radio there local radios playing in this area that area local where they understand the things.

______________________________________________________________________

Code: Mobile cart business example {2-0}

P 3: Meeting_XXXXX.doc - 3:49 [Yes, he has now moved he is no..]  (89:89)   (Super)
Codes:	[Cart_example] [Mobile cart business example] 
No memos

Yes, he has now moved he is now down in Ottery somewhere but the model there is that he will fund the building of that unit and then every morning he would stock the unit so I don't know how many he has at the moment, but I think about ten or 20, he will stock the units and then deliver it to the roadside place and then that is where the vendor will be selling and it's fresh fish and his whole business model it is healthy fish it is not old fish and it picks up your passing trade and he's trying first of all he's trying not to compete with your typical fish vendor from the Cape Flats he is trying to create an opportunity for new vendors to be on the Atlantic Seaboard than the posh areas. But, I don't know if that is what you are looking at because this is fresh fish that you have to take home to cook this is not selling food on the pavement which you can eat straight away.

P 3: Meeting_XXXXX.doc - 3:50 [Because to me you now mention ..]  (92:92)   (Super)
Codes:	[Mobile cart business example] 
No memos

Because to me you now mention that okay he has chosen fish because that is the business that he is in but what happened if somebody like that taking the Bangkok food and say that I am going to provide the unit and stir fry vegetables and distribute them all over the municipal area.

______________________________________________________________________

Code: Mobile facility {2-0}

P 2: Meeting EHManagers.doc - 2:13 [So your whole plan and whole t..]  (40:40)   (Super)
Codes:	[Mobile facility] 
No memos

So your whole plan and whole thing is around a mobile facility as well?

P 2: Meeting EHManagers.doc - 2:14 [You work with the mobile facil..]  (42:42)   (Super)
Codes:	[Mobile facility] 
No memos

You work with the mobile facility and then with the food and nutritious side of it but it's around a little hawkers vehiclekie[?] caravankie[?] or whatever it is.

______________________________________________________________________

Code: Night trade {1-0}

P 4: SF Workshop_transcript.docx - 4:35 [When Janice comes back she dea..]  (99:99)   (Super)
Codes:	[Night trade] 
No memos

When Janice comes back she deals with night trading hey. There you have a nice set up there probably, I haven't seen it, but I am sure they have sophisticated stuff. for them to actually go out and see what's in the night trade. 

______________________________________________________________________

Code: No qouta for licences/COA {1-0}

P 1: Interview with XXXXX.docx - 1:61 [No. we don't have a quota. So ..]  (134:134)   (Super)
Codes:	[No qouta for licences/COA] 
No memos

No. we don't have a quota. So people that are interested in earning a living, apply for the license and comply with the requirements in terms of food safety, we basically issue those licenses. 

______________________________________________________________________

Code: Non-trading zone {3-0}

P 2: Meeting EHManagers.doc - 2:17 [so most of them most of those ..]  (44:44)   (Super)
Codes:	[Non-trading zone] [Traffic bylaw] 
No memos

so most of them most of those vendors the ones that are braaing the meat along the roadside they are not supposed to be there because those areas are not even zoned for economic purposes they are not for commercial purposes so they just forcing matters by being there and if things were to go the city's way the city could just remove all of them tomorrow or as in yesterday. So maybe that is why the colleagues from ED were kind of like cautious a bit skeptical towards… 

P 2: Meeting EHManagers.doc - 2:18 [Because now if we maybe the ci..]  (44:44)   (Super)
Codes:	[Non-trading zone] 
No memos

Because now if we maybe the city would be very cautious like now they sort of granting them permission it's like they legalizing they are being there yet that space is not for trading purposes because there are certain areas that are zoned for whatever trade. 

P 2: Meeting EHManagers.doc - 2:19 [But, then there is also a very..]  (44:44)   (Super)
Codes:	[Non-trading zone] 
No memos

But, then there is also a very another predicament where city would maybe zone certain areas for trading purposes but because the vendors themselves feel that the traffic it is outside the area that is zoned for that the city regards to be suitable for the trade that they are in is not so safe to them because maybe good for the customers coming from the train and going back home or going to catch a bus or a taxi or something so it is quite it is too much many components in it involved

______________________________________________________________________

Code: Not applicable legislation {7-0}

P 1: Interview with XXXXX.docx - 1:17 [The international health regul..]  (64:64)   (Super)
Codes:	[Not applicable legislation] 
No memos

The international health regulations act, hell, I don't think so. [Let's make a little cross there]. 

P 1: Interview with XXXXX.docx - 1:18 [No look, I think if you want t..]  (68:68)   (Super)
Codes:	[Not applicable legislation] 
No memos

No look, I think if you want to make a vendor aware of the agriculture products act, unless they want to make fruit juices on the cart and things like that that may be an issue, so let's leave that one in. the liquor products act, they not going to sell alcohol. Not applicable. 

P 1: Interview with XXXXX.docx - 1:19 [Then I think it is fine, one d..]  (71:71)   (Super)
Codes:	[Not applicable legislation] 
No memos

Then I think it is fine, one don't actually have to go into too much details if you deal with traders and you want to teach them these things and so on. I think what is important for them is to know that the food that they purchase come from a safe source. If they buy meat it should come from a safe source. Ok. So the abattoir hygiene act is not going to influence them.

P 1: Interview with XXXXX.docx - 1:21 [Same thing with the animal dis..]  (71:71)   (Super)
Codes:	[Not applicable legislation] 
No memos

Same thing with the animal diseases act I don't think your trader that is going to run a little cart like that would need an in depth knowledge about this.

P 1: Interview with XXXXX.docx - 1:22 [The same with the farm feeds a..]  (71:71)   (Super)
Codes:	[Not applicable legislation] 
No memos

The same with the farm feeds and the agriculture products act, stop remedies act, I don't think that is applicable. 

P 1: Interview with XXXXX.docx - 1:25 [The standards act, I don't eve..]  (71:71)   (Super)
Codes:	[Not applicable legislation] 
No memos

The standards act, I don't even know under which that falls. No idea. Plant breeder's rights act, no! Really. Agricultural pest act, no. Trade metrology, maybe if they are selling things in weights and things like that if they put a weight on something, but in traditional terms you would not.

P 1: Interview with XXXXX.docx - 1:26 [That is what the trade metrolo..]  (73:73)   (Super)
Codes:	[Not applicable legislation] 
No memos

That is what the trade metrology act becomes complicated for the people.

______________________________________________________________________

Code: Now, one of the challenges tha.. {0-0}
______________________________________________________________________

Code: Occasional/seasonal trade {4-0}

P 1: Interview with XXXXX.docx - 1:63 [Ja look, I think that is the r..]  (145:145)   (Super)
Codes:	[Occasional/seasonal trade] 
No memos

Ja look, I think that is the reality unfortunately. Because some of the constraints that we have, that the colleagues find is the fact that you have a very seasonal thing, and you have a very occasional sense. Occasional in the sense of urhm, with pension day, although with the SASSA thing I am not sure how that pans out now. End of month things, end of week things, lots of people actually operate on a Friday, Saturday, Sunday and things like that. And they spring up, some may operate for 4,5, 6 weeks or two months and things like that and then all of a sudden they are no longer there, or whatever the case may be.

P 1: Interview with XXXXX.docx - 1:65 [But it is also as I say people..]  (146:146)   (Super)
Codes:	[Occasional/seasonal trade] 
No memos

 But it is also as I say people spring up and the city is big you know and we have about, what 140 operational people.

P 2: Meeting EHManagers.doc - 2:46 [We seem to get more traders on..]  (98:98)   (Super)
Codes:	[Occasional/seasonal trade] 
No memos

We seem to get more traders on that day everywhere where there is pay days and those people after that they disappear we don't see them again.

P 2: Meeting EHManagers.doc - 2:47 [When you say you've got a scho..]  (100:100)   (Super)
Codes:	[Occasional/seasonal trade] 
No memos

When you say you've got a school that's got one trader it is very questionable but if you have a pay day we have more and more numbers on them.

______________________________________________________________________

Code: Oil usage {3-0}

P 2: Meeting EHManagers.doc - 2:84 [The other thing depending on w..]  (205:205)   (Super)
Codes:	[Oil usage] 
No memos

The other thing depending on what the person – you looking at a healthier way which I suppose excludes oil?

P 2: Meeting EHManagers.doc - 2:85 [Quality of oil yes that's good..]  (211:211)   (Super)
Codes:	[Oil usage] 
No memos

Quality of oil yes that's good because there is a color chart but I guess that goes with the education. There is a color chart that we use…

P 2: Meeting EHManagers.doc - 2:86 [Because you find that – you do..]  (213:213)   (Super)
Codes:	[Oil usage] 
No memos

Because you find that – you don't find it in the informal only, you find it in the formal as well where they reuse the oil.

______________________________________________________________________

Code: One size fits all not pratical {1-0}

P 4: SF Workshop_transcript.docx - 4:34 [Most important is the point yo..]  (97:97)   (Super)
Codes:	[One size fits all not pratical] 
No memos

Most important is the point you made earlier on, you cannot have one size fits all because we have a range of different food vendors. From fruit and veg, to tikka chicken, chips and chocolate...the breyanis, the akhnis. So it is so that you cannot have a one size fits all for the food industry and that is very important from the onset to have in mind.

______________________________________________________________________

Code: Penalty for non-payment_permit {2-0}

P 3: Meeting_XXXXX.doc - 3:66 [We have two penalties the one ..]  (117:117)   (Super)
Codes:	[Penalty for non-payment_permit] 
No memos

We have two penalties the one just in terms of location permit we generally give people three months to rectify and that is to prove that we can't be hands on every minute of the day, the system is not set up that when you don't pay there is a red flag that says Paul hasn't paid but we do check and then if the person hasn't paid the second month we fill follow up, what's the problem. We try and encourage the vendor to have healthy relationship with the district person, so maybe it's a case of financial crisis or whatever. And then we would try and support that vendor to ultimately pay the arrears and to continue paying. The second penalty is something we don't have any control over the law enforcement officer…

P 3: Meeting_XXXXX.doc - 3:67 [Here's my permit. No, that exp..]  (121:121)   (Super)
Codes:	[Penalty for non-payment_permit] 
No memos

Here's my permit. No, that expired two months ago. Now what law enforcement does and it has a lot of discuss it with us first of all they say you a bad person go and see the area coordinator and sort it out and we will not do – what they can do, they can impound your goods but they don't do because you've got a permit the fact that the permit expired sort it out we will give you a fine, or maybe they will give you a verbal warning the second time it is a fine. But, they are a bit more lenient with people who have permits even though the permit has expired.

______________________________________________________________________

Code: Penalty_no permit {1-0}

P 3: Meeting_XXXXX.doc - 3:68 [If it is someone who hasn't go..]  (121:121)   (Super)
Codes:	[Penalty_no permit] 
No memos

If it is someone who hasn't got a permit in a prohibited area, then the law enforcement is a bit more harsh then it's a case of pack up, I take your stuff away, if you don't do it we will impound your goods. 

______________________________________________________________________

Code: Permit {15-0}

P 1: Interview with XXXXX.docx - 1:43 [he permit is the location. Tha..]  (97:97)   (Super)
Codes:	[Permit] 
No memos

he permit is the location. That is what they pay the city for to stand there and things like that. 

P 1: Interview with XXXXX.docx - 1:44 [No we do not give temporary pe..]  (98:98)   (Super)
Codes:	[Permit] 
No memos

No we do not give temporary permits. 

P 2: Meeting EHManagers.doc - 2:29 [he other thing that I will sen..]  (55:55)   (Super)
Codes:	[Business license] [Permit] 
No memos

he other thing that I will send you is the Business License Act so those are the two pieces of legislation that we would look at. Johan has said that besides even if you have both of those legislations or you… Even if you have the certificate and the business license it still doesn't allow you to trade in certain areas because that is where the permit system comes in so if you have – 

P 2: Meeting EHManagers.doc - 2:31 [for example Bellville CBD ther..]  (55:55)   (Super)
Codes:	[Permit] 
No memos

for example Bellville CBD there are allocated zones where people are allocated areas where people can and that is a permit system in order words you have to apply for it you have to pay for it and they have certain rules.

P 2: Meeting EHManagers.doc - 2:32 [I think the permit thing more ..]  (58:58)   (Super)
Codes:	[Permit] 
No memos

I think the permit thing more is you've got to have is like anything else. You've got to have the owner's permission to be on the property that is insured because if it is like in the areas that they said areas allocated or not, that is the city of Cape Town's property. 

P 2: Meeting EHManagers.doc - 2:34 [And their permission is what w..]  (58:58)   (Super)
Codes:	[Permit] 
No memos

And their permission is what we are talking about a permit because a permit is not always a piece of paper but sometimes it is where it is an allocated place where there are toilets and stuff like that and they give you a permit and you apply for a permit from the owner of the property which is 90% times the city. But, the permit that is what I always explain to the people, the permit is the…

P 3: Meeting_XXXXX.doc - 3:51 [There are two components the o..]  (98:98)   (Super)
Codes:	[Certificate of acceptability] [Permit] 
No memos

There are two components the one is what I call a location permit and the other one deals with your health certificate I'm going to use the broad word health certificate. 

P 3: Meeting_XXXXX.doc - 3:52 [Now at the moment, if you were..]  (98:98)   (Super)
Codes:	[Allocated vs non-allocated zones] [Permit] 
No memos

Now at the moment, if you were to look at the Cape Town municipal area roughly 60% of the entire municipal area is an area where you need a location permit so take a silly example which Mitchell's plain town centre if you want to take there you need to get permission to trade in that yellow block and if you don't have a permit you can't trade so that is the one side of it. And by the way, the other 40% is effectively area where we don't need a location permit so as long as you comply with bylaws you can go and stand. So again using a silly example if you are AZ Berman Drive of Mitchells Plan you can stand alongside the road you don't need permission.

P 3: Meeting_XXXXX.doc - 3:55 [I use the word location permit..]  (102:102)   (Super)
Codes:	[Permit] 
No memos

I use the word location permit because it makes sense. And then what I tend to do with the vendors is I put them in touch with my eight districts because each district is different so for example Richard looks after Mitchells Plain very quickly if you want to take there are two areas we need a location certificate – location permit is the town centre and Lentegeur[?] anywhere else in Mitchells Plain you don't need a location permit

P 3: Meeting_XXXXX.doc - 3:59 [There is a movement to give it..]  (104:104)   (Super)
Codes:	[Permit] 
No memos

There is a movement to give it a lifespan and I will talk about that but right now if you get a permit to trade in how many square Mitchell Plain's town centre as long as you comply with city bylaws and behave yourself and as long as you pay for that permit every month.

P 3: Meeting_XXXXX.doc - 3:60 [It ranges we just had a… It's ..]  (106:106)   (Super)
Codes:	[Permit] 
No memos

It ranges we just had a… It's a monthly fee and I think the bottom end is R60 a month depending on the area and the top end is R400 but that is just purely a location…

P 3: Meeting_XXXXX.doc - 3:61 [I'm now waiting for the inform..]  (108:108)   (Super)
Codes:	[Permit] 
No memos

I'm now waiting for the informal economy to start putting political pressure on the city again because to me the location permit is not about money for the city but yet there is a huge element of the city where income helps in terms of your fiscus so it's…

P 3: Meeting_XXXXX.doc - 3:63 [So now again, there is pressur..]  (110:110)   (Super)
Codes:	[Permit] 
No memos

 So now again, there is pressure to try and say, we need to increase the informal trading fees so that you can cross pollinate the law enforcement. There is whole lot of debate about that but to me the location permit is not about making money sorry what was your question how much… And it is valid you pay for it every month but there is no cut off period. If one of them moves a foot within the city is to explore increasing that fee so just say you trading at town centre and it's R80 a month after a year we then push it up to maybe R100/R160…

P 3: Meeting_XXXXX.doc - 3:64 [And then after two years so yo..]  (112:112)   (Super)
Codes:	[Permit] 
No memos

And then after two years so you have an increment in your increase so that it in a sense forces you to look at other areas to go out.

P 3: Meeting_XXXXX.doc - 3:69 [And then the second component ..]  (121:121)   (Super)
Codes:	[Permit] 
No memos

And then the second component apart from the increase in the tariff what we are considering and exploring is saying that the permit should be valid for a period of a year or two or three and then after that you need to reapply and that is to create opportunity for new entrance, but again it's a difficult area to, reality and…

______________________________________________________________________

Code: Policies vs legislation {3-0}

P 1: Interview with XXXXX.docx - 1:13 [Now this is not policies madam..]  (56:56)   (Super)
Codes:	[Policies vs legislation] 
No memos

Now this is not policies madam this is legislation. Policies are a little different to legislation. Policies you cannot enforce, policies give strategic direction and urhm whereas regulation you have to comply with it. It is enforceable. 

P 1: Interview with XXXXX.docx - 1:14 [A policy is a guide. So what y..]  (60:60)   (Super)
Codes:	[Policies vs legislation] 
No memos

 A policy is a guide. So what you will do initially is you will provide an overall policy, to send you the direction and to tell the people what it is that you want to do, and then in order to fulfil that policy you will then develop legislation. 

P 3: Meeting_XXXXX.doc - 3:10 [Let me finish talking about… T..]  (35:35)   (Super)
Codes:	[Bylaws of the city] [Policies vs legislation] [Various departments involved] 
No memos

Let me finish talking about… The city has got a policy its got its bylaw and what we did in terms of both the document took very much into account the 1991 document, so the backbone to our policies and bylaws and then also took into account other legislations around for example food trading. So when it comes to environmental health aspects and more specifically street trading and food, we don't specify in our bylaw just to say that any other bylaw that exists or any other legislation you need to comply with it and we had a huge debate in one sense and if you think from an informal trader perspective they don't want to have to go and search the entire legislative framework to find out what is applicable to them. And then the city we don't want to necessarily take one document and pull in from every other so you got environmental health legislation, you've got this legislation to do with solid waste your cleansing, you got traffic bylaws and all that that impacts on informal economy because then when you change your environmental health legislation you've got to remember you've got to change your informal trading bylaws so it was a debate and I don't think we settled that finally so we have said, Christa will talk about all the environmental health aspects and that embodied in their policies and their legislation. In the city's policy and bylaws we have just made reference to that you need to be aware of health issues and health and sanitation we don't specify…

______________________________________________________________________

Code: Presciptiveness as a strategy? {2-0}

P 3: Meeting_XXXXX.doc - 3:15 [You see to me I mean I have tw..]  (41:41)   (Super)
Codes:	[Presciptiveness as a strategy?] 
No memos

You see to me I mean I have two tensions the one is first of all is that national legislature says that you not allowed to be prescriptive, so the city is now being prescriptive and to me I take it but again, for me sitting here and saying sushi is a healthy food but if I go into townships to sell sushi it is not going to work.

P 3: Meeting_XXXXX.doc - 3:28 [So you can go to Bellville tra..]  (50:50)   (Super)
Codes:	[Presciptiveness as a strategy?] 
No memos

So you can go to Bellville transport interchange and say in this area fruit and vegetables can be sold or people can't eat fish you got to take it somewhere to be cooked, but you won't allow the frying of food but will that cause people who normally buy fatty food to then go and buy fruit and vegetables.

______________________________________________________________________

Code: Private storage arrangements {1-0}

P 4: SF Workshop_transcript.docx - 4:10 [The question of storage, it is..]  (13:13)   (Super)
Codes:	[Private storage arrangements] 
No memos

The question of storage, it is personal/private storage arrangements. Most traders have engaged business for their private storing purposes. So they have to get out by a certain time, as obviously businesses must trade, so by 6/7am. And then they must be in at a certain time as the business close for the day. Even though there might still be “traffic” in Wynberg, Claremont, they then lose out on that business. 

______________________________________________________________________

Code: Property owner's permission {1-0}

P 2: Meeting EHManagers.doc - 2:33 [I think the permit thing more ..]  (58:58)   (Super)
Codes:	[Property owner's permission] 
No memos

I think the permit thing more is you've got to have is like anything else. You've got to have the owner's permission to be on the property that is insured because if it is like in the areas that they said areas allocated or not, that is the city of Cape Town's property. If I want to go and stand at the hypermarket or Shoprite then the permit will be it's not necessarily that the permit is a piece of paper, but you've got to have permission of the owner of that not necessarily Checkers but the management centre or the management or whatever it is, the owner of that property you are standing on because it is private property it belongs to a company, but you need their permission. 

______________________________________________________________________

Code: R962 {7-0}

P 1: Interview with XXXXX.docx - 1:45 [What are the key factors in gr..]  (99:99)   (Super)
Codes:	[R962] 
No memos

What are the key factors in granting a certificate? That I think those are the things I'll send on to you or to Paul or things like that. But it goes around R962, which would be the requirements for a food premises. And things like that ok. 

P 1: Interview with XXXXX.docx - 1:46 [Now R962 is the regulation tha..]  (100:100)   (Super)
Codes:	[R962] 
No memos

Now R962 is the regulation that deals with these things and is promulgated from the foodstuffs act. So that's how these things fall into play. 

P 2: Meeting EHManagers.doc - 2:15 [I think the cart of Cape Winel..]  (44:44)   (Super)
Codes:	[Cart requirements] [Cart_example] [R962] 
No memos

I think the cart of Cape Winelands will bring with it some answers to what you are looking for because they have a safe component in it there is I can't remember if it is a tap or 25 litre water container – clean water thing in whereby one can use the sink maybe to wash maybe your vegetables or whatever or the meat or anything and then at the bottom there is also an empty 25 litre container to drain the thing actually. So it's a typical – an ideal model that really satisfies us as EHPs because its got all the components of what we are looking for and then I think it also has a space for them to label their stalls because our regulations are R962 requires that if it is shop that you are selling then it must be clearly labeled with your name and everything.

P 2: Meeting EHManagers.doc - 2:22 [R962 that's the main one that ..]  (46:46)   (Super)
Codes:	[R962] 
No memos

R962 that's the main one that the Bible. 

P 2: Meeting EHManagers.doc - 2:27 [Your main piece legislation wo..]  (51:51)   (Super)
Codes:	[R962] 
No memos

Your main piece legislation would be R962 which I can forward to you now if you give me the email address.

P 2: Meeting EHManagers.doc - 2:28 [I will send it to you now. But..]  (55:55)   (Super)
Codes:	[Certificate of acceptability] [R962] 
No memos

I will send it to you now. But I mean like Johan was saying it covers the definitions, it covers Certificate of Acceptability which one of the things that you asked about certification that is required. 

P 2: Meeting EHManagers.doc - 2:49 [But, please I don't know I wou..]  (104:104)   (Super)
Codes:	[5 keys to safer foods] [R962] [R962 diagram] 
No memos

 But, please I don't know I would not want you to leave it out now that we've got this ideal cut work space, how the work space should like and then we've got the five keys let us also have him or her to say that this is how you as a food vendor should look like so that we've got a full picture. And then probably also if in this cart you could also maybe have the space where they could maybe later [unclear 0:40:25] something there where they would – their name could be so that even if somebody a customer comes then they buy something then he or she gets food poisoning then she can quickly tell us that she got it from Zandile Sparsa shop you work from Zandile's braai stand and then you know that there is only one Zandile braai stand there and that would also make us fully compliant in terms of R962 because those are the type of other things that are needed.

______________________________________________________________________

Code: R962 diagram {2-0}

P 2: Meeting EHManagers.doc - 2:48 [we have a diagram where that w..]  (104:104)   (Super)
Codes:	[R962 diagram] 
No memos

we have a diagram where that we usually give to the food vendors when we do when we workshop them around hygiene requirements in terms of R962 what is expected in terms of their food handling and how their premises should be and something like that. So we got that like a sketch diagram of like a typical or our ideal food vendor how you or she should look like. So in there we've now this person wearing a clean apron, wearing a head cover, having a 25 litre container of clean water and the one for waste water as well as a dirt bin for the collection of where he or she could collect the refuge so we've got that. 

P 2: Meeting EHManagers.doc - 2:49 [But, please I don't know I wou..]  (104:104)   (Super)
Codes:	[5 keys to safer foods] [R962] [R962 diagram] 
No memos

 But, please I don't know I would not want you to leave it out now that we've got this ideal cut work space, how the work space should like and then we've got the five keys let us also have him or her to say that this is how you as a food vendor should look like so that we've got a full picture. And then probably also if in this cart you could also maybe have the space where they could maybe later [unclear 0:40:25] something there where they would – their name could be so that even if somebody a customer comes then they buy something then he or she gets food poisoning then she can quickly tell us that she got it from Zandile Sparsa shop you work from Zandile's braai stand and then you know that there is only one Zandile braai stand there and that would also make us fully compliant in terms of R962 because those are the type of other things that are needed.

______________________________________________________________________

Code: R962_non-compliance {1-0}

P 2: Meeting EHManagers.doc - 2:20 [I think that is why in a way c..]  (44:44)   (Super)
Codes:	[R962_non-compliance] 
No memos

I think that is why in a way city would like to try it very carefully but then those people are there and they are not complying to the regulations and what do we do because that is now the question that we are all sitting with they are there right now they are servicing our communities they not complying like we found that they not even hand washing and other things that are compromising with the health of the consumers so what we going to do. 

______________________________________________________________________

Code: Revision of policy + bylaws {1-0}

P 3: Meeting_XXXXX.doc - 3:12 [And then from that summit the ..]  (37:37)   (Super)
Codes:	[Informal trading summit] [Revision of policy + bylaws] 
No memos

And then from that summit the city has revised its policy and bylaws, so I can give you copies of that. But, again, it wasn't focusing on food and health specifically more a generic document to say this is how we want to grow and enhance the sector. 

______________________________________________________________________

Code: Satiety factor {2-0}

P 2: Meeting EHManagers.doc - 2:77 [And like I said before it's a ..]  (174:174)   (Super)
Codes:	[Satiety factor] 
No memos

And like I said before it's a filling up factor that plays a role.

P 2: Meeting EHManagers.doc - 2:78 [If I feel my stomach is filled..]  (176:176)   (Super)
Codes:	[Satiety factor] 
No memos

If I feel my stomach is filled up I feel I am okay for the day I can… But it impacts on your concentration and all those things. 

______________________________________________________________________

Code: Security of tender {1-0}

P 3: Meeting_XXXXX.doc - 3:38 [The other component of a tradi..]  (75:75)   (Super)
Codes:	[Security of tender] 
No memos

The other component of a trading plan is often with traders they want security of tender so when they get up in the morning they go to their spot they want to know that that spot is theirs that they don't get there and they find that somebody else there. And to be controversial a local trader gets there and then there is suddenly a foreign national there then there is a war going on or somebody else. 

______________________________________________________________________

Code: Security_crime_theft {1-0}

P 3: Meeting_XXXXX.doc - 3:94 [What we try and do there is wh..]  (176:176)   (Super)
Codes:	[Security_crime_theft] [Vendor challenges] 
No memos

What we try and do there is where there are existing municipal facilities I use the word broadly, but for example where you've got a transport interchange, in any case at the facility there is security to look after the facility so they also address the crime and crime issue. In your established areas there are city improvement district or special rating areas where businesses contribute towards people that look after crime and grime but it is a real challenge and then the other thing that you haven't said there is a perception amongst to some communities that traders are directly responsible for the criminal element they are front for drugs, they fronts for money laundering etcetera, etcetera. They also are the eyes and ears of ATMs and they provide information and all that sort of thing. In some cases it does happen we know it happens but to a large extent perceptions of the community is not based on reality.

______________________________________________________________________

Code: SF project vision/mission_complementary {1-0}

P 1: Interview with XXXXX.docx - 1:55 [And would the aims of this, th..]  (116:116)   (Super)
Codes:	[SF project vision/mission_complementary] 
No memos

And would the aims of this, this would now be this SFVBM of yours? …with this vision? Well I think any improvement in how people first of all deal with their businesses, if it is a healthier product, it will fit in overall on a national basis not just what we as a city subscribe to for healthy lifestyles and things like that and so on, with the diseases burden that you have. I think anything that fits in with that would be welcomed. I mean we cannot, we will not be able to criticize it, let me put it that way. 

______________________________________________________________________

Code: Site visits {2-0}

P 4: SF Workshop_transcript.docx - 4:13 [Maybe onsite visits to see how..]  (24:24)   (Super)
Codes:	[Site visits] [Vendor challenges] 
No memos

Maybe onsite visits to see how it works. At the moment we deal with historical traders who have ties with businesses. New comers struggle a bit in terms of storage etc.  

P 4: SF Workshop_transcript.docx - 4:16 [Then you get individuals in CP..]  (26:26)   (Super)
Codes:	[Site visits] 
No memos

Then you get individuals in CPT centre we moved them into kiosks with all their trims and fittings. So you could go look at them for example but for me it is the basic skottelbraai. So anything would be an improvement from my side. So something like this I would welcome it. 

______________________________________________________________________

Code: Storage_challenge {7-0}

P 2: Meeting EHManagers.doc - 2:12 [Now, my second question the st..]  (37:37)   (Super)
Codes:	[Storage_challenge] 
No memos

Now, my second question the storage of this it's easy to design something when people are complaining about crime and your safety issues is it breakable…

P 3: Meeting_XXXXX.doc - 3:84 [I think one has to distinguish..]  (151:151)   (Super)
Codes:	[M'Plain Towncentre] [Storage_challenge] 
No memos

I think one has to distinguish between the Mitchells Plain town centre as a city wide model but you don't know in Mitchells Plain as the part of the whole discussion I don't know if you know of the model there, but really of an old bus terminus and effectively what we did we created a market underneath that terminus so it is an undercover market under roof market and then along the edges you've got kiosks and you have different size kiosks, you have large kiosks and you have small kiosks. But, the bulk of the not the bulk but the sizeable number of traders in the town centre are fruit and vegetable traders but there are also some who are not fruit and vegetable traders and the question is you know, when your means of transport is a mini bus taxi, what do you do with your goods at the end of the day.

P 3: Meeting_XXXXX.doc - 3:85 [So it's intensive but certainl..]  (155:155)   (Super)
Codes:	[CCT's infrastructure_challenge] [Storage_challenge] 
No memos

So it's intensive but certainly because of the historical negotiations took place with the town centre the issue of storage facilities is on that agenda there. City wise it causes a tension because first of all it's just city it's a municipality and business are providing storage facilities for traders and if your answer is yes are the traders prepared to pay for that and often you find that the traders are not prepared to pay for it.

P 3: Meeting_XXXXX.doc - 3:86 [But then the flip side of the ..]  (155:155)   (Super)
Codes:	[Storage_challenge] 
No memos

But then the flip side of the coin is that if you look at anyone of the established areas just taking Bellville as an example or Cape Town Claremount, Wynberg, traders do store their goods and they make their own arrangement with property owners in some cases they use parking areas, parking garages in other cases you must know about it but the fire escapes and they do store there.

P 4: SF Workshop_transcript.docx - 4:1 [Irrespective of the design, th..]  (6:6)   (Super)
Codes:	[Storage_challenge] 
No memos

 Irrespective of the design, the issue in my area is storage. I have a situation now where a trader stores her goods in a shop….

P 4: SF Workshop_transcript.docx - 4:2 [The starting point should be w..]  (7:7)   (Super)
Codes:	[Storage_challenge] 
No memos

The starting point should be where the unit will be stored. Irrespective if it is mobile or not. 

P 4: SF Workshop_transcript.docx - 4:3 [How is it going to be brought ..]  (7:7)   (Super)
Codes:	[Storage_challenge] [Transporting cart] 
No memos

How is it going to be brought to the trading space? In some cases people come via public transport. So they cannot travel with the unit plus other commodities with public transport. Storage is a huge issue. So if the storage can be solved a mobile unit could work.

______________________________________________________________________

Code: Support_city/municipality {1-0}

P 1: Interview with XXXXX.docx - 1:54 [Support available to the vendo..]  (114:114)   (Super)
Codes:	[Support_city/municipality] 
No memos

Support available to the vendors available from the municipality or … Well I think the support because the guys do the visit, they do the inspection at the stall from time to time, we do take samples from them from time to time. And that would be our support from our side. If we get complaints from the members of the public about the way they conduct their business and things like that or food quality or safety quality, then we will interact with them or so on. But much like the restaurant you know we give them the same kind of attention and things like that. 

______________________________________________________________________

Code: Sustainability {1-0}

P 4: SF Workshop_transcript.docx - 4:21 [But then the cost of that unit..]  (37:37)   (Super)
Codes:	[Sustainability] 
No memos

But then the cost of that unit. I would be interested, Bruce has only been around for a year now, with the mobile vending unit. And I do not know how sustainable it is. He was hoping to roll out in the Deep South but it never happened. 

______________________________________________________________________

Code: TCP programme {5-0}

P 1: Interview with XXXXX.docx - 1:1 [Good heavens it was twelve yea..]  (17:17)   (Super)
Codes:	[TCP programme] 
No memos

Good heavens it was twelve years ago. 

P 1: Interview with XXXXX.docx - 1:2 [We did something similar in 19..]  (19:19)   (Super)
Codes:	[TCP programme] 
No memos

 We did something similar in 1997 and then there was a follow-up with this. Where I think the informal traders were divided into 5 different zones so to speak, at transport hubs and very informal areas and things like that. I actually have forgotten about all of that. 

P 1: Interview with XXXXX.docx - 1:3 [I do not think that this is co..]  (21:21)   (Super)
Codes:	[TCP programme] 
No memos

I do not think that this is copyrighted. This has been documented and the department of health and FAO so I don't think…

P 1: Interview with XXXXX.docx - 1:4 [So they would be happy if we t..]  (22:22)   (Super)
Codes:	[TCP programme] 
No memos

So they would be happy if we take it on. But I can maybe advise you to contact Penny Campbell in Pretoria she is the acting director at the moment for food control. 

P 3: Meeting_XXXXX.doc - 3:3 [the Durban conference I do not..]  (13:13)   (Super)
Codes:	[TCP programme] 
No memos

 the Durban conference I do not know about.

______________________________________________________________________

Code: Temperature control {2-0}

P 1: Interview with XXXXX.docx - 1:50 [But that is why we accept the ..]  (109:109)   (Super)
Codes:	[COA_requirements] [Temperature control] 
No memos

But that is why we accept the cooler boxes, and so on. So if your product goes out of your home in the morning at 4 degrees Celsius, you have ice boxes and coolerbags and things like that, you can maintain it at a safe temperature, unless you are going to leave that bin open or that container open, or you going to open and close, open and close it, and things like that. So that is why I say that business model is very important. 

P 1: Interview with XXXXX.docx - 1:51 [And then of course keeping thi..]  (110:110)   (Super)
Codes:	[Temperature control] 
No memos

And then of course keeping things hot that is meant to be hot. Don't make the sausage 10am this morning then it sits outside room temperature, 28 degrees in the centre of town and you only warming it up again to put it on a roll or something like that you know. Or salads and things like that and so on. 

______________________________________________________________________

Code: Territorial wars {3-0}

P 3: Meeting_XXXXX.doc - 3:39 [And to be controversial a loca..]  (75:75)   (Super)
Codes:	[Territorial wars] 
No memos

And to be controversial a local trader gets there and then there is suddenly a foreign national there then there is a war going on or somebody else. 

P 3: Meeting_XXXXX.doc - 3:40 [It will be more controversial ..]  (75:75)   (Super)
Codes:	[Territorial wars] 
No memos

It will be more controversial and I am now discussing in Langa – the Langa community saying we only want the people from Langa selling. So okay, let's just take that debate a bit further so in Cape Town CBD there shouldn't be any informal traders from Langa in Cape Town CBD they must go back to Langa and trade and then people got upset with me.

P 3: Meeting_XXXXX.doc - 3:41 [But then I say, it is all very..]  (75:75)   (Super)
Codes:	[Territorial wars] 
No memos

But then I say, it is all very well to sit there and say in my community I only want people from my community that's fine, I understand it but how far do you stretch that. 

______________________________________________________________________

Code: Thailand SFM as example {1-0}

P 3: Meeting_XXXXX.doc - 3:30 ['m just thiking… One cannot co..]  (52:52)   (Super)
Codes:	[Thailand SFM as example] 
No memos

'm just thiking… One cannot compare the two I spend some time in Thailand and I've got a good friend she is now in South America but she says seven days a week of those seven days she probably will cook a meal maybe one or two days in her apartment the other times she goes down onto the street and she buys either precooked and then takes it up and what they are selling on the streets there is actually very healthy it's stir fry and all that sort of thing. Now, I tried to project[?] that sort of Bangkok street would that work in Bellville, Cape Town I don't know.

______________________________________________________________________

Code: Trading from home {1-0}

P 1: Interview with XXXXX.docx - 1:62 [Yes and no. if somebody trades..]  (136:136)   (Super)
Codes:	[Trading from home] 
No memos

Yes and no. if somebody trades from their home, that might be a different license. Urhm it may actually need to go to land use to see that because we have to be careful with trading from home. The context, the volume, the type of trading and things like that and that would not be seen as a hawker anymore. That would definitely be seen as for instance a supplier of a meal, in terms of a formal process. Because it is now a formal premises. Because we view informal as things with non-permanent structures. The moment you have a permanent structure and things, we start to view it as a more formal premises. So that is more of a different approach and then land use, first priority is for land use to say yes, in terms of the schemes and things like that. You are allowed to have a home industry and things like that. Then it goes to Fire, Building inspectors and to Health. There are four reporting officials on a formal license. Whereas the trading hawkers license, it is just two. 

______________________________________________________________________

Code: Trading plans {5-0}

P 3: Meeting_XXXXX.doc - 3:36 [But, to me one would think jus..]  (75:75)   (Super)
Codes:	[Trading plans] 
No memos

But, to me one would think just getting back to what the city's do we create trading plans so we take an area and there are a number of different aspects of a trading plan, but we take the area and we say this is where people can trade, this is where you can't trade and then in terms of our bylaw we can actually be prescriptive in what you can trade in and what hours you can trade. 

P 3: Meeting_XXXXX.doc - 3:37 [We haven't used it yet, and I'..]  (75:75)   (Super)
Codes:	[Trading plans] 
No memos

We haven't used it yet, and I'm skirting[?] away from it because I think we can be a big challenge and our political representative in the communities the ward councilors they haven't pushed only one area but it didn't have to do with food but strange enough it had to do with a wood seller saying that he should only trade sell wood in daylight hours not after hours after… But the mechanism is there that the city can say in this area there are ten traders of those ten five can sell food, five can't sell food and we can do that. 

P 3: Meeting_XXXXX.doc - 3:42 [The other side of the trading ..]  (75:75)   (Super)
Codes:	[Trading plans] 
No memos

The other side of the trading plan comes down more to the regulatory side is where in more and more of our areas the community want to know the informal trading is regulated in terms of where they trade and then the commodities they trade and there is an element now coming through more of the nutritional healthy food it's still very low key not in your face, but it is coming through. And then in the city of Cape Town what the political push is that the entire municipal area an area where if you don't have a permit you can't trade now to me that is going back to the pre 91 era you know, and I don't know if that is healthy but that is not my decision that is a political decision that effectively to say that this is a municipal area, these are the spots where you can trade and these are where you can't trade, these are the commodities we would like you to trade and then the other side is the training, the development education for traders and that's where Christa comes in, our law enforcement, we come in from business support we talk about financial viabilities we talk about having units like that.

P 4: SF Workshop_transcript.docx - 4:29 [We have a trading plan. A trad..]  (82:82)   (Super)
Codes:	[Trading plans] 
No memos

We have a trading plan. A trading plan normally depicts where trading takes place. So if you say Mitchells Plain, we will have a document that tells you how many traders there are, should be or is in M'Plain. And what the commodities are which they sell. That information is readily available with the new system that we have now you can manipulate that information because we moving onto a new … system, where you call for statistical data, which is also nice. 

P 4: SF Workshop_transcript.docx - 4:31 [I think the difficulty for us ..]  (84:84)   (Super)
Codes:	[Trading plans] 
No memos

I think the difficulty for us when we developing these trading plans are we not really specific about who is going to sell what. we just create the plan and put whoever is to go into that space. To find those people selling food is few and in-between. People sell everything else, I am talking about in a contextual structure of the trading plan process. There is a different story in the townships, in Khayelitsha, Langa there are no trading plans, it is free for all. We are still bringing a trading plan into that context. 

______________________________________________________________________

Code: Traffic bylaw {2-0}

P 2: Meeting EHManagers.doc - 2:5 [Yes, they drop them off at cer..]  (13:13)   (Super)
Codes:	[Cart_example] [Traffic bylaw] 
No memos

Yes, they drop them off at certain points and so on. And those things also had wheels on and I mean there were guys from law enforcement and they didn't mention anything about this unit has a wheel is going to be a problem or anything because it was various departments also the informal traders as well as people from the business sector and then the vendors themselves and it was us and law enforcement and we gave some input on there – on their design. I'm just looking at the… For the normal vendor maybe the affordability could be a bit of a challenge.

P 2: Meeting EHManagers.doc - 2:17 [so most of them most of those ..]  (44:44)   (Super)
Codes:	[Non-trading zone] [Traffic bylaw] 
No memos

so most of them most of those vendors the ones that are braaing the meat along the roadside they are not supposed to be there because those areas are not even zoned for economic purposes they are not for commercial purposes so they just forcing matters by being there and if things were to go the city's way the city could just remove all of them tomorrow or as in yesterday. So maybe that is why the colleagues from ED were kind of like cautious a bit skeptical towards… 

______________________________________________________________________

Code: Training offered {4-0}

P 3: Meeting_XXXXX.doc - 3:70 [Yes, we used to offer a blanke..]  (123:123)   (Super)
Codes:	[Training offered] 
No memos

Yes, we used to offer a blanket check an open check training but we find that the vendors didn't use that opportunity for whatever reason we didn't find out, so what we do now is that we have a number of I'm just trying to find the word, but we have a number of organizations who offer training opportunities and then what we do, vendors who are interested they tap into those training organizations. I'm just forgetting the name but I can give that to you as well.

P 3: Meeting_XXXXX.doc - 3:71 [It's not compulsory and then w..]  (125:125)   (Super)
Codes:	[Training offered] 
No memos

It's not compulsory and then what we do if there is an area where there is a general problem so say Christa and her staff pick up that there is – there is generally an issue of non compliance in an area, then in combination with environmental health and economic development we will go into that area and invite people for an awareness session.

P 3: Meeting_XXXXX.doc - 3:72 [I'm just thinking we had one i..]  (125:125)   (Super)
Codes:	[Training offered] 
No memos

I'm just thinking we had one in Athlone beginning of this year and it was very well received so there we had Christa there from her staff, we us from business support, we had law enforcement and sometimes in those training things we then introduce traders to new concepts. 

P 3: Meeting_XXXXX.doc - 3:73 [So training is available but i..]  (125:125)   (Super)
Codes:	[Training offered] 
No memos

So training is available but it is not compulsory. When the trader applies for a permit we normally at a very high level talk them through the rules and regulations and what the opportunities are.

______________________________________________________________________

Code: Transporting cart {1-0}

P 4: SF Workshop_transcript.docx - 4:3 [How is it going to be brought ..]  (7:7)   (Super)
Codes:	[Storage_challenge] [Transporting cart] 
No memos

How is it going to be brought to the trading space? In some cases people come via public transport. So they cannot travel with the unit plus other commodities with public transport. Storage is a huge issue. So if the storage can be solved a mobile unit could work.

______________________________________________________________________

Code: Urban agriculture {2-0}

P 3: Meeting_XXXXX.doc - 3:21 [You see one of the other acts ..]  (46:46)   (Super)
Codes:	[Urban agriculture] 
No memos

You see one of the other acts of the city it doesn't fall within my portfolio at all but it's the real desire to encourage urban agriculture and more on the fruit and vegetable side not so much the animal side. So we would go into our township communities and really encourage the information of fruit and vegetable garden and in some places it works and some places it doesn't work.

P 3: Meeting_XXXXX.doc - 3:22 [I was down at Dunoon the other..]  (46:46)   (Super)
Codes:	[Urban agriculture] 
No memos

 I was down at Dunoon the other day and under the power lines where you can't build houses because of electricity there's a vegetable garden so we encouraged that and that is food for the local community and that does work we kind of take it one step further down at Philippi we built fruit and vegetable I'm going to call it a market 

______________________________________________________________________

Code: Various departments involved {3-0}

P 2: Meeting EHManagers.doc - 2:16 [Then when it comes to the city..]  (44:44)   (Super)
Codes:	[Various departments involved] 
No memos

Then when it comes to the city when it comes to our scenario here in the city of Cape Town this is very it is a bit of a sensitive issue in a way and it is also quite broad because there are many parties involved there is health, there is economic development, there's traffic like you mentioned,

P 2: Meeting EHManagers.doc - 2:24 [Now, as you said for town plan..]  (48:48)   (Super)
Codes:	[Various departments involved] 
No memos

 Now, as you said for town planning or something else because already you see this thing with the wheels that I don't understand. So from my point of view I'm always trying to do the environmental side and don't put my neck into other departments because it is difficult. But, I will suggest that you do get those people on board if you haven't got yet get them on board. Fire as well for those safety things with the gas and stuff like that.

P 3: Meeting_XXXXX.doc - 3:10 [Let me finish talking about… T..]  (35:35)   (Super)
Codes:	[Bylaws of the city] [Policies vs legislation] [Various departments involved] 
No memos

Let me finish talking about… The city has got a policy its got its bylaw and what we did in terms of both the document took very much into account the 1991 document, so the backbone to our policies and bylaws and then also took into account other legislations around for example food trading. So when it comes to environmental health aspects and more specifically street trading and food, we don't specify in our bylaw just to say that any other bylaw that exists or any other legislation you need to comply with it and we had a huge debate in one sense and if you think from an informal trader perspective they don't want to have to go and search the entire legislative framework to find out what is applicable to them. And then the city we don't want to necessarily take one document and pull in from every other so you got environmental health legislation, you've got this legislation to do with solid waste your cleansing, you got traffic bylaws and all that that impacts on informal economy because then when you change your environmental health legislation you've got to remember you've got to change your informal trading bylaws so it was a debate and I don't think we settled that finally so we have said, Christa will talk about all the environmental health aspects and that embodied in their policies and their legislation. In the city's policy and bylaws we have just made reference to that you need to be aware of health issues and health and sanitation we don't specify…

______________________________________________________________________

Code: Vendor associations_challenge {1-0}

P 4: SF Workshop_transcript.docx - 4:36 [Can I just say, vendor associa..]  (103:103)   (Super)
Codes:	[Vendor associations_challenge] 
No memos

 Can I just say, vendor associations are actually one of the most challenging areas. Because you are going to have the Western Cape Coalition say that they represent the informal traders throughout South Africa. And they will aggressively tell you that. And as part of the SA trading forum. Yet our experiences shows that when you deal with traders on the ground some of them are members of the coalition and that's fine, but there are quite a few that are not. And they are quite resentful of the coalition representing themselves. Now the reason I say that as the city and very much so us and not top management deal with people on the ground, so we don't want to alienate that people because you happen to choose one particular organisation. So tto me, they in 2013, the city, the major had a informal trading summit. And we had a follow up earlier this year and now we are having ¾ micro summits and at the moment we are testing how we engage with the informal sector without alienating any particular body. It is a challenge. But I think the way we can do it; I mean we have a full house of colleagues, not so much vending associations, but individuals who happen to be members of associations. Try and identify them and then have a forum where we can get together. 


______________________________________________________________________

Code: Vendor challenges {12-0}

P 1: Interview with XXXXX.docx - 1:56 [I am not going to comment on t..]  (120:120)   (Super)
Codes:	[Vendor challenges] 
No memos

 I am not going to comment on that because we are not a service provision department. Other than the license and things like that. We are not like water services or economic development or planning or whatever the case may be, so I can see their frustration and things like that, all of those things are nice to have. It would be difficult to operate where you do not have electricity, and so on, but then there are people who are running successful little businesses. That has actually gone in from tiny, tiny starts to bigger things. And have become good caterers and things like that, so it is a constraint but it does not prevent people from trading.

P 1: Interview with XXXXX.docx - 1:57 [Of course it would be, but the..]  (122:122)   (Super)
Codes:	[Business license_homebase accepted as premises] [Vendor challenges] 
No memos

Of course it would be, but then we use the home premises as a base. So we accept that for a base. If it were to be close by t their stalls the people would look into it, but that would not be the main stumbling block for us not to give somebody a license. For a hawkers license we are happy with the home base premises. 

P 1: Interview with XXXXX.docx - 1:58 [Illegally in the sense that th..]  (126:126)   (Super)
Codes:	[Vendor challenges] 
No memos

Illegally in the sense that they are not trading from approved sights. You see that is the dinges. And in organising the informal industry the city probably has a lot of challenges. Because people think that they can just put their things up anywhere and the city is trying to clam in on that and make it more organised and control it for cleaning purposes and all sorts of things. And I know that that is a major thing so, the metro police I know do remove people form sites and that is probably where that criticism comes from. 

P 1: Interview with XXXXX.docx - 1:59 [Ja. And on top of one another,..]  (128:128)   (Super)
Codes:	[Vendor challenges] 
No memos

Ja. And on top of one another, so maybe there is not enough space for the dingese, so that is not something that affect us. 

P 1: Interview with XXXXX.docx - 1:60 [But I think they need to be ve..]  (130:130)   (Super)
Codes:	[Vendor challenges] [Waiting period_Application] 
No memos

But I think they need to be very careful and they need to be certain which things is it that they are waiting long for. Is it the permit for trade for a site? In other words a permit for the site or is it the permit for health? Because our turnaround times are, and I don't work with it anymore, but I don't have the sense that my colleagues take a hell of a long time to do it for hawkers. Because in terms of legislation that license should be ideally issued within 21 days. We give another 14 days to say 35 days. Now people sometimes have this perception that when they get into something like that, that we give them criticism when we give them requirements to meet before we can give them their license. And then they take 6 months to comply with those things, or they just never do and then they get a question like that, then all of a sudden it is the officials problem and not their problem. You see how it goes on.

P 3: Meeting_XXXXX.doc - 3:79 [That certainly has accepted th..]  (141:141)   (Super)
Codes:	[Vendor challenges] 
No memos

That certainly has accepted that knowledge and I think a challenge there from a city's perspective do you invest your money in creating markets where you can have access to water, electricity and sewerage and all that or do you rather create the opportunity on the streets so your Adderley Street or your main roads.

P 3: Meeting_XXXXX.doc - 3:90 [ou see there is a tension. let..]  (167:167)   (Super)
Codes:	[Allocated vs non-allocated zones] [Concession letters] [Vendor challenges] 
No memos

ou see there is a tension. let's just talk first of all about the 40% on municipal area that you don't need a location permit and just so that you can get a picture of it at the moment the Blouberg that whole area up the coast the whole of Khayelitsha there two pockets of Khayelitsha, but most of Khayelitsha town centre and then the area from Khayelitsha heading across towards Durbanville so that area is effectively a free trading area excluding the townships and the mentality of the community onto the politicians the ward councilor is that every single key informal trader is illegal so law enforcement will go there and the first thing is where is your permit. The fact that they talk to my staff and you don't need a permit the law says you don't need a permit but the mentality of law enforcement and the South African Police where's your permit and the trader gets very confused, I don't need a permit, but. So what we do and I'm only talking about the 40% of the area we issue what we call concession letters, its got no legal status it simply says you can trade these are the basic conditions and it is a piece of paper that the trader can get. Now, the councilors are very unhappy that we are issuing that letter and I have been challenged all over the place on it but I have succeeded with the challenge…

P 3: Meeting_XXXXX.doc - 3:91 [And there is no payment for it..]  (171:171)   (Super)
Codes:	[Vendor challenges] 
No memos

And there is no payment for it or that sort of thing. And then of course I'm not talking about the environmental health those are separate. In the 60% of the area because of the capacity constraints of law enforcement they tend to react to complaints so they would do their regular monitoring and inspections but they tend to react more to complaints and then because there is a complaint they are less sympathetic to the traders, so where is your permit – I don't have one, well you shouldn't be here and they come back in an hour's time and you still there then they will confiscate goods and that sort of thing they are less sympathetic. As I mentioned earlier on if you've got a permit and it's outdated we will give you a fine, but go and sort it out. The other challenge funny enough I've got a meeting this afternoon on it, it has to do with the fish traders now remember fish trade is they pick up the fish from the boats, put it on their bakkie and then they go and sell on the roadside now again most of that is within the no permit no trade areas, but yet the demand is there people want to buy.

P 3: Meeting_XXXXX.doc - 3:94 [What we try and do there is wh..]  (176:176)   (Super)
Codes:	[Security_crime_theft] [Vendor challenges] 
No memos

What we try and do there is where there are existing municipal facilities I use the word broadly, but for example where you've got a transport interchange, in any case at the facility there is security to look after the facility so they also address the crime and crime issue. In your established areas there are city improvement district or special rating areas where businesses contribute towards people that look after crime and grime but it is a real challenge and then the other thing that you haven't said there is a perception amongst to some communities that traders are directly responsible for the criminal element they are front for drugs, they fronts for money laundering etcetera, etcetera. They also are the eyes and ears of ATMs and they provide information and all that sort of thing. In some cases it does happen we know it happens but to a large extent perceptions of the community is not based on reality.

P 3: Meeting_XXXXX.doc - 3:97 [It is a valid comment we've un..]  (178:178)   (Super)
Codes:	[Vendor challenges] 
No memos

It is a valid comment we've undergone as a city undergone a process change initially it was literally a case of I'm just going to do Bellville as an example a trader wants to trade in Bellville they will go and see the area coordinator, fill in an application form, she will make sure that there is compliance and then she will issue the permit and you go to the cash office and pay for it. so it was literally as quick as that. There was a concern that it may the area coordinator is vulnerable in terms of favoritism so now there has been the separation, she would fill in the application form check the compliance as it were but she would make a recommendation to me and I need to make sure that she has complied that she followed the right policies guides. And then I would then sign it off and it goes through to my director he will then authorize it and then the information will come back where before he literally would walk in and walk out with the permit the delay now, best case scenario about two to three weeks. Sometimes my staff are guilty of not saying no upfront please, I will check I will come back to you. But when you have two or three hundred calls a month, how do you get back to two or three hundred people and it is sometimes very difficult you get people who are absolutely desperate to say upfront no. But, my word one needs to be honest.

P 3: Meeting_XXXXX.doc - 3:98 [The city policy on communicati..]  (180:180)   (Super)
Codes:	[Application process] [Vendor challenges] 
No memos

The city policy on communication that you need to communicate, you need to advise, you need to inform you shouldn't wait for more than seven days without some form of communication but I know the reality it's at least true. And then also, I think traders are opportunistic I had a lady phone me up and she said she applied for a permit eight years ago and she hasn't heard anything and my response to her I said sorry, I just struggle eight years is a long time, but 48 months ago surely when you apply and you haven't heard in a month or two months time you then… And if you still not getting an answer then talk to the senior escalator, eight yes no something is not right.

P 4: SF Workshop_transcript.docx - 4:13 [Maybe onsite visits to see how..]  (24:24)   (Super)
Codes:	[Site visits] [Vendor challenges] 
No memos

Maybe onsite visits to see how it works. At the moment we deal with historical traders who have ties with businesses. New comers struggle a bit in terms of storage etc.  

______________________________________________________________________

Code: Vendor education {2-0}

P 3: Meeting_XXXXX.doc - 3:31 [To me the approach would be tw..]  (57:57)   (Super)
Codes:	[Consumer education] [Vendor education] 
No memos

To me the approach would be two fold one is to educate the trader absolutely but one needs to also educate the community and it has taken me a long time to suddenly realize that what I am eating is unhealthy and most of the time I change my…

P 3: Meeting_XXXXX.doc - 3:44 [these are the commodities we w..]  (75:75)   (Super)
Codes:	[Vendor education] 
No memos

these are the commodities we would like you to trade and then the other side is the training, the development education for traders and that's where Christa comes in, our law enforcement, we come in from business support we talk about financial viabilities we talk about having units like that.

______________________________________________________________________

Code: Vendor initiave vs being prescriptive {6-0}

P 3: Meeting_XXXXX.doc - 3:16 [at the harbor end of Cape Town..]  (41:41)   (Super)
Codes:	[Vendor initiave vs being prescriptive] 
No memos

at the harbor end of Cape Town station there's a trader there that suddenly realized that there is a lot of focus on healthy food so what she has done it is a lady, she has now taken fruit and vegetables and taken a banana and a naartjie and packaged it that it is in a healthy display and people buy it. 

P 3: Meeting_XXXXX.doc - 3:17 [So she has got that niche, but..]  (41:41)   (Super)
Codes:	[Vendor initiave vs being prescriptive] 
No memos

So she has got that niche, but to me how far should a city go to be prescriptive in creating that niche. because we are not as a city we a legislative organ of state that fine line between being prescriptive and we are prescriptive in creating bylaws but then how far do you take it down in terms of what people buy and sell. 

P 3: Meeting_XXXXX.doc - 3:18 [My other thinking and it is a ..]  (41:41)   (Super)
Codes:	[Vendor initiave vs being prescriptive] 
No memos

My other thinking and it is a long way to get there is surely what we should be encouraging that if a trader wants to then sell healthy food stuff we should be encouraging them. But, we shouldn't say you must sell healthy food but to say if you train them in education these are the better alternatives these are the better options and we would encourage. 

P 3: Meeting_XXXXX.doc - 3:19 [And then if people then like t..]  (41:41)   (Super)
Codes:	[Vendor initiave vs being prescriptive] 
No memos

And then if people then like this lady who sells fruit and vegetables that is fine and there is another trader who is selling sushi that is fine, but again what do we do when we in some of your transport inter changes where the mindset of the trader is saying, I will sell what the commuters want.

P 3: Meeting_XXXXX.doc - 3:20 [The onions and the… I look at ..]  (43:43)   (Super)
Codes:	[Vendor initiave vs being prescriptive] 
No memos

The onions and the… I look at it and say no, but for that trader then to sell healthy food and nobody buys for whatever reason, and I just don't know that the city should be prescriptive in that environment.

P 3: Meeting_XXXXX.doc - 3:25 [And I'm struggling to do that,..]  (48:48)   (Super)
Codes:	[Vendor initiave vs being prescriptive] 
No memos

And I'm struggling to do that, that's why I say at the end of the day for me what would make sense how far do we go to become prescriptive as government on people in terms of their business model. Now, it's a philosophical debate but at the end of the day when a person doesn't succeed, is it the responsibility of the government or do we just say…

______________________________________________________________________

Code: Vendor involvement {1-0}

P 4: SF Workshop_transcript.docx - 4:23 [The other comment I wanted to ..]  (41:41)   (Super)
Codes:	[Vendor involvement] 
No memos

The other comment I wanted to make is there is an informal trading coalition, which has a very good slogan saying; “Nothing for us, without us”.  so maybe at the design stage to have them as part of that consultation. That we do not design something that won't suit them. It'll make it fall flat. 

______________________________________________________________________

Code: Vendors at clinics {1-0}

P 2: Meeting EHManagers.doc - 2:79 [Another sensitive area is your..]  (176:176)   (Super)
Codes:	[Vendors at clinics] 
No memos

 Another sensitive area is your clinics all of have got clinics in our areas and that is also same concepts happen there where the vendors are all around the fence and they sell because now the parent is waiting they wait in long queues so it's chips, it's koekies, it's Niknaks all of those things and that is carrying into the facility as well.

______________________________________________________________________

Code: Vendors at schools {3-0}

P 2: Meeting EHManagers.doc - 2:74 [he time when you took the… The..]  (167:167)   (Super)
Codes:	[Vendors at schools] 
No memos

he time when you took the… There is one of the areas that also needs to be addressed is whether it would be via an informal seller outside or because your whole purpose is to improve the nutrition value of what the food is of what the people take in kids especially. So maybe I don't know if it is outside this project, but you do have vendors that sell at the fence?

P 2: Meeting EHManagers.doc - 2:75 [So they would sell anything fr..]  (169:169)   (Super)
Codes:	[Vendors at schools] 
No memos

So they would sell anything from koeksisters to sweets to toffee apples to vetkoek to what… And for many of those kids parents don't give them they don't put in food for lunch so they would get money

P 2: Meeting EHManagers.doc - 2:76 [And that is their meal or thei..]  (171:171)   (Super)
Codes:	[Child nutrition] [Vendors at schools] 
No memos

And that is their meal or their lunch for the afternoon or whatever, so maybe that is one of the focus areas because that is a sensitive area. It's either I know one or two schools have done it before, but that is in the school where you would have your tuck shop to look at their menu in the tuck shop but also then combine that with the vendor that is outside. I know at your more affluent schools they don't allow vendors but if you look at the Delft areas Mannenberg areas those areas die aunties sit almal so they all sit on the road.

______________________________________________________________________

Code: Waiting period_Application {2-0}

P 1: Interview with XXXXX.docx - 1:37 [Waiting period. Could be one w..]  (91:91)   (Super)
Codes:	[Waiting period_Application] 
No memos

Waiting period. Could be one week, could be two, could be three, depending on how ready this person is to inspect it, ok. 

P 1: Interview with XXXXX.docx - 1:60 [But I think they need to be ve..]  (130:130)   (Super)
Codes:	[Vendor challenges] [Waiting period_Application] 
No memos

But I think they need to be very careful and they need to be certain which things is it that they are waiting long for. Is it the permit for trade for a site? In other words a permit for the site or is it the permit for health? Because our turnaround times are, and I don't work with it anymore, but I don't have the sense that my colleagues take a hell of a long time to do it for hawkers. Because in terms of legislation that license should be ideally issued within 21 days. We give another 14 days to say 35 days. Now people sometimes have this perception that when they get into something like that, that we give them criticism when we give them requirements to meet before we can give them their license. And then they take 6 months to comply with those things, or they just never do and then they get a question like that, then all of a sudden it is the officials problem and not their problem. You see how it goes on.

______________________________________________________________________

Code: Waste management {3-0}

P 2: Meeting EHManagers.doc - 2:80 [Did we include waste managemen..]  (196:196)   (Super)
Codes:	[Cart requirements] [Waste management] 
No memos

Did we include waste management?

P 2: Meeting EHManagers.doc - 2:81 [It's a big issue for us especi..]  (199:199)   (Super)
Codes:	[Cart requirements] [Waste management] 
No memos

It's a big issue for us especially what do you do with waste so you've got to try and build in some… A waste minimisation strategy with your thing because people don't like to take the waste home.

P 4: SF Workshop_transcript.docx - 4:19 [It was all built into the unit..]  (36:36)   (Super)
Codes:	[Waste management] 
No memos

It was all built into the unit. When he went to city health that is what he got. It has to be part of the trading operation. So it was built in. there was a place for washing hands and washing the fish. And waste disposal. And then at the end of the day a truck comes along, picks up the unit, takes it back to the factory, flushed/washed out and re-stocked. The next day it gets dropped off. 

______________________________________________________________________

Code: Waste water {2-0}

P 2: Meeting EHManagers.doc - 2:82 [So somehow that is also going ..]  (201:201)   (Super)
Codes:	[Waste water] 
No memos

So somehow that is also going to come into play. I think you touched on it Johan waste waters the grey water and what do we do with a person who is traveling maybe like we explained that a person – you might put a bicycle on this thing and make it a smaller version of that big thing. Now, you have that, so…

P 2: Meeting EHManagers.doc - 2:83 [To put it in a sewer system an..]  (204:204)   (Super)
Codes:	[Waste water] 
No memos

To put it in a sewer system and not in the storm water system that sort of thing that's why the base premises is so important in certain cases but it is not always practical as you said because his got to – that 20 litres is 20 kilograms so if you've got to cycle with that extra 20 kilograms they throw it here in the gutter.

______________________________________________________________________

Code: Weather_challenge {1-0}

P 4: SF Workshop_transcript.docx - 4:11 [in Cape Town a big issue is th..]  (7:7)   (Super)
Codes:	[Weather_challenge] 
No memos

 in Cape Town a big issue is the weather. If we look at the extremes, a black south easter where you have to anchor things down, or a really bad rainy winter north wester where rain comes in sideways. There it is that onw would go for the three sided gazebos. It is very practical. Sturdy. So we should look at what the unit is being aimed for, can it cope with the weather?

______________________________________________________________________
